# Genome-Wide Characterization of Snf1-Related Protein Kinases (SnRKs) and Expression Analysis of SnRK1.1 in Strawberry

**DOI:** 10.3390/genes11040427

**Published:** 2020-04-16

**Authors:** Yunting Zhang, Yuyun Ye, Leiyu Jiang, Yuanxiu Lin, Xianjie Gu, Qing Chen, Bo Sun, Yong Zhang, Ya Luo, Yan Wang, Xiaorong Wang, Haoru Tang

**Affiliations:** 1 College of Horticulture, Sichuan Agricultural University, Chengdu 611130, China; 2 Institute of Pomology and Olericulture, Sichuan Agricultural University, Chengdu 611130, China; 3 Mianyang Academy of Agricultural Sciences, Mianyang 621000, China

**Keywords:** SnRKs, phylogenetic analysis, expression pattern, strawberry

## Abstract

The plant sucrose nonfermenting 1 (SNF1)-related protein kinases (SnRKs) are key regulators in the interconnection of various signaling pathways. However, little is known about the SnRK family in strawberries. In this study, a total of 26 FvSnRKs including one FvSnRK1, nine FvSnRK2s and 16 FvSnRK3s were identified from the strawberry genome database. They were respectively designated as FvSnRK1.1, FvSnRK2.1 to FvSnRK2.9 and FvSnRK3.1 to FvSnRK3.16, according to the conserved domain of each subfamily and multiple sequence alignment with Arabidopsis. FvSnRK family members were unevenly distributed in seven chromosomes. The number of exons or introns varied among *FvSnRK1s*, *FvSnRK2s* and *FvSnRK3s*, but highly conserved in the same subfamily. The *FvSnRK1.1* had 10 exons. Most of *FvSnRK2s* had nine exons or eight introns, except *FvSnRK2.4*, *FvSnRK2.8* and *FvSnRK2.9*. *FvSnRK3* genes were divided into intron-free and intron-harboring members, and the number of introns in intron-harboring group ranged from 11 to 15. Moreover, the phylogenetic analysis showed SnRK1, SnRK2 and SnRK3 subfamilies respectively clustered together in spite of the different species of strawberry and Arabidopsis, indicating the genes were established prior to the divergence of the corresponding taxonomic lineages. Meanwhile, conserved motif analysis showed that FvSnRK sequences that belonged to the same subgroup contained their own specific motifs. Cis-element in promoter and expression pattern analyses of *FvSnRK1.1* suggested that *FvSnRK1.1* was involved in cold responsiveness, light responsiveness and fruit ripening. Taken together, this comprehensive analysis will facilitate further studies of the FvSnRK family and provide a basis for the understanding of their function in strawberry.

## 1. Introduction

Plants are forced to face the ever-changing surroundings during growth and development, due to their sessile nature. Thus, a timely response and coordinated regulatory machinery to external and internal signals is required, which helps plant to maintain optimal cellular conditions and resist various stresses originating from temperature, salinity, drought, nutrient defect and pathogen invasion [1,2]. It has been documented that these processes are involved in post-translation modifications (PTMs), such as phosphorylation, ubiquitination and acetylation. Arguably, the reversible protein phosphorylation executed by protein kinases and protein phosphatases plays a crucial role in intracellular signaling cascades and gene expression orchestration [3,4]. 

The sucrose non-fermenting 1 (SNF1) protein kinases that belong to Ser/Thr protein kinase are evolutionarily conserved in organisms. In plants, SNF1-related protein kinases (SnRKs) have been classified into SnRK1, SnRK2 and SnRK3 subfamilies on the basis of the sequence similarity and gene structures, which play key roles in integrating the metabolic and stress signaling [5]. The SnRK1 subfamily, which mainly participates in the global regulation of carbon and nitrogen metabolism, homologous to SNF1 genes in yeast and AMP-activated protein kinases (AMPKs) in mammals, functions as the heterotrimeric complex consisting of a catalytic α subunit and accessory β and γ subunits. The catalytic α subunit contains a highly conserved N-terminal catalytic domain and a variable C-terminal regulatory domain, which is important to form a complex interaction (with the β and γ subunits) and regulates the kinase activity [6,7]. Unlike SnRK1s, the other two subfamilies (SnRK2s and SnRK3s) are unique in plants. It has been reported that the catalytic region of SnRK2s and SnRK3s is analogous to that of SNF1/AMPK-type kinases. They probably derive from the gene duplication of SnRK1s and then diverge rapidly during plant evolution to fulfil new roles that enable plants to develop networks that link stress and abscisic acid (ABA) signaling with metabolic signaling [5,8]. Members of the SnRK2 subfamily have kinase domain, ATP binding domain, serine/threonine active site and four N-fourteen sites [9,10]. The first SnRK2 cDNA clone (PKABA1) was identified from an ABA-treated wheat embryo cDNA library, which was induced by salinity, cold, dehydration and osmotic stresses [11,12]. Early research has demonstrated that SnRK2s mainly participate in countering abiotic stress. Recent evidence suggests that SnRK2s are multifarious players in various biological processes like plant growth and development [13]. SnRK3s are known as calcineurin B-like calcium sensor-interacting protein kinases (CIPKs) that include a binding site for calcium-binding proteins (SOS3, salt overly sensitive 3; SCaBPS, SOS3-like calcium-binding proteins) and calcium-sensitive CBL (calcineurin B-like proteins) in the C-terminal region, which combine together to activate the protein kinase [14,15]. The CBL-CIPK calcium signaling network enables information integration and physiological coordination in response to a variety of extracellular cues [16].

In the light of the significant influence of SnRKs on regulating the metabolic and stress signaling, we identified SnRK members from strawberry and analyzed their conserved motif, homology and phylogenetic relationship with Arabidopsis. Especially, the SnRK1 kinases act as important metabolic sensors, which integrate diverse stress conditions and maintain energy homeostasis by direct phosphorylation of key metabolic enzymes and regulatory proteins, and massive transcriptional reprogramming [7,17,18]. Hence, we detected SnRK1 expression profiles in different strawberry tissues and under various abiotic stresses, which provided a theoretical basis for the genetic improvement of stress resistance and fruit quality.

## 2. Materials and Methods

### 2.1. Plant Materials and Treatments

Different tissues including root, stem, leaf, stolon, flower and berry at seven developmental stages (small green, SG; large green, LG; fade green, FG; white, W; turning red; TR; half red; HR; full red, FR) of the strawberry (*Fragaria×ananassa* cv. Benihoppe) were sampled. In addition, the Toyonoka strawberry cultivar was subjected to different light quality and low temperature treatment. The potted strawberries at the 7th day after flowering (7 DAF) were divided into four groups and then respectively transferred into growth chambers installed light emitting diodes of white (control), red (730 nm), blue (450 nm) and mixed light (red: blue = 1:1). The environmental conditions in chambers were strictly controlled (8 h dark at 16 °C, 16 h photoperiod at 25 °C, 100 μmol∙m^−2^∙s^−1^ light intensity and 75% relative humidity). Fruits were harvested at the 14th, 21th, 25th, 28th day after flowering. In addition, two groups of the potted strawberries at white stage of fruit were put into 4 °C (cold) and 25 °C (control) chambers under a 16 h diurnal light cycle at 100 μmol∙m^−2^∙s^−1^ with 75% relative humidity for low temperature treatment, respectively. Fruits were sampled at 0, 6, 12, 24, 48, and 72 h. All of above samples were used to investigate *SnRK1.1* expression pattern. 

### 2.2. Identification and Characterization of FvSnRKs 

The *Arabidopsis* SnRK proteins (Supplementary File S1) obtained from TAIR database (https://www.arabidopsis.org/) were used as query probes to BLAST search against *Fragaria vesca* v1.0 genome database (https://www.rosaceae.org). Meanwhile, the whole protein sequences of *F. vesca* were downloaded from this database. Then, the Hidden Markov Model (HMM) files corresponding to each subfamily of SnRKs were downloaded from Pfam protein family database (http://pfam.sanger.ac.uk/), and HMMER 3.0 was used to search the SnRKs from the protein sequence dataset with the default threshold. To further ensure the existence of SnRK conserved structures, each candidate protein based on results of BLAST search and HMM search was checked using the NCBI-CDD (https://www.ncbi.nlm.nih.gov/) and SMART (http://smart.embl-heidelberg.de/) online tools. 

### 2.3. Sequence Analysis of FvSnRKs

The information of all SnRK proteins, genes, coding sequences (CDS) was obtained from the strawberry genome database (Supplementary File S2–S4). The protein theoretical pI, molecular weight (MW), aliphatic index, grand average of hydropathicity (GRAVY) and instability index were evaluated using the ExPASy-ProtParam tool (http://web.e xpasy.org/protparam/). Conserved motifs of the FvSnRKs were identified using the Multiple EM for Motif Elicitation (MEME) web server (http://meme.nbcr.net/meme/cgi-bin/meme.cgi) and visualized using TBtools [19]. Putative signal peptide and transmembrane helix (TMH) were predicted in the SignalP 4.1 Server (http://www.cbs.dtu.dk/services/SignalP/) and the TMHMM Server v.2.0 (http://www.cbs.dtu.dk/ser vices/TMHMM/), respectively. Subcellular location was speculated by ProtComp v.9.0 (http://linux1.softberry.com/berry.phtml?topic=protc omppl&group=programs&subgroup=proloc). Additionally, the cis-element of *FvSnRK1.1* promoter was analyzed in the Plantcare database (http://bioinformatics.psb.ugent.be/webtools/plantcare/html/). 

### 2.4. Gene Structure and Phylogenetic Tree of FvSnRKs

The exon–intron structure of *FvSnRK* genes was analyzed using the Gene Structure Display Server program (GSDS v.2.0, http://gsds.cbi.pku.edu.cn) based on the comparison of coding sequences (Supplementary File S4) and corresponding genomic sequences (Supplementary File S3). The unrooted phylogenetic tree of FvSnRK proteins was constructed in MEGA v.6.0 according to the neighbor-joining (NJ) method with Poisson model and 1000 bootstrap replications, after multiple sequence alignment was performed in ClustalX v.2.0 software. Then the classification of FvSnRKs into subfamilies was based on the previous report in Arabidopsis. 

### 2.5. RNA Extraction and Gene Expression of FvSnRKs

Total RNA was isolated from all samples using the improved CTAB method [20]. After quality and quantity were checked by 1% agrose gel electrophoresis and BioPhotometer, 1 μg of total RNA was used for reverse-transcription into the first strand of cDNA with PrimeScript TM RT reagent Kit with gDNA Eraser (Perfect Real Time) (Takara, Japan) according to the operating manual. All quantitative real-time PCRs were performed on the CFX96 real-time PCR system (Bio-Rad, USA). A total volume of 10 μL reaction mixture contained 0.4 μL of each primer (10 μM), 1 μL of 10-fold dilution of cDNA, 3.2 μL of RNase-free water and 5 μL of SYBR Premix (Takara, Japan). The reaction procedure was carried out as follows: 95 °C/3 min for pre-denaturation, followed by 40 cycles of 95 °C /10 s for denaturation, 60 °C/30 s for annealing and 72 °C/15 s for extension. Subsequently, melting curve was executed to confirm specificity of primers, ramping from 65 °C to 95 °C (increment 0.5 °C/5 s). The relative expression level of *FvSnRK1.1* to 26S-18S RNA housekeeping gene was analyzed with the 2^−ΔΔCT^ method. The primers were as follows: 5’-GCACAAATGGTTCCAGGCTC-3’ (sense) and 5’-GAAACTCGGCCCCAAGGTAG-3’ (antisense) for *FvSnRK1.1* 239 bp amplicon; 5’- ACCGTTGATTCGCACAATTGGTCATCG-3’ (sense) and 5’- TACTGCGGGTCGGCAATCGGACG -3’ (antisense) for 26S-18S RNA housekeeping gene 150 bp amplicon. 

## 3. Results

### 3.1. Identification and Properties of SnRKs in F. versa

To extensively screen the SnRKs from the strawberry genome database, two methods, including BLAST search and HMM search, were applied. Eventually, 26 FvSnRKs composed of one FvSnRK1, nine FvSnRK2s and 16 FvSnRK3s were identified. They were respectively designated as FvSnRK1.1, FvSnRK2.1 to FvSnRK2.9 and FvSnRK3.1 to FvSnRK3.16, according to conserved domain and multiple sequence alignment with Arabidopsis. Their distribution and sequence feature were analyzed in Table 1. FvSnRK1.1 was distributed on chromosome 6. FvSnRK2s were scattered to chromosome 1, 2, 4 and 5. FvSnRK3s were distributed over all chromosomes except chromosome 5. Respectively, the open reading frame (ORF) length of FvSnRK1.1, FvSnRK2s and FvSnRK3s was 1557 bp, 963–1215 bp and 1284–1524 bp, and deduced protein was 518 aa, 320–404 aa and 427–507 aa, and the relative molecular mass was 59.19 kDa, 36.30–45.72 kDa and 47.96–57.42 kDa. The FvSnRK1.1 protein was predicted to be unstable, while most of the FvSnRK3s were stable. All of the FvSnRKs had no signal peptide and only FvSnRK3.11 contained one transmembrane helix. Subcellular localization prediction indicated FvSnRK1.1 and FvSnRK2s were localized to the cytoplasm and nucleus, whereas FvSnRK3s were localized to cytoplasm, nucleus, endoplasmic reticulum or membrane.

### 3.2. Analysis of Gene Structure

**Figure 1:**
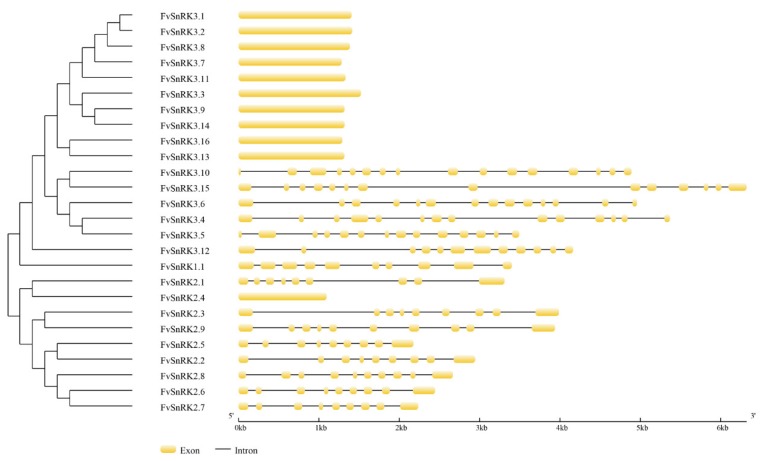
Gene structure analysis of *FvSnRKs*. Exon–intron structure was analyzed in the Gene Structure Display Server (GSDS) program. The yellow boxes indicate exons, and the gray horizontal lines indicate introns. The scale bar represents 6 kb.

The *FvSnRKs* exon–intron organizations were analyzed in GSDS. As shown in Figure 1, the *FvSnRK1.1* coding sequence was composed of 10 exons. Most of the *FvSnRK2s* had nine exons or eight introns, except *FvSnRK2.4*, *FvSnRK2.8* and *FvSnRK2.9*. The order and approximate size of exons among the *FvSnRK2s* were relatively conserved, but the size of introns was variable, which gave rise to a diversity of gene structures. Clearly, *FvSnRK3s* can be divided into three clades according to the exon–intron of gene structures. The first clade included ten genes without introns. The second clade had five members. Of these, four members contained 14 exons, while the remaining one contained 16 exons. The third clade contained only one gene, *FvSnRK3.12,* with 12 exons.

### 3.3. Phylogenetic Analysis and Conserved Motifs of FvSnRKs

**Figure 2:**
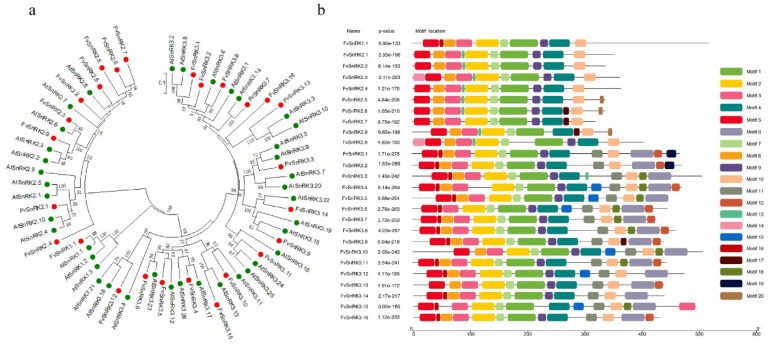
Phylogenetic relationships among SnRKs between strawberry and Arabidopsis (**a**) and conserved motifs of FvSnRKs (**b**). The evolutionary tree was constructed with the neighbor-joining method based on the Poisson model using 1000 bootstrap replicates. The green and red dots respectively indicate Arabidopsis and strawberry SnRK proteins. Conserved motifs of FvSnRKs in strawberry were analyzed using the MEME web server. Different color boxes represent different types of putative motifs.

To gain insight into the evolutionary relationship among SnRKs between the strawberry and Arabidopsis, a phylogenetic tree was constructed based on the multiple alignment of the amino acid sequences. All SnRKs were specifically classified into three main clades of SnRK1, SnRK2 and SnRK3 subfamilies (Figure 2a). The results showed FvSnRK1.1 was clustered together with AtSnRK1.1, AtSnRK1.2 and AtSnRK1.3. FvSnRK2s were divided into three subgroups. Two subgroups respectively contained one member FvSnRK2.1 and FvSnRK2.4, and the third one contained the rest seven members. FvSnRK3s were compartmentalized into three subgroups that respectively included 1, 5 and 10 members. Twenty conserved motifs of 26 FvSnRKs were identified using the online MEME program (Supplementary File S5). Clearly, all of SnRKs in the strawberry shared some motifs, but SnRK1, SnRK2 and SnRK3-class had their own idiographic characteristic (Figure 2b). 

### 3.4. Cis-element Analysis in the Promoter Region of FvSnRK1.1 Gene

The 2000 bp fragment of upstream region from the start codon in *FvSnRK1.1* gene was isolated to predict the cis-acting regulatory element. As shown in Table 2, the *FvSnRK1.1* promoter included several light responsive elements, such as AE-box, Box 4, G-box, GATA-motif, GT1-motif, L-box, TCT-motif and MRE. It also contained stress-related (anaerobic, drought, low temperature, etc.) cis-elements (ARE, LTR, MBS, TC-rich repeats), hormone-related (abscisic acid, MeJA, gibberellin, salicylic acid) cis-elements (ABRE, CGTCA-motif, P-box, TCA-element, TGACG-motif). Moreover, it was involved in endosperm expression, zein metabolism regulation. In addition, this region existed many core promoter TATA-box and enhancer CAAT-box.

### 3.5. Expression Pattern of FvSnRK1.1 in Different Tissues and During the Fruit Development

**Figure 3:**
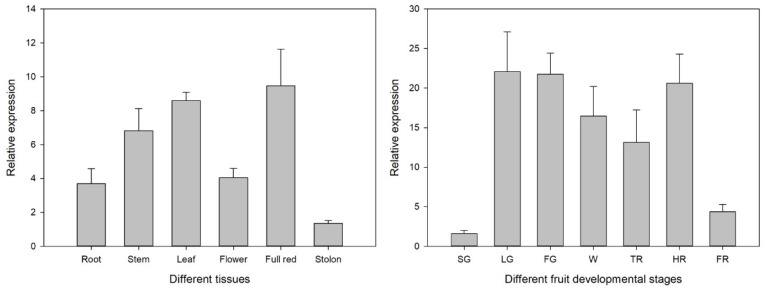
Expression level of *FvSnRK1.1* in different tissues and during the fruit development. SG, small green; LG, large green; FG, fade green; W, white; TR, turning red; HR, half red; FR, full red.

As shown in Figure 3, *FvSnRK1.1* constitutively expressed among different tissues. It had much higher expression level in fruit, leaf and stem, followed by flower and root, and had lowest transcript abundance in stolon. To investigate whether the expressions of the *FvSnRK1.1* in strawberries were associated with fruit development, its expression pattern in different samples of small green (SG), large green (LG), fade green (FG), white (W), turning red (TR), half red (HR) and full red (FR) was analyzed by qRT-PCR (quantitative real-time PCR). The expression level of *FvSnRK1.1* had a remarkable increase from SG to LG. After this, it kept high transcript abundance except in the FR stage. These results indicated that expression of *FvSnRK1.1* was related to early strawberry fruit development and might play an important role in regulating fruit ripening.

### 3.6. Expression Pattern of FvSnRK1.1 in Response to Different Treatment

**Figure 4:**
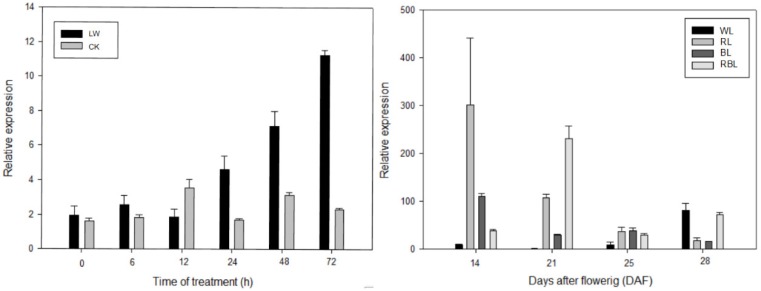
Expression level of *FvSnRK1.1* under low temperature and light quality treatment. LW, low temperature; WL, white light; RL, red light; BL, blue light; RBL, mixed light (red: blue = 1:1).

*SnRK1s* was confirmed to participate widely in response to various environmental elicitors. The expression pattern of the *FvSnRK1.1* gene under low temperature and light quality treatments were thus examined. The results showed that *FvSnRK1.1* was highly induced after the 12-hour low temperature exposure. Additionally, the transcript level of *FvSnRK1.1* had different sensitivities to white, red, blue and red/blue light. It was significantly upregulated by RL, BL and RBL from 14 to 25 DAF. Furthermore, the *FvSnRK1.1* expression pattern differed under different light quality during the fruit development. It had a gradually decreasing trend under red and blue light treatment, while an opposite tendency existed under white light (Figure 4).

## 4. Discussion

SnRKs as protein kinases represent an interface between metabolic and stress signaling pathways in plants. SnRK1 is closely related to the metabolic regulators of mammals (5’-AMP-activated protein kinase, AMPK) and yeast (sucrose non-fermenting-1, SNF1), with which it shares about 47% amino acid sequence identity and similar substrate specificity. As the plant has evolved, the subfamilies SnRK2 and SnRK3 have emerged, which are larger and relatively more diverse compared with SnRK1 [8]. Bioinformatic analysis of SnRK family has isolated a total of 39 AtSnRKs including three AtSnRK1s, 10 AtSnRK2s and 26 AtSnRK3s in *Arabidopsis* [21,22,23,24], 44 BdSnRKs including three BdSnRK1s, 10 BdSnRK2s and 31 BdSnRK3s in *Brachypodium distachyon* [25], 34 EgrSnRK including two EgrSnRK1s, eight EgrSnRK2s and 24 EgrSnRK3s in *Eucalyptus grandis* [26]. Here, we identified 26 FvSnRKs composed of one FvSnRK1, nine FvSnRK2s, 16 FvSnRK3s in the wild strawberry (*Fragaria vesca*). Overall, the SnRK3 subfamily has the largest number of members, while the SnRK1 subfamily has the fewest members. In our study, the consequences of sequence feature, subcellular localization, gene structure and phylogeny analysis also indicated the function diversity of SnRKs. 

Subcellular localization showed SnRK3s were localized to endoplasmic reticulum and membranes besides cytoplasm and nuclei, indicating it functions in more cellular compartments. The diversification of the exon–intron structure played a pivotal role in the evolution and function of many gene families. The number of exons or introns varied in different subfamilies and genes. The *FvSnRK1.1* had 10 exons, which was the same as that reported for *EgrSnRK1s* and *BdSnRK1s* [25,26]. Most of *FvSnRK2s* had nine exons or eight introns, except *FvSnRK2.4*, *FvSnRK2.8* and *FvSnRK2.9*, which are consistent with that of most Arabidopsis, rice, maize, sorghum, tea and grape *SnRK2* genes [27,28,29,30], suggesting that most *SnRK2s* in plants have a conserved structure with nine exons or eight introns. Like the *SnRK3s* subfamily in other plant species, *SnRK3* genes in strawberries were also divided into intron-free and intron-harboring members, and the number of introns in intron-harboring group ranged from 11 to 15. These findings have showed that the number of *SnRKs* exon–intron exhibits high conservation during the evolution in each subfamily. Moreover, the phylogenetic analysis showed SnRK1, SnRK2 and SnRK3 respectively clustered together in spite of the different species of strawberry and Arabidopsis, indicating that the genes were established prior to the divergence of the corresponding taxonomic lineages. Meanwhile, conserved motif analyses showed that SnRK sequences that belonged to the same subgroup contained the specific motifs.

The SnRK1 kinases influence plant metabolism, stress tolerance, and a large array of growth and developmental processes by direct phosphorylation of regulatory proteins and metabolic enzymes, and by extensive transcriptional orchestration [31,32,33]. To better understand the function and regulation of *SnRK1* in strawberries, the *FvSnRK1.1* promoter sequence was analyzed using PlantCARE tool. The results showed that it contained a series of cis-elements involved in stress-responsiveness, hormone-responsiveness and light responsiveness, indicating that *FvSnRK1.1* has a diverse role in the strawberry. Clearly, *FvSnRK1.1* indeed responded to low temperatures according to their up-regulating expression pattern, which corroborated the result in *FvSnRK1.1* promoter analysis. It was reported that SnRK1 could interact with a trihelix family gene *ShCIGT* to mediate cold tolerance in a tomato [34]. Meanwhile, the *FvSnRK1.1* transcript level changed under different light quality treatment, indicating it was sensitive to light signal. The Arabidopsis *akinβ1*(a subunit of SnRK1) mutant altered multiple genes expression level in response to light and dark [33]. In addition, *FvSnRK1.1* transcript accumulated in tissue-specific manner. It has been documented that overexpression of *SnRK1* could increase the fruit starch and soluble sugar and promote fruit ripening [35,36,37]. For instance, a tomato overexpressing PpSnRK1α caused higher sugar content and matured approximately 10 days earlier than the WT. It was demonstrated that PpSnRK1α interacted with the MADS-box transcription factor SIRIN, increasing the expression of RIN, regulating the expression of downstream ripening-related genes and promoting the fruit ripening [37]. Here, the high expression level of *FvSnRK1.1* during fruit development suggested that *FvSnRK1.1* was probably involved in strawberry fruit maturation, but the detailed mechanism requires in-depth study. 

In conclusion, we isolated and characterized 26 SnRKs including one SnRK1, nine SnRK2s and 16 SnRK3s from the strawberry genome and analyzed their gene structure, conserved protein motif and evolutionary relationship, which will supply abundant information for functional investigation of SnRK genes. Cis-elements in promoter and expression patterns in response to external signals analysis suggested that *FvSnRK1.1* was involved in cold responsiveness and light responsiveness. The expression profiles of *FvSnRK1.1* in distinct tissues, stages of fruit development and ripening indicated that the *FvSnRK1.1* transcript accumulation was tissue-specific and possibly related to strawberry fruit maturation. These results may advance the understanding of the role of *FvSnRK1.1* in the regulation of strawberry development and responses to abiotic stress. 

## Figures and Tables

**Table 1:** Sucrose nonfermenting 1 (SNF1)-related protein kinases **(***SnRKs*) genes and corresponding protein properties in strawberry.

Gene Name	Gene Id	Chr.	Locus	ORF (bp)	Amino Acid (aa)	MW (kDa)	pI	Instability Index	Aliphatic Index	GRAVY	SignalP	TMH	Location
*FvSnRK1.1*	gene04397	chr6	32622471..32625872+	1557	518	59.19	8.68	48.23 (unstable)	88.78	−0.313	no	0	a, b
*FvSnRK2.1*	gene11031	chr5	16681534..16684843+	1059	352	40.40	6.12	44.84 (unstable)	82.78	−0.511	no	0	a, b
*FvSnRK2.2*	gene10769	chr5	23595280..23598224+	1014	337	38.04	5.85	35.42 (stable)	86.20	−0.399	no	0	a, b
*FvSnRK2.3*	gene31902	chr2	12574424..12578412+	1092	363	41.23	4.88	41.24 (unstable)	88.35	−0.316	no	0	a, b
*FvSnRK2.4*	gene06595	chr4	14755025..14756119+	1095	364	41.48	9.10	45.92 (unstable)	82.17	−0.429	no	0	a, b
*FvSnRK2.5*	gene16244	chr1	13571546..13573722+	1014	337	38.38	5.18	33.76 (stable)	90.50	−0.286	no	0	a, b
*FvSnRK2.6*	gene11971	chr5	12304605..12307049−	1014	337	38.10	5.76	36.88 (stable)	89.91	−0.242	no	0	a, b
*FvSnRK2.7*	gene11970	chr5	12295362..12297597−	963	320	36.30	8.76	37.85 (stable)	91.34	−0.253	no	0	a, b
*FvSnRK2.8*	gene11969	chr5	12290219..12292886−	1050	349	39.43	6.76	41.81 (unstable)	81.20	−0.342	no	0	a, b
*FvSnRK2.9*	gene24096	chr1	11643335..11647273+	1215	404	45.72	4.82	44.09 (unstable)	90.20	−0.170	no	0	a, b
*FvSnRK3.1*	gene28136	chr3	21779588..21780994−	1407	468	53.65	8.77	40.89 (unstable)	88.12	−0.491	no	0	c, d
*FvSnRK3.2*	gene13841	chr6	7167764..7169179+	1416	471	53.28	9.00	32.77 (stable)	89.36	−0.414	no	0	c, d
*FvSnRK3.3*	gene29682	chr3	7803967..7805490−	1524	507	56.29	7.64	39.44 (stable)	88.22	−0.219	no	0	c, d
*FvSnRK3.4*	gene15372	chr2	32504181..32509548+	1416	471	53.98	6.03	32.57 (stable)	88.58	−0.334	no	0	a, b
*FvSnRK3.5*	gene18646	chr7	3032869..3036362−	1347	448	51.53	9.13	40.50 (unstable)	77.46	−0.539	no	0	a, b
*FvSnRK3.6*	gene30382	chr3	2915121..2920078+	1341	446	49.55	8.83	30.14 (stable)	83.30	−0.314	no	0	a, b
*FvSnRK3.7*	gene10067	chr1	842428..843711−	1284	427	47.96	9.34	28.34 (stable)	83.58	−0.326	no	0	c, d
*FvSnRK3.8*	gene29681	chr3	7799332..7800717+	1386	461	51.94	9.11	31.99 (stable)	78.87	−0.429	no	0	c, d
*FvSnRK3.9*	gene13849	chr6	7132323..7133642−	1320	439	49.58	7.58	31.35 (stable)	91.69	−0.266	no	0	a, b, d
*FvSnRK3.10*	gene31049	chr1	3172739..3177629−	1524	507	57.42	9.24	43.34 (unstable)	86.37	−0.286	no	0	a, b, d
*FvSnRK3.11*	gene15015	chr2	35278943..35280274−	1332	443	49.74	8.94	38.64 (stable)	77.24	−0.346	no	1	c, d
*FvSnRK3.12*	gene22806	chr4	15674061..15678224+	1425	474	53.06	8.06	44.47 (unstable)	93.54	−0.368	no	0	a, b
*FvSnRK3.13*	gene29066	chr4	6439716..6441032+	1317	438	47.51	8.87	35.28 (stable)	94.86	−0.080	no	0	a, b
*FvSnRK3.14*	gene28132	chr3	21757318..21758637+	1320	439	49.48	8.52	38.61 (stable)	87.40	−0.298	no	0	a, b, d
*FvSnRK3.15*	gene26443	chr1	4822218..4828540−	1485	494	55.46	5.67	37.29 (stable)	93.30	−0.074	no	0	a, b, d
*FvSnRK3.16*	gene21319	chr7	19497585..19498877+	1293	430	48.84	8.99	30.50 (stable)	87.09	−0.328	no	0	c, d

Note: a, cytoplasm; b, nucleus; c, endoplasmic reticulum; d, membrane.

**Table 2:** The cis-acting regulatory element in the promoter of *FvSnRK1.1.*

Function Class	Cis-Elements	Amount	Function
Stress	ARE	3	essential for anaerobic induction
	LTR	1	low-temperature responsiveness
	MBS	1	MYB binding site involved in drought-inducibility
	TC-rich repeats	3	defense and stress responsiveness
Hormone	ABRE	1	abscisic acid responsiveness
	CGTCA-motif	3	MeJA-responsiveness
	P-box	1	gibberellin-responsive element
	TCA-element	1	salicylic acid responsiveness
	TGACG-motif	3	MeJA-responsiveness
Others	AE-box	3	part of a module for light response
	Box 4	2	light responsiveness
	G-box	3	light responsiveness
	GATA-motif	2	part of a light responsive element
	GT1-motif	3	light responsive element
	L-box	1	part of a light responsive element
	TCT-motif	2	part of a light responsive element
	MRE	3	MYB binding site involved in light responsiveness
	GCN4_motif	1	endosperm expression
	O2-site	1	zein metabolism regulation

## Supplementary Materials

The following are available online at https://www.mdpi.com/2073-4425/11/4/427/s1, Supplementary File S1: Protein sequences of Arabidopsis SnRK genes; Supplementary File S2: Protein sequences of strawberry SnRK genes; Supplementary File S3: Genomic sequences of strawberry SnRK genes; Supplementary File S4: Coding sequences of strawberry SnRK genes; Supplementary File S5: Conserved motifs of strawberry SnRK proteins.
