# Supplementary material for: Genome-Wide Characterization of Snf1-Related Protein Kinases (SnRKs) and Expression Analysis of SnRK1.1 in Strawberry
Source: Genes (Basel). 2020 Apr 16;11(4):427. doi: 10.3390/genes11040427 (PMC7230852; doi:10.3390/genes11040427)
Supplement: Supplementary file 1 [file genes-11-00427-s001.zip › Supplementary File S1.docx]

Supplementary File S1: Protein sequences of Arabidopsis SnRK genes.

>AtSnRK1.1(At3g01090)

MFKRVDEFNLVSSTIDHRIFKSRMDGSGTGSRSGVESILPNYKLGRTLGIGSFGRVKIAEHALTGHKVAIKILNRRKIKNMEMEEKVRREIKILRLFMHPHIIRLYEVIETPTDIYLVMEYVNSGELFDYIVEKGRLQEDEARNFFQQIISGVEYCHRNMVVHRDLKPENLLLDSKCNVKIADFGLSNIMRDGHFLKTSCGSPNYAAPEVISGKLYAGPEVDVWSCGVILYALLCGTLPFDDENIPNLFKKIKGGIYTLPSHLSPGARDLIPRMLVVDPMKRVTIPEIRQHPWFQAHLPRYLAVPPPDTVQQAKKIDEEILQEVINMGFDRNHLIESLRNRTQNDGTVTYYLILDNRFRASSGYLGAEFQETMEGTPRMHPAESVASPVSHRLPGLMEYQGVGLRSQYPVERKWALGLQSRAHPREIMTEVLKALQDLNVCWKKIGHYNMKCRWVPNSSADGMLSNSMHDNNYFGDESSIIENEAAVKSPNVVKFEIQLYKTRDDKYLLDLQRVQGPQFLFLDLCAAFLAQLRVL

>AtSnRK1.2(At3g29160)

MDHSSNRFGNNGVESILPNYKLGKTLGIGSFGKVKIAEHVVTGHKVAIKILNRRKIKNMEMEEKVRREIKILRLFMHPHIIRQYEVIETTSDIYVVMEYVKSGELFDYIVEKGRLQEDEARNFFQQIISGVEYCHRNMVVHRDLKPENLLLDSRCNIKIADFGLSNVMRDGHFLKTSCGSPNYAAPEVISGKLYAGPEVDVWSCGVILYALLCGTLPFDDENIPNLFKKIKGGIYTLPSHLSSEARDLIPRMLIVDPVKRITIPEIRQHRWFQTHLPRYLAVSPPDTVEQAKKINEEIVQEVVNMGFDRNQVLESLRNRTQNDATVTYYLLLDNRFRVPSGYLESEFQETTDSGSNPMRTPEAGASPVGHWIPAHVDHYGLGARSQVPVDRKWALGLQSHAHPREIMNEVLKALQELNVCWKKIGHYNMKCRWVPGLADGQNTMVNNQLHFRDESSIIEDDCAMTSPTVIKFELQLYKAREEKYLLDIQRVNGPQFLFLDLCAAFLTELRVI

>AtSnRK1.3(At5g39440)

MDGSSEKTTNKLVSILPNYRIGKTLGHGSFAKVKLALHVATGHKVAIKILNRSKIKNMGIEIKVQREIKILRFLMHPHIIRQYEVIETPNDIYVVMEYVKSGELFDYIVEKGKLQEDEARHLFQQIISGVEYCHRNMIVHRDLKPENVLLDSQCNIKIVDFGLSNVMHDGHFLKTSCGSPNYAAPEVISGKPYGPDVDIWSCGVILYALLCGTLPFDDENIPNVFEKIKRGMYTLPNHLSHFARDLIPRMLMVDPTMRISITEIRQHPWFNNHLPLYLSIPPLDTIDQAKKIEEEIIQNVVNIGFDRNHVVDSLANRIQNEATVAYHLILDNRNQNSVPNDPFQSKFKEISDGIFNSTLPVQNITSHVGHSFSALYGLKSNVKDDKTWTLGLQSQGSPYDIMTEIFKALQNLKICWKKIGLYNIKCRWVRSFAYYKNHTIEDECAIILPTVIKFEIQLYKVREGKYLLDILRIDGPQFIFFDLCVAFLRELGVL

>AtSnRK2.1(At5g08590)

MDKYDVVKDLGAGNFGVARLLRHKDTKELVAMKYIERGRKIDENVAREIINHRSLKHPNIIRFKEVILTPTHLAIVMEYASGGELFDRICTAGRFSEAEARYFFQQLICGVDYCHSLQICHRDLKLENTLLDGSPAPLLKICDFGYSKSSILHSRPKSTVGTPAYIAPEVLSRREYDGKHADVWSCGVTLYVMLVGAYPFEDPNDPKNFRKTIQRIMAVQYKIPDYVHISQECKHLLSRIFVTNSAKRITLKEIKNHPWYLKNLPKELLESAQAAYYKRDTSFSLQSVEDIMKIVGEARNPAPSTSAVKSSGSGADEEEEEDVEAEVEEEEDDEDEYEKHVKEAQSCQESDKA

>AtSnRK2.2(At3g50500)

MDPATNSPIMPIDLPIMHDSDRYDFVKDIGSGNFGVARLMTDRVTKELVAVKYIERGEKIDENVQREIINHRSLRHPNIVRFKEVILTPSHLAIVMEYAAGGELYERICNAGRFSEDEARFFFQQLISGVSYCHAMQICHRDLKLENTLLDGSPAPRLKICDFGYSKVLFISLKSSVLHSQPKSTVGTPAYIAPEILLRQEYDGKLADVWSCGVTLYVMLVGAYPFEDPQEPRDYRKTIQRILSVTYSIPEDLHLSPECRHLISRIFVADPATRITIPEITSDKWFLKNLPGDLMDENRMGSQFQEPEQPMQSLDTIMQIISEATIPTVRNRCLDDFMADNLDLDDDMDDFDSESEIDVDSSGEIVYAL

>AtSnRK2.3(At5g66880)

MDRAPVTTGPLDMPIMHDSDRYDFVKDIGSGNFGVARLMRDKLTKELVAVKYIERGDKIDENVQREIINHRSLRHPNIVRFKEVILTPTHLAIIMEYASGGELYERICNAGRFSEDEARFFFQQLLSGVSYCHSMQICHRDLKLENTLLDGSPAPRLKICDFGYSKSSVLHSQPKSTVGTPAYIAPEVLLRQEYDGKIADVWSCGVTLYVMLVGAYPFEDPEEPRDYRKTIQRILSVKYSIPDDIRISPECCHLISRIFVADPATRISIPEIKTHSWFLKNLPADLMNESNTGSQFQEPEQPMQSLDTIMQIISEATIPAVRNRCLDDFMTDNLDLDDDMDDFDSESEIDIDSSGEIVYAL

>AtSnRK2.4(At1g10940)

MDKYELVKDIGAGNFGVARLMKVKNSKELVAMKYIERGPKIDENVAREIINHRSLRHPNIIRFKEVVLTPTHLAIAMEYAAGGELFERICSAGRFSEDEEEGNKRKHARYFFQQLISGVSYCHAMQICHRDLKLENTLLDGSPAPRLKICDFGYSKSSLLHSRPKSTVGTPAYIAPEVLSRREYDGKMADVWSCGVTLYVMLVGAYPFEDQEDPKNFRKTIQKIMAVQYKIPDYVHISQDCKNLLSRIFVANSLKRITIAEIKKHSWFLKNLPRELTETAQAAYFKKENPTFSLQTVEEIMKIVADAKTPPPVSRSIGGFGWGGNGDADGKEEDAEDVEEEEEEVEEEEDDEDEYDKTVKEVHASGEVRIS

>AtSnRK2.5(At5g63650)

MDKYEVVKDLGAGNFGVARLLRHKETKELVAMKYIERGRKIDENVAREIINHRSLRHPNIIRFKEVILTPTHLAIVMEYASGGELFERICNAGRFSEAEARYFFQQLICGVDYCHSLQICHRDLKLENTLLDGSPAPLLKICDFGYSKSSLLHSRPKSTVGTPAYIAPEVLSRREYDGKHADVWSCGVTLYVMLVGGYPFEDPDDPRNFRKTIQRIMAVQYKIPDYVHISQECRHLLSRIFVTNSAKRITLKEIKKHPWYLKNLPKELTEPAQAAYYKRETPSFSLQSVEDIMKIVGEARNPAPSSNAVKGFDDDEEDVEDEVEEEEEEEEEEEEEEEEEEDEYEKHVKEAHSCQEPPKA

>AtSnRK2.6(At4g33950)

MDRPAVSGPMDLPIMHDSDRYELVKDIGSGNFGVARLMRDKQSNELVAVKYIERGEKIDENVKREIINHRSLRHPNIVRFKEVILTPTHLAIVMEYASGGELFERICNAGRFSEDEARFFFQQLISGVSYCHAMQVCHRDLKLENTLLDGSPAPRLKICDFGYSKSSVLHSQPKSTVGTPAYIAPEVLLKKEYDGKVADVWSCGVTLYVMLVGAYPFEDPEEPKNFRKTIHRILNVQYAIPDYVHISPECRHLISRIFVADPAKRISIPEIRNHEWFLKNLPADLMNDNTMTTQFDESDQPGQSIEEIMQIIAEATVPPAGTQNLNHYLTGSLDIDDDMEEDLESDLDDLDIDSSGEIVYAM

>AtSnRK2.7(At4g40010)

MERYDILRDLGSGNFGVAKLVREKANGEFYAVKYIERGLKIDEHVQREIINHRDLKHPNIIRFKEVFVTPTHLAIVMEYAAGGELFERICNAGRFSEDEGRYYFKQLISGVSYCHAMQICHRDLKLENTLLDGSPSSHLKICDFGYSKSSVLHSQPKSTVGTPAYVAPEVLSRKEYNGKIADVWSCGVTLYVMLVGAYPFEDPEDPRNIRNTIQRILSVHYTIPDYVRISSECKHLLSRIFVADPDKRITVPEIEKHPWFLKGPLVVPPEEEKCDNGVEEEEEEEEKCRQSVEEIVKIIEEARKGVNGTDNNGGLGLIDGSIDLDDIDDADIYDDVDDDEERNGDFVCAL

>AtSnRK2.8(At1g78290)

MERYEIVKDIGSGNFGVAKLVRDKFSKELFAVKFIERGQKIDEHVQREIMNHRSLIHPNIIRFKEVLLTATHLALVMEYAAGGELFGRICSAGRFSEDEARFFFQQLISGVNYCHSLQICHRDLKLENTLLDGSEAPRVKICDFGYSKSGVLHSQPKTTVGTPAYIAPEVLSTKEYDGKIADVWSCGVTLYVMLVGAYPFEDPSDPKDFRKTIGRILKAQYAIPDYVRVSDECRHLLSRIFVANPEKRITIEEIKNHSWFLKNLPVEMYEGSLMMNGPSTQTVEEIVWIIEEARKPITVATGLAGAGGSGGSSNGAIGSSSMDLDDLDTDFDDIDTADLLSPL

>AtSnRK2.9(At2g23030)

MEKYEMVKDLGFGNFGLARLMRNKQTNELVAVKFIDRGYKIDENVAREIINHRALNHPNIVRFKEVVLTPTHLGIVMEYAAGGELFERISSVGRFSEAEARYFFQQLICGVHYLHALQICHRDLKLENTLLDGSPAPRLKICDFGYSKSSVLHSNPKSTVGTPAYIAPEVFCRSEYDGKSVDVWSCGVALYVMLVGAYPFEDPKDPRNFRKTVQKIMAVNYKIPGYVHISEDCRKLLSRIFVANPLHRSTLKEIKSHAWFLKNLPRELKEPAQAIYYQRNVNLINFSPQRVEEIMKIVGEARTIPNLSRPVESLGSDKKDDDEEEYLDANDEEWYDDYA

>AtSnRK2.10(At1g60940)

MDKYELVKDIGAGNFGVARLMRVKNSKELVAMKYIERGPKIDENVAREIINHRSLRHPNIIRFKEVVLTPTHIAIAMEYAAGGELFERICSAGRFSEDEARYFFQQLISGVSYCHAMQICHRDLKLENTLLDGSPAPRLKICDFGYSKSSLLHSMPKSTVGTPAYIAPEVLSRGEYDGKMADVWSCGVTLYVMLVGAYPFEDQEDPKNFKKTIQRIMAVKYKIPDYVHISQDCKHLLSRIFVTNSNKRITIGDIKKHPWFLKNLPRELTEIAQAAYFRKENPTFSLQSVEEIMKIVEEAKTPARVSRSIGAFGWGGGEDAEGKEEDAEEEVEEVEEEEDEEDEYDKTVKQVHASMGEVRVS

>AtSnRK3.1(At5g01810)

MEKKGSVLMLRYEVGKFLGQGTFAKVYHARHLKTGDSVAIKVIDKERILKVGMTEQIKREISAMRLLRHPNIVELHEVMATKSKIYFVMEHVKGGELFNKVSTGKLREDVARKYFQQLVRAVDFCHSRGVCHRDLKPENLLLDEHGNLKISDFGLSALSDSRRQDGLLHTTCGTPAYVAPEVISRNGYDGFKADVWSCGVILFVLLAGYLPFRDSNLMELYKKIGKAEVKFPNWLAPGAKRLLKRILDPNPNTRVSTEKIMKSSWFRKGLQEEVKESVEEETEVDAEAEGNASAEKEKKRCINLNAFEIISLSTGFDLSGLFEKGEEKEEMRFTSNREASEITEKLVEIGKDLKMKVRKKEHEWRVKMSAEATVVEAEVFEIAPSYHMVVLKKSGGDTAEYKRVMKESIRPALIDFVLAWH

>AtSnRK3.2(At5g07070)

MENKPSVLTERYEVGRLLGQGTFAKVYFGRSNHTNESVAIKMIDKDKVMRVGLSQQIKREISVMRIAKHPNVVELYEVMATKSRIYFVIEYCKGGELFNKVAKGKLKEDVAWKYFYQLISAVDFCHSRGVYHRDIKPENLLLDDNDNLKVSDFGLSALADCKRQDGLLHTTCGTPAYVAPEVINRKGYEGTKADIWSCGVVLFVLLAGYLPFHDTNLMEMYRKIGKADFKCPSWFAPEVKRLLCKMLDPNHETRITIAKIKESSWFRKGLHLKQKKMEKMEKQQVREATNPMEAGGSGQNENGENHEPPRLATLNAFDIIALSTGFGLAGLFGDVYDKRESRFASQKPASEIISKLVEVAKCLKLKIRKQGAGLFKLERVKEGKNGILTMDAEIFQVTPTFHLVEVKKCNGDTMEYQKLVEEDLRPALADIVWVWQGEKEKEEQLLQDEQGEQEPS

>AtSnRK3.3(At4g14580)

MESPYPKSPEKITGTVLLGKYELGRRLGSGSFAKVHVARSISTGELVAIKIIDKQKTIDSGMEPRIIREIEAMRRLHNHPNVLKIHEVMATKSKIYLVVEYAAGGELFTKLIRFGRLNESAARRYFQQLASALSFCHRDGIAHRDVKPQNLLLDKQGNLKVSDFGLSALPEHRSNNGLLHTACGTPAYTAPEVIAQRGYDGAKADAWSCGVFLFVLLAGYVPFDDANIVAMYRKIHKRDYRFPSWISKPARSIIYKLLDPNPETRMSIEAVMGTVWFQKSLEISEFQSSVFELDRFLEKEAKSSNAITAFDLISLSSGLDLSGLFERRKRKEKRFTARVSAERVVEKAGMIGEKLGFRVEKKEETKVVGLGKGRTAVVVEVVEFAEGLVVADVKVVVEGE

EEEEEVESHWSELIVELEEIVLSWHN

>AtSnRK3.4(At5g57630)

MGLFGTKKIGKYEIGRTIGEGNFAKVKLGYDTTNGTYVAVKIIDKALVIQKGLESQVKREIRTMKLLNHPNIVQIHEVIGTKTKICIVMEYVSGGQLSDRLGRQKMKESDARKLFQQLIDAVDYCHNRGVYHRDLKPQNLLLDSKGNLKVSDFGLSAVPKSGDMLSTACGSPCYIAPELIMNKGYSGAAVDVWSCGVILFELLAGYPPFDDHTLPVLYKKILRADYTFPPGFTGEQKRLIFNILDPNPLSRITLAEIIIKDSWFKIGYTPVYHQLSDSIKDNVAEINAATASSNFINAFQIIAMSSDLDLSGLFEENDDKRYKTRIGSKNTAQETIKKIEAAATYVSLSVERIKHFKVKIQPKEIRSRSSYDLLSAEVIEVTPTNCVIEISKSAGELRLYMEFCQSLSSLLTAEVS

>AtSnRK3.5(At5g45810)

MADLLRKVKSIKKKQDQSNHQALILGKYEMGRLLGHGTFAKVYLARNAQSGESVAIKVIDKEKVLKSGLIAHIKREISILRRVRHPNIVQLFEVMATKSKIYFVMEYVKGGELFNKVAKGRLKEEMARKYFQQLISAVSFCHFRGVYHRDLKPENLLLDENGNLKVSDFGLSAVSDQIRQDGLFHTFCGTPAYVAPEVLARKGYDGAKVDIWSCGVILFVLMAGFLPFHDRNVMAMYKKIYRGDFRCPRWFPVEINRLLIRMLETKPERRFTMPDIMETSWFKKGFKHIKFYVEDDHQLCNVADDDEIESIESVSGRSSTVSEPEDFESFDGRRRGGSMPRPASLNAFDLISFSPGFDLSGLFEDDGEGSRFVSGAPVGQIISKLEEIARIVSFTVRKKDCKVSLEGSREGSMKGPLSIAAEIFELTPALVVVEVKKKGGDKMEYDEFCNKELKPKLQNLSSENGQRVSGSRSLPSFLLSDTD

>AtSnRK3.6(At5g45820)

MDKNGIVLMRKYELGRLLGQGTFAKVYHARNIKTGESVAIKVIDKQKVAKVGLIDQIKREISVMRLVRHPHVVFLHEVMASKTKIYFAMEYVKGGELFDKVSKGKLKENIARKYFQQLIGAIDYCHSRGVYHRDLKPENLLLDENGDLKISDFGLSALRESKQQDGLLHTTCGTPAYVAPEVIGKKGYDGAKADVWSCGVVLYVLLAGFLPFHEQNLVEMYRKITKGEFKCPNWFPPEVKKLLSRILDPNPNSRIKIEKIMENSWFQKGFKKIETPKSPESHQIDSLISDVHAAFSVKPMSYNAFDLISSLSQGFDLSGLFEKEERSESKFTTKKDAKEIVSKFEEIATSSERFNLTKSDVGVKMEDKREGRKGHLAIDVEIFEVTNSFHMVEFKKSGGDTMEYKQFCDRELRPSLKDIVWKWQGNNNNSNNEKIEVIH

>AtSnRK3.7(At2g34180)

MAQVLSTPLAIPGPTPIQFMAGLLARIVTKNTNKETSTPESPRSPRTPQGSILMDKYEIGKLLGHGSFAKVYLARNIHSGEDVAIKVIDKEKIVKSGLAGHIKREISILRRVRHPYIVHLLEVMATKTKIYIVMEYVRGGELYNTVARGRLREGTARRYFQQLISSVAFCHSRGVYHRDLKLENLLLDDKGNVKVSDFGLSVVSEQLKQEGICQTFCGTPAYLAPEVLTRKGYEGAKADIWSCGVILFVLMAGYLPFDDKNILVMYTKIYKGQFKCPKWFSPELARLVTRMLDTNPDTRITIPEIMKHRWFKKGFKHVKFYIENDKLCREDDDNDDDDSSSLSSGRSSTASEGDAEFDIKRVDSMPRPASLNAFDILSFSDLSGLFEEGGQGARFVSAAP

MTKIISKLEEIAKEVKFMVRKKDWSVRLEGCREGAKGPLTIRVEIFELTPSLVVVEVKKKGGNIEEYEEFCNKELRPQLEKLMHYQADEVEEVMCLPPEIEQ

>AtSnRK3.8(At5g58380)

MENKPSVLTDKYDVGRLLGQGTFAKVYYGRSILTNQSVAIKMIDKEKVMKVGLIEQIKREISVMRIARHPNVVELYEVMATKTRIYFVMEYCKGGELFNKVAKGKLRDDVAWKYFYQLINAVDFCHSREVYHRDIKPENLLLDDNENLKVSDFGLSALADCKRQDGLLHTTCGTPAYVAPEVINRKGYDGTKADIWSCGVVLFVLLAGYLPFHDSNLMEMYRKIGKADFKAPSWFAPEVRRLLCKMLDPNPETRITIARIRESSWFRKGLHMKQKKMEKRVKEINSVEAGTAGTNENGAGPSENGAGPSENGDRVTEENHTDEPTNLNAFDLIALSAGFDLAGLFGDDNKRESRFTSQKPASVIISKLEEVAQRLKLSIRKREAGLFKLERLKEGRKGILSMDAEIFQVTPNFHLVEVKKSNGDTLEYQKLVAEDLRPALSDIVWVWQGEKDELTSQQETEYQQQQQQEQQEQEEPLKF

>AtSnRK3.9(At4g18700)

MAEKITRETSLPKERSSPQALILGRYEMGKLLGHGTFAKVYLARNVKTNESVAIKVIDKEKVLKGGLIAHIKREISILRRVRHPNIVQLFEVMATKAKIYFVMEYVRGGELFNKVAKGRLKEEVARKYFQQLISAVTFCHARGVYHRDLKPENLLLDENGNLKVSDFGLSAVSDQIRQDGLFHTFCGTPAYVAPEVLARKGYDAAKVDIWSCGVILFVLMAGYLPFHDRNVMAMYKKIYRGEFRCPRWFSTELTRLLSKLLETNPEKRFTFPEIMENSWFKKGFKHIKFYVEDDKLCNVVDDDELESDSVESDRDSAASESEIEYLEPRRRVGGLPRPASLNAFDIISFSQGFDLSGLFDDDGEGSRFVSGAPVSKIISKLEEIAKVVSFTVRKKDCRVSLEGSRQGVKGPLTIAAEIFELTPSLVVVEVKKKGGDKTEYEDFCNNELKPKLQNLTADDVVAEPVAVSAVDETAIPNSPTISFLPSDTE

>AtSnRK3.10(At3g23000)

MESLPQPQNQSSPATTPAKILLGKYELGRRLGSGSFAKVHLARSIESDELVAVKIIEKKKTIESGMEPRIIREIDAMRRLRHHPNILKIHEVMATKSKIYLVMELASGGELFSKVLRRGRLPESTARRYFQQLASALRFSHQDGVAHRDVKPQNLLLDEQGNLKVSDFGLSALPEHLQNGLLHTACGTPAYTAPEVISRRGYDGAKADAWSCGVILFVLLVGDVPFDDSNIAAMYRKIHRRDYRFPSWISKQAKSIIYQMLDPNPVTRMSIETVMKTNWFKKSLETSEFHRNVFDSEVEMKSSVNSITAFDLISLSSGLDLSGLFEAKKKKERRFTAKVSGVEVEEKAKMIGEKLGYVVKKKMMKKEGEVKVVGLGRGRTVIVVEAVELTVDVVVVEVKVVEGEEDDSRWSDLITELEDIVLSWHNDIM

>AtSnRK3.11(At5g35410)

MTKKMRRVGKYEVGRTIGEGTFAKVKFARNTDTGDNVAIKIMAKSTILKNRMVDQIKREISIMKIVRHPNIVRLYEVLASPSKIYIVLEFVTGGELFDRIVHKGRLEESESRKYFQQLVDAVAHCHCKGVYHRDLKPENLLLDTNGNLKVSDFGLSALPQEGVELLRTTCGTPNYVAPEVLSGQGYDGSAADIWSCGVILFVILAGYLPFSETDLPGLYRKINAAEFSCPPWFSAEVKFLIHRILDPNPKTRIQIQGIKKDPWFRLNYVPIRAREEEEVNLDDIRAVFDGIEGSYVAENVERNDEGPLMMNAFEMITLSQGLNLSALFDRRQDFVKRQTRFVSRREPSEIIANIEAVANSMGFKSHTRNFKTRLEGLSSIKAGQLAVVIEIYEVAPSLFMVDVRKAAGETLEYHKFYKKLCSKLENIIWRATEGIPKSEILRTITF

>AtSnRK3.12((At1g01140)

MSGSRRKATPASRTRVGNYEMGRTLGEGSFAKVKYAKNTVTGDQAAIKILDREKVFRHKMVEQLKREISTMKLIKHPNVVEIIEVMASKTKIYIVLELVNGGELFDKIAQQGRLKEDEARRYFQQLINAVDYCHSRGVYHRDLKPENLILDANGVLKVSDFGLSAFSRQVREDGLLHTACGTPNYVAPEVLSDKGYDGAAADVWSCGVILFVLMAGYLPFDEPNLMTLYKRICKAEFSCPPWFSQGAKRVIKRILEPNPITRISIAELLEDEWFKKGYKPPSFDQDDEDITIDDVDAAFSNSKECLVTEKKEKPVSMNAFELISSSSEFSLENLFEKQAQLVKKETRFTSQRSASEIMSKMEETAKPLGFNVRKDNYKIKMKGDKSGRKGQLSVATEVFEVAPSLHVVELRKTGGDTLEFHKVCDSFYKNFSSGLKDVVWNTDAAAEEQKQ

>AtSnRK3.13(At4g24400)

MVVRKVGKYELGRTIGEGTFAKVKFAQNTETGESVAMKIVDRSTIIKRKMVDQIKREISIMKLVRHPCVVRLYEVLASRTKIYIILEYITGGELFDKIVRNGRLSESEARKYFHQLIDGVDYCHSKGVYHRDLKPENLLLDSQGNLKISDFGLSALPEQGVTILKTTCGTPNYVAPEVLSHKGYNGAVADIWSCGVILYVLMAGYLPFDEMDLPTLYSKIDKAEFSCPSYFALGAKSLINRILDPNPETRITIAEIRKDEWFLKDYTPVQLIDYEHVNLDDVYAAFDDPEEQTYAQDGTRDTGPLTLNAFDLIILSQGLNLATLFDRGKDSMKHQTRFISHKPANVVLSSMEVVSQSMGFKTHIRNYKMRVEGLSANKTSHFSVILEVFKVAPSILMVDIQNAAGDAEEYLKFYKTFCSKLDDIIWKPPDASMRNRVTKAKSKRR

> AtSnRK3.14(At4g30960)

MVGAKPVENGSDGGSSTGLLHGRYELGRLLGHGTFAKVYHARNIQTGKSVAMKVVGKEKVVKVGMVDQIKREISVMRMVKHPNIVELHEVMASKSKIYFAMELVRGGELFAKVAKGRLREDVARVYFQQLISAVDFCHSRGVYHRDLKPENLLLDEEGNLKVTDFGLSAFTEHLKQDGLLHTTCGTPAYVAPEVILKKGYDGAKADLWSCGVILFVLLAGYLPFQDDNLVNMYRKIYRGDFKCPGWLSSDARRLVTKLLDPNPNTRITIEKVMDSPWFKKQATRSRNEPVAATITTTEEDVDFLVHKSKEETETLNAFHIIALSEGFDLSPLFEEKKKEEKREMRFATSRPASSVISSLEEAARVGNKFDVRKSESRVRIEGKQNGRKGKLAVEAEIFAVAPSFVVVEVKKDHGDTLEYNNFCSTALRPALKDIFWTSTPA

>AtSnRK3.15(At5g01820)

MVDSDPVEFPPENRRGQLFGKYEVGKLVGCGAFAKVYHGRSTATGQSVAIKVVSKQRLQKGGLNGNIQREIAIMHRLRHPSIVRLFEVLATKSKIFFVMEFAKGGELFAKVSKGRFCEDLSRRYFQQLISAVGYCHSRGIFHRDLKPENLLLDEKLDLKISDFGLSALTDQIRPDGLLHTLCGTPAYVAPEVLAKKGYDGAKIDIWSCGIILFVLNAGYLPFNDHNLMVMYRKIYKGEFRIPKWTSPDLRRLLTRLLDTNPQTRITIEEIIHDPWFKQGYDDRMSKFHLEDSDMKLPADETDSEMGARRMNAFDIISGSPGFNLSGLFGDARKYDRVERFVSAWTAERVVERLEEIVSAENLTVAKKETWGMKIEGQKGNFAMVVEINQLTDELVMIEVRKRQRAAASGRDLWTDTLRPFFVELVHESDQTDPEPTQVHTTS

>AtSnRK3.16(At3g17510)

MVRRQEEEKKAEKGMRLGKYELGRTLGEGNFGKVKFAKDTVSGHSFAVKIIDKSRIADLNFSLQIKREIRTLKMLKHPHIVRLHEVLASKTKINMVMELVTGGELFDRIVSNGKLTETDGRKMFQQLIDGISYCHSKGVFHRDLKLENVLLDAKGHIKITDFGLSALPQHFRDDGLLHTTCGSPNYVAPEVLANRGYDGAASDIWSCGVILYVILTGCLPFDDRNLAVLYQKICKGDPPIPRWLSPGARTMIKRMLDPNPVTRITVVGIKASEWFKLEYIPSIPDDDDEEEVDTDDDAFSIQELGSEEGKGSDSPTIINAFQLIGMSSFLDLSGFFEQENVSERRIRFTSNSSAKDLLEKIETAVTEMGFSVQKKHAKLRVKQEERNQKGQVGLSVTAEVFEIKPSLNVVELRKSYGDSCLYRQLYERLLKDVGTSSPEQEIVT

> AtSnRK3.17(At2g26980)

MLIPNKKLREMNRRQQVKRRVGKYEVGRTIGEGTFAKVKFARNSETGEPVALKILDKEKVLKHKMAEQIRREIATMKLIKHPNVVQLYEVMASKTKIFIILEYVTGGELFDKIVNDGRMKEDEARRYFQQLIHAVDYCHSRGVYHRDLKPENLLLDSYGNLKISDFGLSALSQQVRDDGLLHTSCGTPNYVAPEVLNDRGYDGATADMWSCGVVLYVLLAGYLPFDDSNLMNLYKKISSGEFNCPPWLSLGAMKLITRILDPNPMTRVTPQEVFEDEWFKKDYKPPVFEERDDSNMDDIDAVFKDSEEHLVTEKREEQPAAINAFEIISMSRGLNLENLFDPEQEFKRETRITLRGGANEIIEKIEEAAKPLGFDVQKKNYKMRLENVKAGRKGNLNVATEIFQVAPSLHMVQVSKSKGDTLEFHKFYKKLSNSLEQVVWTNNEVKKETAK

>AtSnRK3.18(At2g25090)

MEESNRSSTVLFDKYNIGRLLGTGNFAKVYHGTEISTGDDVAIKVIKKDHVFKRRGMMEQIEREIAVMRLLRHPNVVELREVMATKKKIFFVMEYVNGGELFEMIDRDGKLPEDLARKYFQQLISAVDFCHSRGVFHRDIKPENLLLDGEGDLKVTDFGLSALMMPEGLGGRRGSSDDLLHTRCGTPAYVAPEVLRNKGYDGAMADIWSCGIVLYALLAGFLPFIDENVMTLYTKIFKAECEFPPWFSLESKELLSRLLVPDPEQRISMSEIKMIPWFRKNFTPSVAFSIDETIPSPPEPPTKKKKKDLNEKEDDGASPRSFNAFQFITSMSSGFDLSNLFEIKRKPKRMFTSKFPAKSVKERLETAAREMDMRVKHVKDCKMKLQRRTEGRKGRLSVTAEVFEVAPEVSVVEFCKTSGDTLEYYLFCEDDVRPALKDIVWSWQGDDDEDDVTTNDNVDTNDNKINNVS

>AtSnRK3.19(At2g38490)

MAEDSNSSESIIVNVTGDDNKSALFGKYDLGKLLGSGAFAKVYQAEDLQNGGESVAIKVVQKKRLKDGLTAHVKREISVMRRLRHPHIVLLSEVLATKTKIYFVMELAKGGELFSRVTSNRFTESLSRKYFRQLISAVRYCHARGVFHRDLKPENLLLDENRDLKVSDFGLSAMKEQIHPDGMLHTLCGTPAYVAPELLLKKGYDGSKADIWSCGVVLFLLNAGYLPFRDPNIMGLYRKIHKAQYKLPDWTSSDLRKLLRRLLEPNPELRITVEEILKDPWFNHGVDPSEIIGIQADDYDLEENGKILNAFDLISSASSSNLSGLFGNFVTPDHCDQFVSDESTAVIMRKVEEVAKQLNLRIAKKKERAIKLEGPHGVANVVVKVRRLTNELVMVEMKNKQRDVGLVWADALRQKLRRLINQPVYKVPDKP

>AtSnRK3.20(At1g29230)

MAQALAQPPLVVTTVVPDPPPPPPPPHPKPYALRYMADLLGRIGIMDTDKDGNISPQSPRSPRSPRNNILMGKYELGKLLGHGTFAKVYLAQNIKSGDKVAIKVIDKEKIMKSGLVAHIKREISILRRVRHPYIVHLFEVMATKSKIYFVMEYVGGGELFNTVAKGRLPEETARRYFQQLISSVSFCHGRGVYHRDLKPENLLLDNKGNLKVSDFGLSAVAEQLRQDGLCHTFCGTPAYIAPEVLTRKGYDAAKADVWSCGVILFVLMAGHIPFYDKNIMVMYKKIYKGEFRCPRWFSSDLVRLLTRLLDTNPDTRITIPEIMKNRWFKKGFKHVKFYIEDDKLCREDEDEEEEASSSGRSSTVSESDAEFDVKRMGIGSMPRPSSLNAFDIISFSSGFDLSGLFEEEGGEGTRFVSGAPVSKIISKLEEIAKIVSFTVRKKEWSLRLEGCREGAKGPLTIAAEIFELTPSLVVVEVKKKGGDREEYEEFCNKELRPELEKLIHEEVVVEEALYLPSDTE

>AtSnRK3.21(At1g48260)

MVIKGMRVGKYELGRTLGEGNSAKVKFAIDTLTGESFAIKIIEKSCITRLNVSFQIKREIRTLKVLKHPNIVRLHEVLASKTKIYMVLECVTGGDLFDRIVSKGKLSETQGRKMFQQLIDGVSYCHNKGVFHRDLKLENVLLDAKGHIKITDFGLSALSQHYREDGLLHTTCGSPNYVAPEVLANEGYDGAASDIWSCGVILYVILTGCLPFDDANLAVICRKIFKGDPPIPRWISLGAKTMIKRMLDPNPVTRVTIAGIKAHDWFKHDYTPSNYDDDDDVYLIQEDVFMMKEYEEEKSPDSPTIINAFQLIGMSSFLDLSGFFETEKLSERQIRFTSNSLAKDLLENIETIFTEMGFCLQKKHAKLKAIKEESTQKRQCGLSVTAEVFEISPSLNVVELRKSHGDSSLYKQLYERLLNELGSSSQVQELLA

>AtSnRK3.22(At2g30360)

MPEIEIAAGSGDNNDALFGKYELGKLLGCGAFAKVFHARDRRTGQSVAVKILNKKKLLTNPALANNIKREISIMRRLSHPNIVKLHEVMATKSKIFFAMEFVKGGELFNKISKHGRLSEDLSRRYFQQLISAVGYCHARGVYHRDLKPENLLIDENGNLKVSDFGLSALTDQIRPDGLLHTLCGTPAYVAPEILSKKGYEGAKVDVWSCGIVLFVLVAGYLPFNDPNVMNMYKKIYKGEYRFPRWMSPDLKRFVSRLLDINPETRITIDEILKDPWFVRGGFKQIKFHDDEIEDQKVESSLEAVKSLNAFDLISYSSGLDLSGLFAGCSNSSGESERFLSEKSPEMLAEEVEGFAREENLRMKKKKEEEYGFEMEGQNGKFGIGICISRLNDLLVVVEARRRGGDGDCYKEMWNGKLRVQLIRVCDQTSSTNAAI

>AtSnRK3.23(At1g30270)

MASRTTPSRSTPSRSTPSGSSSGGRTRVGKYELGRTLGEGTFAKVKFARNVENGDNVAIKVIDKEKVLKNKMIAQIKREISTMKLIKHPNVIRMFEVMASKTKIYFVLEFVTGGELFDKISSNGRLKEDEARKYFQQLINAVDYCHSRGVYHRDLKPENLLLDANGALKVSDFGLSALPQQVREDGLLHTTCGTPNYVAPEVINNKGYDGAKADLWSCGVILFVLMAGYLPFEDSNLTSLYKKIFKAEFTCPPWFSASAKKLIKRILDPNPATRITFAEVIENEWFKKGYKAPKFENADVSLDDVDAIFDDSGESKNLVVERREEGLKTPVTMNAFELISTSQGLNLGSLFEKQMGLVKRKTRFTSKSSANEIVTKIEAAAAPMGFDVKTNNYKMKLTGEKSGRKGQLAVATEVFQVAPSLYMVEMRKSGGDTLEFHKFYKNLTTGLKDIVWKTIDEEKEEGTDGGGTNGAMANRTIAKQST

>AtSnRK3.24(At5g10930)

MEEERRVLFGKYEMGRLLGKGTFAKVYYGKEIIGGECVAIKVINKDQVMKRPGMMEQIKREISIMKLVRHPNIVELKEVMATKTKIFFVMEFVKGGELFCKISKGKLHEDAARRYFQQLISAVDYCHSRGVSHRDLKPENLLLDENGDLKISDFGLSALPEQILQDGLLHTQCGTPAYVAPEVLKKKGYDGAKADIWSCGVVLYVLLAGCLPFQDENLMNMYRKIFRADFEFPPWFSPEARRLISKLLVVDPDRRISIPAIMRTPWLRKNFTPPLAFKIDEPICSQSSKNNEEEEEDGDCENQTEPISPKFFNAFEFISSMSSGFDLSSLFESKRKVQSVFTSRSSATEVMEKIETVTKEMNMKVKRTKDFKVKMEGKTEGRKGRLSMTAEVFEVAPEIS

VVEFCKSAGDTLEYDRLYEEEVRPALNDIVWSWHGDNNNTSSEDC

>AtSnRK3.25(At5g25110)

MGSKLKLYPLLNHSSVFHPDSRYQSAPTMEEEQQQLRVLFAKYEMGRLLGKGTFGKVYYGKEITTGESVAIKIINKDQVKREGMMEQIKREISIMRLVRHPNIVELKEVMATKTKIFFIMEYVKGGELFSKIVKGKLKEDSARKYFQQLISAVDFCHSRGVSHRDLKPENLLVDENGDLKVSDFGLSALPEQILQDGLLHTQCGTPAYVAPEVLRKKGYDGAKGDIWSCGIILYVLLAGFLPFQDENLMKMYRKIFKSEFEYPPWFSPESKRLISKLLVVDPNKRISIPAIMRTPWFRKNINSPIEFKIDELEIQNVEDETPTTTATTATTTTTPVSPKFFNAFEFISSMSSGFDLSSLFESKRKLRSMFTSRWSASEIMGKLEGIGKEMNMKVKRTKDFKVKLFGKTEGRKGQIAVTAEVFEVAPEVAVVELCKSAGDTLEYNRLYEEHVRPALEEIVWSWHGDNHNNNIVKSNGNYVSDENSGSDC

>AtSnRK3.26 (At5g21326)

MNRPKVQRRVGKYEVGKTLGQGTFAKVRCAVNTETGERVALKILDKEKVLKHKMAEQIRREICTMKLINHPNVVRLYEVLASKTKIYIVLEFGTGGELFDKIVHDGRLKEENARKYFQQLINAVDYCHSRGVYHRDLKPENLLLDAQGNLKVSDFGLSALSRQVRGDGLLHTACGTPNYAAPEVLNDQGYDGATADLWSCGVILFVLLAGYLPFEDSNLMTLYKKIIAGEYHCPPWLSPGAKNLIVRILDPNPMTRITIPEVLGDAWFKKNYKPAVFEEKEEANLDDVDAVFKDSEEHHVTEKKEEQPTSMNAFELISMSRALDLGNLFEEEEGFKRETRFAAKGAANDLVQKIEEASKPLGFDIQKKNYKMRLENVTAGRKGNLRVATEIFQVSPSLHMIEVRKTKGDTLEFHKFYKKLSTSLNDVVWKSGESSGLSK
