# Supplementary material for: Genome-Wide Characterization of Snf1-Related Protein Kinases (SnRKs) and Expression Analysis of SnRK1.1 in Strawberry
Source: Genes (Basel). 2020 Apr 16;11(4):427. doi: 10.3390/genes11040427 (PMC7230852; doi:10.3390/genes11040427)
Supplement: Supplementary file 1 [file genes-11-00427-s001.zip › Supplementary File S2.docx]

Supplementary File S2: Protein sequences of strawberry SnRK genes.

>FvSnRK1.1

MDGAIGRGGSSADAYLPNYKLGKTLGIGSFGKVKIAEHALTGHKVAIKILNRRKIKNMEMEEKVRREIKILRLFMHPHIIRLYEVIETPSDIYVVMEYVKSGELFDYIVEKGRLQEDEARNFFQQIISGVEYCHRNMVVHRDLKPENLLLDSKCNVKIADFGLSNIMRDGHFLKTSCGSPNYAAPEVISGKLYAGPEVDVWSCGVILYALLCGTLPFDDENIPNLFKKIKGGIYTLPSHLSPGARDLIPRMLVVDPMKRMTIPEIRQHKWFQAHLPRYLAVPPPDTMQQAKKIDEEILQEVVKMGFDRNHLVESLRGRLQNEGTVAYYLLLDNRFRVSSGYLGAEFQETVESGFNRMHQGEPASSPVGHRLPGYMEFQGMGSSPFRQQFPVERKWALGLQSRAHPREIMTEVLKALQELRVCWKKIGHYNMKCRWVPGTPGHHEGMVDNPVNNNHYFGDESSIIENDGIMKTPNVVKFEVQLFKTREEKYLLDLQRVQGPQFLFLDLCAAFLAQLRVL

>FvSnRK2.1 MEKYELVKDIGSGNFGVARLMRNKETKELVAMKYIDRGLKIDENVAREIINHRSLRHPNIIRFREVVLTPTHLAIVMEYAAGGELFERICNAGRFSEDEARYFFQQLISGVSYCHSLQICHRDLKLENTLLDGSPAPRLKICDFGYSKSSLLHSRPKSTVGTPAYIAPEVLSRREYDGKLADVWSCGVTLYVMLVGAYPFEDQEDPKNFRKTINRIMAVQYKIPDYVHISQDCRHLLSRIFVANPARRITIKEIKNHPWFLKNLPRELTEAAQTMYYRKENPTFSLQSVEDIMKIVEEAKNPPPVSRSVGGFGWGGEEDGDAKDEVEEGEDEEDEYEKRVKEAHQSGEVRVV

>FvSnRK2.2 MEERYEPMKDLGSGNFGVARLVRDKKTRELVAVKYIERGKKIDENVQREIINHRSLRHPNIVRFKEVLLTPSHLAIVMEYAAGGELFERICSAGRFSEDEARFFFQQLISGVSYCHSMEICHRDLKLENTLLDGSATPRLKICDFGYSKSAILHSQPKSTVGTPAYIAPEVLSRKEYDGKIADVWSCGVTLYVMLVGAYPFEDPEDPRNFRKTIERIMSVQYTIPDYVRISADCKHLLSRIFVANPSKRLSLPEIKQHPWFLKNLAKELIEVEKKSFAEVERDNPTQNIEEINKIIQDARTPGEGSKGVGQAVAGAGPSDSDDVDLDSEVDLSGDFA

>FvSnRK2.3 MDRSMLTVGPGMDMPIMHDSDRYELVKDIGSGNFGVARLMRDKQTEELVAVKYIERGEKIDENVQREIINHRSLRHPNIVRFKEVILTPTHLAIVMEYASGGELFERICNAGRFSEDEARFFFQQLISGVSYCHAMQVCHRDLKLENTLLDGSPAPRLKICDFGYSKSSVLHSQPKSTVGTPAYIAPEVLLKKEYDGKIADVWSCGVTLYVMLVGAYPFEDPGEPKNFRKTIHRITSVQYSIPDYVHISPECRHLISRIFVAEPEKRITIPEIRNHEWFLRNLPADLMVENTMNSQFEEPDQPMQSIDEIMQIIAEATIPAAGTNNLNQYLAGSLDIDNMEEDLDTDPDDLDIDSSGEIVYAI

>FvSnRK2.4 MEKYEFVEDIGSGHFGVAKLMRNKETKELVAVKYIDRGLKIDQNVAREIINHRSLCHPNIIQFREVFLTPTHLAIVMEYAAGGELFDRVANAGRLSEDEARFFFRQFISGVSHCHSMQICHRDLKLDNTLLDRTSSSPRLKICDFGFSKSSLLHSRTKSIVGSPAYTAPEVFSRKEYDGKLADVWSCGVSLYVMVVGAYPFADENDQTNIREIVKRIMSVRYVIPGYVYISEDCRHLLSRIFVADPARRITIQEIKNHPWFLKNLPRELTEEAQTRKENSTCSLQSVEDIMKIVEEAKNIPPPVSRSVGGSGDAEKTSMKRESKKHIKVGKEIEEGDLKKTSMKRESKKHIKVGRYGVCRMSST

>FvSnRK2.5 MERYEIVKDIGSGNFGVAKLVKDKWSGELYAIKFIERGQKIDEHVQREIMNHRSLKHPNIIRFKEVLLTQTDLAIVMEYAAGGELFERICNAGRFSEDEARFFFQQLISGVSYCHSMQICHRDLKLENTLLDSSSAPRLKICDFGYSKSSVLHSQPKSTVGTPAYIAPEVLSKKEYDGKIADVWSCGVTLYVMLVGAYPFEDPEDPRNFRKTLQRILSVSYSIPDYVRVSRECTHLLSRIFVANPEKRITIPEIKQHPWFLKNLPSEFMDEDDMQIGEVQKNEISQSVEDIVSIIQEARKLGDGIKVGHFLGSMDLDEIDDADIDDIETSGDFVCAL

>FvSnRK2.6 MERYEIVKDIGSGNFGVARLVRDKLTRELFAVKFIERGQKIDEHVQREIMNHRSLKHSNIVQFKEVLLTPTHLAIVMEYAAGGELFGRICNAGRFSENEARFFFQQLISGVSYCHSMQICHRDLKLENTLLDGSTAPRVKICDFGYSKSLLQSQPKSTVGTPAYIAPEVLSKKQYDGKIADVWSCGVTLFVMIAGAYPFEDPDDPKNFRKTINRILAVHYSIPDHVQVSIECRHLLSQIFVENPEKRITIAGIKSHPWFLKNLPMELMEGGSWQSNDVNIPSQSTEEVLSIIQEARKSVPYPNTVTHLIEGNMDLDDLDAEVEDVETSGDFVCHLSS

>FvSnRK2.7 MEKYEVVKDIGSGNFGVARLVRDKSTNELLAVKFIERGIKIDENVQREIMNHRSLKHPNIVQFKEILLTPTHLAIVMEYAAGGELYDRIVKATRFSENEARFFFQQLISGVSYCHTMQICHRDLKLENTLLDDSAAPRVKICDFGYSKSLLSSLPKSTVGTPAYIAPEVLSKKQYNGQVADVWSCGVTLYVMLVGAYPFEDPKDPMNFRKTIRRILTVRYAIPDSVRVSVECRHLLSKIFVANPEKRITIPDLKMHRWFAKNLPLEMKEGGSWENNAVNTPSQSIEEVQSIIKEARKPLKVPTVSRHCVGSSMAIDDAEE

>FvSnRK2.8 MRQEPSLVQNVQQWSCDTMISSGTPTAPPDMRYEILKDIGAGNFAVTKLVREKCSGELYAVKLIERGKKIDENVQREIMNHRSLKHPNIVEFKEVLLTPTHLGIVMEYAAGGELYERICKAGRFSEDEARFFFQQLISGLKYCHTMHICHRDLKLENSLLDGGTTPRVKICDFGYSKSLLQSQPKSVVGTPNYIAPEVLSRKKYDGKISDVWSCGVTLFVMIVGAYPFEDPQDPINFTKTIRNVAVSRECRHLFSKIFVANPEKRITIPEIKSHPWFVKNLPMEMMEGGSWESNEMNNPVQSVEEVQSIIQEARRPLKVRTVSRHLTGSSMALDEADVGGPETSGDFVC

>FvSnRK2.9 MDRAAVTVGPAMDMPIMHDSDRYDFVRDIGSGNFGVARLMTDKQTKELVAVKYIERGNKIDENVQREIINHRSLRHPNIVRFKEVILTPTHLAIVMEYASGGELFERICNSGRFSEDEARFFFQQLISGVSYCHAMQVCHRDLKLENTLLDGSPAPRLKICDFGYSKSSVLHSQPKSTVGTPAYIAPEVLLRQEYDGKVLSIWNPVLGHIHVVLVLTFSMKQQLSELKTVISSDRQNLFTTADVWSCGVTLYVMLMGSYPFEDPDEPKDFRKTIQRILNVQYAIPDSVPLSCECLELISRIFVSDPTARITIPEIKNHPWFLKNLPADLMDEMTMGNHFEEPDQPMQSIDTIMQIIAEATIPAVGIHNLSPFMNDSFDMDDDMDDLDSESELDVDSSGEIVYAI

>FvSnRK3.1 MVLKLEMDNKSYILMQRYELGRQLGKGTFAKVYYARSLITNQAVAIKVVDKEKIMKVGLMDQIKREISVMRLVRHPNIIHLYEVLATKTKIYFVIEYAKGGELFNKVAKGKLKEDVARKYFQQLINALDFCHSRGVYHRDIKPENLLLDENDNLKISDFGLSALAESKRQDGLLHTTCGTPAYVAPEVINRKGYDGVKADVWSCGVVLYVLLAGYLPFHDSNLMEMYRKIGKAEFRCPNWFSPEARRLLCKMLDPNPNTRITLAKVRESSWFRRGPKSKEKEVAPAATEASSPSENENNLAVEANQESGRPSNLNAFDIISLSDGFDLSGLFEKNSLSREARFTSRKPATVIISKLEEMAKYLKLKVNKKDHGLLKMDRLQEGRKGFLSIDAEIFEITPNFHLVEVKKSNGDTMEYQQMLEDIRPALRDIVWVWQGEQEQTSQVQQLQEQEEVQQQQQEQLPQNPPQL

>FvSnRK3.2 MENKGSVLMQRYELGRLLGQGTFAKVYHARNLKTNMSVAIKIIDKERILKVGMIDQIKREISVMRLIRHPNVVELYEVMASKTKIYFVMEYVKGGELFDKVSKGKLKEDVARKYFQQLVSAVDYCHSRGVYHRDLKPENLLLDENGNLKVSDFGLSALGESKRQDGLLHTTCGTPAYVAPEVINRKGYDGAKADIWSCGVVLFVLLAGYLPFHDANLMELYRKIGKGEFKFPNWFTPEVRRLLSKIFDPNPNTRISLSKVMQSSWFRKGLVQKPAIVELPVKELAPLDADSIFGPGEDNNSVTEAKQELVQKPSNLNAFDIISYSAGFDLSGLFEEAEQKKEVRFTSNKTASTIISKLEDIAKRLKLKIKKKDGGLLRMEGSTEGRKGVLGIETEIFEITPSFHLVEVKKSSGDTLEYQKVMKKEVRPGLKDIIWTWQGEQQPQQQEPPQPEQQEQQPLTLPVQVASPQEA

>FvSnRK3.3 MAELRPQNGAVSTPTTTFTSNTSKTKNNPLLLGRFEIGKLLGHGTFAKVYHARNIKTDQGVAIKVIDKEKILKGGLIAHIKREISILRRVRHPNIVQLFEVMATKAKIYFVMEYVRGGELFNKVAKGRLKEEVARKYFQQLISAVGFCHARGVYHRDLKPENLLLDENGDLKVSDFGLSAVSDEIRQDGLFHTFCGTPAYVAPEVLGRKGYDAAKVDIWSCGIVLFVLMAGYLPFHDHNVMAMYKKIYKGEFRCPRWFSSELVKLLTRLLDTNPNTRITIAEVMENRWFKKGFKHIKFYIDHDDRLCNVHEDDGDDSDASSVMSDMSESEAEFETRRKLTTLPRPASLNAFDIISFSPGFDLSGLFEERGEEARFVSGAPVDKIISKLEEIAKVVSFSVRKKDCRVSLEGSREGVKGPLTIAAEIFELTPSLVVLEVKKKAGDKVEYDQFCNTELRPGLQNLMIEESAGGSLASGGSVVSEGSLASGGSVVSVGSPSSVHHLPSDTE

>FvSnRK3.4 MNQPKIKRRVGKYEVGRTIGEGTFAKVKFARNSETGEPVALKILDKEKVLKHKMAEQIKREIATMKLIKHPNVVQLYEVMGSKTKIFIVMEFVTGGELFDKISFLYLDVLFLLDSSSSDYLVDLAYLLTKTYFALKVNNGRMREDEARRYFQQLINAVDYCHSRGVYHRDLKPENLLLDAYGNLKVSDFGLSALSQQVRDDGLLHTTCGTPNYVAPEVLNDRGYDGATADLWSCGVILFVLLAGYLPFDDSNLINLYRKISAGEFTCPPWLSFGAMKLIARILDPNPMTRITICEILEDEWFKKDYKSLMFEEKEDTNLDDVEAVFKDSEEHHVTEKKEEQPTAMNAFELISMSKGLNLGNLFDVEQGFKRETRFTSRCPANEIIHKIEEAAKPLGFDVQKKNYKLRLENMKAGRKGNLNVATEIFQVAPSLHMVEVRKAKGDTLEFHKFYKNLSTCLEDVVWKTEEDMLE

>FvSnRK3.5 MIGFRLRQYKKSYVGKLEPCDLDHRRMSAPKSPRMRTRVGKYELGKTLGEGTFAKVKFAKNTETGQCVAIKILDREQVLKHKMVEHIKREISTMKLIKHPNVTQMFEVMASKTKIYIVLEFVDGGELFDEIAKNGRLKEDNARRYFQQLINAVDYCHSRGVYHRDLKPENLLLDSFGVLKISDFGLSTFEQQVREDGLLHTACGTPNYVAPEVLNNKGYEGKSSDVWSCGVILFVLMAGYLPFDEPNLIALYRKICKAEFSCPAWFSSGAKKLIHRILDPNPATRMTIPEILENDWFKKDYKPAQFKEEDNINLDDVDAVFNNSKENFVTERREKPTSMNAFELISRSQSFNLENLFEKQMGLVKRETRFTSQRPANEIMSKIEETAKPLGFNIRKKDYKMKLQGDKHGRKGHLSVATEVFEVAPSVHMVELRKTGGDTLEFHKASHH

>FvSnRK3.6 MAARAAGVGSRTRVGRYDLGRTLGEGNFAKVKFARNVETGENFAIKILDKEKVLKHKMIGQVMASKTKIYIVLEFVTGGELFDKIASKGRLKEDEARKYFQQLINAVDYCHSRGVFHRDLKPENLLLDVNGVLKVSDFGLSALPQQVREDGLLHTTCGTPNYVAPEVINNKGYDGAKADLWSCGVILYVLMAGYLPFEDSNLMALYKKIFKAEFSCPPWFSSSAKKLIKRILDPNPLTRITFAEVIENEWFKKGYKPPSFEQVDVSLDDVDAIFNDPGDSQNFVVEKREERHVPVTMNAFELISTSQGLNLNSLFEKQMELVKRETRFTSKRPANEIISKIEEAAAPLGFGVKKNNFKLKLQGEKTGRKGHLSVATEIFEVAPSLYMVEVRKSGGDTLEFHNFYKNLSTGLKDIVWKSGDDARKEAEFGSASSSGTAAGAGAVSST

>FvSnRK3.7 MANEKSAGSALLHGKYELGRMLGHGTFAKVYHARNLKTGKSMAMKVVGKEKVIKVGMMEQIKREISVMRMVRHPNIVELHEVMASKSKIYFAMDLVRGGELFAKIAKGRLKEDVARVYFQQLISAVDFCHSRGVYHRDLKPENLLLDEDGDLKVTDFGLSAFSEHLKQDGLLHTTCGTPAYVAPEVIGKKGYDGAKADLWSCGVILYVLLAGFLPFQDDNLVSMYRKIYKGDFKCPPWFSSEARRLITKLLDPNPSTRISIAKIMDSSWFKKSIPKTVKSKKEREFDETTEKTSKQMETLNAFHIISLSEGFDLSPLFEEKKREEREELRFATTRSASSVISKLEEVGKAGKFKVKKSDSMVRLQGEASGRKGKLAIAAEIFAVTPSFLVVEVKKDNGDTLEYNQFCSKELRPALKDIVWTNSAPPA

>FvSnRK3.8 MENKKANILMHKYELGRLLGKGTFAKVYHARNLRTGQSVAIKIIDKEKVQQVGLIDQIKREISVMRLVRHPNVVQLYEVMASKTKIYFAMEYVKGGELFNKVAKGKLKEDIARKYFQQLIGAVDYCHSRGVYHRDIKPENLLVDEHGNLKVSDFGLSALIESRGQDGLLHTTCGTPAYVAPEVINKKGYDGAKADTWSCGVVLYVLLAGFLPFHDTNLMEMYRKISRGDFKSPQWFPPEVRKLLARILDPNATMRISVDKIMENSWFKKGFKHIDAPLPIPCDPSTSISDVHSAFGSPDSSEGSSNRKAETTNAASPMRPTNFNAFDIISLSPGFDLSGLFEGDHKHRSSQSRFTTTKPASTIVSKFEQIAQMERFRCMQKDGTVKLQGSREGRKGQLGIDAEIFEVTPSFFVVEVKKTAGDTLEYIQFYDHDLKPSLKDIVWTWQGNDPQQQHQPATQVS

>FvSnRK3.9 MPEIEVVSDAGDGASEASSLDETGGALFGKYELGKLLGRGAFAKVYHARDVSSGQSVAIKAVSKQKVLKGGFTSNVKREISIMRRLQHPHIVKLYEVLATKTKIYFIMEFAKGGELFGKISKGRFSEDLSRRYFQQLISAVGYCHSRGVYHRDLKPENLLLDENWNLKVSDFGLSAVTEQIRPDGLLHTLCGTPAYVAPEILAKKGYDGAKVDIWSCGIILFVLNAGYLPFNDPNLMVMYRKIYKGEFRFPRWTSPGLRRLISRLLDTNVETRITVDEIIKDPWFSVGYKDVKFHLEDFNLKEWRDEDNDTPLNAFDLISFSSGFDISGLFRKPEISDCGERFVSAETPERIIQKVEEVALAEGMTVMEKKTWGAKLAGQNGNLVVAIGIYRLTEKLVVVELNKRERIGENCQQIWKDKLRPQLSCLIYKPEEEQVSGE

>FvSnRK3.10 MMWMSYPQSRASQLRRSPETPRITSLRLQKVPLRTCLASPATGIFAFPASEVNERLGKTMMKKKVTRNVGKYEVGRTIGEGTFAKVKFARNAETGESVAMKVLAKSTILKHRMVDQIKREISIMKIVRHPNIVRLHEVLAGRTKIYIILEFVTGGELFDKIVHQGKLRENESRKYFQQLIDAVSHCHSKGVYHRDLKPENLLLDAYGNLKVSDFGLSALPQQGDGLLFTTCGTPNYVAPEVLGSKGYDGAAADVWSCGVILYVLMAGYLPFDEANLAALYKKINAAEFSCPFWFSPGANSLIHKILDPNPKTRFRIEEIRKDPWFRKNYAPVEYREDEEVSLDDVRAVFEDIEDQYAEERTDNKDSGPLLMNAFEMITLSQGLNLSALFDRRQDYIKRQTRFVSRKPAKVIISNVEAVAESMSLKVHTRNFKTRLEGISANKAGQFAVVLEIFEVAPSLFMVDVRKAAGDTLEYHKFYKNFCAKLDDIIWKPKDGMASSNILRTTTC

>FvSnRK3.11 MEERTVLFGKYETGRLLGKGTFAKVYYGRQIETNESVAIKVISKEQVKKEGMMEQIKREISATRLLRHPNIVQLKEVMATKTKIFIVMEYVKGGELFAKVAKGKLKEDQARKYFQQLISAVDFCHSRGVSHRDLKPENLLLDENGDLKISDFGLSSLPEQLRNDGLLHTQCGTPAYVAPEVLRKKGYDGSKTDIWSCGVILFVLLAGFLPFQDENIMKMYRKVFKAEFECPPWFSTEAKRLVSKLLVSDPERRITIPEIMRVPWFRKGYTRPLAFSPPPASSDKSFDEDFGSTAPAPADAANHKSQSPNFFNAFQFISSMSSGFDLSNLFESKRKAGTMFTSKCSSAAIMAKIEHAAKALSFKVGTVKDFKLRLQGPNEGRKGRLSVTAEVFEVAPEVAVVEFSKSAGDTLEYAKFCEEDVRPALKDIVWTWQGDGNKVDGEE

>FvSnRK3.12 MVIISKGQEKSSDQSKKGMRLGKYELGKTLGEGNFGKVKFAKDVGSGQPFAVKILEKKRITDLNIADQIKREIGTLKLLKHPNVVRLHEVVASKTKIYMVLEYVTGGELFDKIAQKGRLKESEGRKLFQQLIDGVSYCHNQGVFHRDLKLENILVDSKGNIKISDFGLSALPQHFREDGLLHTTCGSPNYVAPEILANRGYDGGTSDIWSCGVILYVILTGYLPFDDRNLAVLYQKILKGDVQIPKWLSPGAQNLIRRVLDPSPLTRINMTDIKSDEWFKQDYFPAKADEEEEDINVDTEAYSIKEVPSEGEKSPDLRHSPTLINAFQLIGMSSCLDLSGFFEKEDVSERKIRFTSNHSAKDLLERIEEIVIEMGYAVQKKNGRAKIMLLPSTFQLKVMQENKGQRNLGSLSVAAEVFELSPTLHVVELRKSYGDPSAYRQLCKKLSNELGVPSSQELLASEVLKSSSLQSQTA

>FvSnRK3.13 MGRGPPPPTPSSAAAAAATTTTNLLGKYQISRMLGRGSFAKVYKAQTIADETPVAIKIIDKLKTHAAMEPLILREISAMRRLQDHPNILKIHEVMATKSKIYIVVELATGGELFAKISRHGKLPESLARRYFQQLVSALRFCHENGVAHRDVKPQNLLLDGNGDLKVSDFGLSALPEQLKNGLLHTACGTPAYTAPEVLYRVGYDGSKADAWSCGVILFVLLAGHLPFDDSNLVAMHKKIQRRDYVIPAAISKPARRIIYQLLDPNPNTRLSVEAVMEKAWFQKAIDLKLVSDGCDVFELEKPPAKCDVVSGMNAFDIISMSSGLDLSGLFEAENRSERRFTANVAAEKVAEKVGEVGERMGYKAERGKGGMSVGLGKGGKGKGRRVALVVEMMEVAAGLVLGEVKMVEGGVEFPELLWEDLKTGLGDVVVSWQNGGV

>FvSnRK3.14 MPEIEQQQQQVVLRIPDNALFGKYELGKLLGCGAFAKVYHARNVFTGQSVAVKVINKKKLNGTSLMSNVTREISIMRRLRHPNIVKLYEVMASKTKIYFILEFVKGGELFAKVSKGRFSEALSRKYFQQLISAVGYCHSRGVYHRDLKPENLLVDDNGNLKVSDFGLSAVTGQIRPDGLLHTLCGTPAYVAPEILTKRGYDGAKVDMWSCGVILYVLNAGFLPFNDPNLMAMYKKIYKGEFRCPKWMSQDLKRFLGRVLDTNPMTRITVDGVLNDPWFRKGGEYKEISFYDDDKPDEEQKCMTNLNAFDIISYSAGLDLSGLFESTNPIQDSERIVSSETVETMVGRVEEFAKEEKLRVRRKKDWGMEMEGQNGNLVIGVEVSRLTESLVVVEAKRTGGESGPFNDMWNKLKPRLVLGREETVEEDLSLIASSSSLSSV

>FvSnRK3.15 MVVRKVGKYEIGRTIGEGTFAKVKFAQNTETGESVAMKIIDRSSIIKHKMVDQGVELEMLMFMMQIKREISIMKLVLASRTKIYIILEFITGGELFDKIVHHGRLSEGEARRFFQQLIDGVDYCHSKGVYHRDLKPENLLLDSIGNLRISDFGLSALPEPGVSLLRTTCGTPNYVAPEVLSHKGYDGAVADVWSCGVILYVLMAGYLPFDELDLTTLYKDDSSDEGHNDICKFDGGVFAASKEDDKLHSKRMCFFQRITIQQIRNDEWFQKNYVPPKILEYEDVNLDDVNAVFDETEEERDSEHQGSEDMGPLVLNAFDLIILSQGLNLASMFDRGKDCMKYQTRFVSQKPAKVVLSSMEVVAQSMGFKTHIRNYKMRVEGLSANKTSHFSIILEIFEVAPTYYMVDIQKAAGDASEFLKCTALSLVSDSLLFYSFTRTFVAILRISSGNRLLNLANQGSARVKVESVDILHSCFHSTVIAGLVLDNAFGGMGH

>FvSnRK3.16 MDKEGEVLLGKYQLGKLLGQGGFAKVYHARSLKTNQIVAIKIISKEKVFELGLVDQTKREISIMRLLKHPNIVQLYEVMATKKKIYLVMEYAEGGELFQKINKRRLKEEAARRFFQQLITAVDFCHKRGVFHRDLKPENLLLDKDGVLKVSDFGLSAFSESKRKHALLHTTCGTPNYVAPEVIRLGAYDGAKADIWSCGVILFQLLAGYRPFDDSNLNNMFRKICASEYRCPRWFSDDIRKLLFGILNTNPNERFLASDIMRSSWFQEGLSSKIKTEVEDVDGESDDCDKSENQETITPATLSAFDIISLSSGFDLSGLMMQKDAKRSAVQFTSAQSATSIMTKLRDITRKLKLKSKKEGASLKLMKGALSIEAEIFEFTPSFHLVEMKKSNGDTFEFRKMVDEDIRPALKDVVWTWQGERSNNNSSICV
