# Supplementary material for: Genome-Wide Characterization of Snf1-Related Protein Kinases (SnRKs) and Expression Analysis of SnRK1.1 in Strawberry
Source: Genes (Basel). 2020 Apr 16;11(4):427. doi: 10.3390/genes11040427 (PMC7230852; doi:10.3390/genes11040427)
Supplement: Supplementary file 1 [file genes-11-00427-s001.zip › Supplementary File S3.docx]

Supplementary File S3: Genomic sequences of strawberry SnRK genes.

>FvSnRK1.1

ATGGATGGAGCAATTGGCCGTGGAGGCAGCAGCGCAGACGCGTATTTACCGAATTACAAGCTTGGAAAAACTCTTGGTATTGGTTCTTTTGGCAAGGTCAAAATTGCCGAGCATGCATTAACTGGCCACAAAGTTGCTATCAAGATTCTTAACCGGCGCAAGATAAAGAACATGGAAATGGAAGAGAAAGGTAGGCCTTGTTCAGTTGATGCTTTCCTAACATCCATGGTTGTAGTTTATCATATGAATTTGATTGTTCTCATTTTGATGCAGTGAGAAGAGAAATCAAAATATTAAGACTGTTTATGCATCCTCACATTATACGACTCTATGAGGTCATTGAAACACCATCAGACATTTATGTTGTTATGGAGTATGTCAAGTCTGGAGAGCTTTTTGATTATATAGTAGAGAAGGGTAGGCTACAGGAAGATGAAGCTCGTAACTTTTTTCAGCAGGTGTGTGACAGTCTTGTTATCTTTCTCTGCGATTAGTTTGCATCTTATATCAGTTATGTATGATCTTTGTTTTTCCTGTGAGACAGATAATATCTGGTGTGGAGTACTGTCACAGAAATATGGTTGTTCATAGAGACCTGAAGCCAGAAAATTTGCTTCTGGATTCCAAATGCAATGTGAAGATTGCTGATTTTGGCCTAAGCAATATTATGCGTGATGGCCATTTTCTTAAGACAAGTTGTGGTAGCCCAAATTATGCTGCTCCTGAGGTATGGAGACTGTGTACTGTGTTAGATGACTTGTAAATTTGTTAAGTAAAAAACTTGAGTTATGTTACAAGATTTCTTGATTTCCATCTGTGTAGGTTATTTCTGGCAAACTATATGCGGGGCCTGAAGTAGATGTATGGAGTTGTGGTGTGATATTATATGCTCTTCTTTGCGGTACTCTTCCTTTTGACGATGAAAACATTCCCAACTTGTTCAAGAAAATTAAGGTAATTTAAGTTAACTTTACACCTATATTTATTTGGGATTATACCTCATAGTTTTAGGATATATTGAAGTTGTTTAATGGTTATATACTGTCTCTGTTGAATAACATAATATTGGCTACAGGGCGGGATATACACTCTTCCAAGCCATTTGTCACCTGGTGCGAGGGATTTAATCCCACGAATGCTTGTGGTGGATCCAATGAAGCGAATGACCATCCCTGAGATTCGTCAGCACAAATGGTTCCAGGCTCATCTTCCTCGTTACTTAGCTGTGCCCCCACCAGATACAATGCAGCAAGCGAAGAAGGTTGATTATTTTCTTCTGTTTCAAAATGTGGAGCTAGTTTCGTTGAGAACTGTTCTGGTATATCTGTCTGTACTTTTTGTAACAAGAAGTTAATGGATCACCATACCATCCCCCCCAAGAATTTCATAGTGACATGTCTGTAGTCCTTATTTTAAATTTATGCTTCATAGTCTCGTTCATGCACCCTGACAAAACGACAGTCATCAAAATTGCTATTCCTGTAGGCCGTTTATCTTTACCTATGGTCCATATCCCACACCTCGGCCTTTTTCTCTGTGATTAAAAAAAAAATTTGTGACACTTTGGACCTTTTTTTTTTTTTTTTTCTTCTTCCTATAATATAAGTTGCGGGGATACATCTATTATAAAAGATTGCTCAAGTGTTTCCTTGCTTTTCGCAGATTGATGAAGAAATCTTACAGGAGGTGGTAAAGATGGGATTTGACAGAAACCATCTTGTTGAATCTCTGCGTGGTAGATTACAAAATGAGGTGGGAACTTCAAAAACTCATTATCTCTTTGATTTTTAGCTAGTATCTTATTACTTACTGTGTTGCAACCTGTTTCAGGGAACCGTTGCTTACTACTTATTATTGGACAACCGGTTTCGTGTATCCAGCGGCTACCTTGGGGCCGAGTTTCAGGAGACTGTGGTATGTACCGTTTCACTGCCCTTTCTTGATGTAGTAGCAACCTATTGGTTGGAAAATAAAATATTATCTTTTCATGCCAAAGTAAAGTCCTTTAGCCGATATCTGATTAATAACTGATTATAGAGACACTACTAAAAATTGTTGAGTAAAATGATGATTTCTGAATATGAAAGAAAACTCTATCAAGAGATCTCACATGATTGTGGTCAAGTCATTGGAGATATTTCTCACATGATTGGAGAATCCTTCAGTAGTTGGCTACATTTGATATATATTTCGGTTTCCATTTTCTTTTCTAATTCTTATTTATATTTTTTGTCTAGGAATCTGGTTTCAATCGTATGCATCAAGGTGAGCCTGCTTCTTCACCTGTTGGGCACCGCCTTCCAGGATATATGGAGTTTCAAGGAATGGGCTCTTCACCTTTTAGACAGCAGTTCCCTGTTGAAAGGAAATGGGCTCTTGGACTTCAGGTTGATATTTCTTTGGCTTCATAACTGTTATGTGGAAAATGGACTAGCGTTGATAATCTATTTTGCTCTGTGATAAGTGTGACCAAATTAAATTAATAAGTATGTTAGAACAGTCAAGGCTATAGCTGCAGTTAATAGGTTGTGTTGCTTGATTAGCATGAAGTCTAGTATTTATTTTGAGTTCACATAGTTGTCTTGGTCATTCTGAGCATGTAGAGGCAGATTGAGCGGTGTCAACTTCTGAAGCAATAATTTCTTGGTTTTTGATCAAATTTGTTGCCTTTATACATGCAGTCTAGGGCTCATCCTCGTGAAATAATGACCGAAGTCCTTAAAGCTTTACAAGAACTGCGGGTGTGTTGGAAGAAGATAGGTCACTACAACATGAAATGTAGGTGGGTTCCTGGCACACCTGGTCATCATGAAGGCATGGTCGACAATCCTGTAAACAATAACCATTATTTTGGGGATGAATCCAGCATCATAGAAAATGATGGCATTATGAAGACACCCAATGTTGTCAAGTTCGAAGTGCAGGTATAAAATTAATATTGATGGCTCCTTGAATGTCAGTATGTACCAATTTGGTTCTTTGTTTTTAAGAAATACAGTACTCTTGATTGCTGCAAGTGGTAAACTGACAAGCAATTTTGACGGGAAATACATCTATTCCACTTTTTTTTTTTTCTACTTTTACAGTCTTTCAAAAGAACTTAACTGGTGATTTATTGGCCAAGTAGAAAAACCCTTTGATAGTATGCCAATCTGTACTGGAACTTGCTATTTTTTCTATTGAAAATAGTTGCTATCAATGATGCTAAGTAGCACAGGTGTACTGTTCAATTTTATCGTTTTCAAAATATTGTGCTGATATATCTTGCTGCTCTTTTGCATTCAACAGCTTTTCAAAACTCGGGAGGAGAAGTATCTGCTAGATCTGCAGAGGGTCCAGGGTCCGCAGTTTCTCTTCCTGGATCTCTGTGCCGCTTTCCTTGCACAGCTTCGTGTTCTTTAA

>FvSnRK2.1

ATGGAAAAGTATGAGCTCGTCAAGGACATAGGATCCGGCAATTTCGGCGTGGCCAGGCTCATGCGCAACAAAGAGACCAAAGAGCTCGTCGCCATGAAATACATCGACCGCGGCCTCAAGGTCCCCTCCCTTTTCCTTCCATCTTTTGCTTGCTGCTGCTTATATATCTATATTTATATATGGATTTGCAGATTGATGAGAATGTTGCCAGAGAAATCATCAACCACAGATCACTCCGCCACCCCAACATCATCCGATTCCGAGAGGTTGCTTTCTATTCTCCTCAAACCCTTTCCAAATCATATCAACCAAAAATTCACACCAATCTAAAATTTGCAGGTGGTTTTGACTCCTACGCATCTTGCTATTGTTATGGAGTATGCCGCCGGCGGAGAGCTCTTTGAACGTATTTGCAATGCTGGCAGGTTCAGCGAAGATGAGGTATCAATAACATCACACATCACAGAGATATAGAGAGAGAGTTTCGATTACAAATCCCCATTATATTTTGCTTTGTTCCTTTCTTTCGCAGGCTAGATATTTTTTCCAGCAGCTTATCTCTGGTGTTAGCTATTGTCATTCTCTGGTAAGATCACCACTCCATTCATTGTTTCTGCCTTTACCTGTCATTCTGCTTGTTCACATTTCTTGCTCTCTCTGCAGCAAATATGCCATCGAGATTTGAAGCTCGAGAACACCTTGCTTGATGGCAGCCCCGCTCCGCGCCTCAAAATCTGTGATTTCGGTTATTCTAAGGTTTATCTCTAAATTCAACATTAATGTTTCTTTGTTAATTTCAGAATTGTTATAAACACACATCAGCTTTGTTTTTATAGTCATCTTTGCTGCATTCACGACCAAAGTCAACTGTGGGAACTCCAGCGTATATTGCACCTGAGGTTCTTTCTCGAAGAGAGTATGACGGCAAGGTAACTTCAAACAAACTTTTTTCTTTCTTGTCACGAATAGCTTAGCTGGAATTGGGTCAAGGGTATCATCAGTTCCCCTGCTAACCAGAACACAAATCCTAATTTCTGCATCTAACTGATCACATATCTGACAGTCAATGCACCACAATTTGTTAAAGTAGTAACACTTCATTATTATGAGACATGAGTTTCTGGTTGCATCTACATCTTTAAAGCTATTTTTAGTGTCCCAGTGAGGGGTTTAAGACATTTAGTAGTAGTTTTCAGATGATTTGAATCTTGTAGTTATAAATTAGAGGCTGTATGGTTGTTTGAATTGGTGTCTATTTAACTCCGGTTATGTAACAACACCAAGTAAATGCATGGAACACAACTTACAAACTATAGCTTTCTATATAAAGGAAATTAACACTTCTCTCACCAATTTTCTGTCTAAGATATATCCAAATTTATTCCCATCAAATGTAGTTCAGTACTAAGCGATAGGGAATCATGATGGTAGCAGACATGTTTTGAAGGATTTGTAAACACAAAACATTTTTGCCTTTTTGTTTATTCATCTACTCTTTGAAGTTTGCAAGTTGTAGATTTGTTACAAGAATTTGAGACCTAATTGCAGTAGCTGGTTATGCTTCGCATCAATTACCAGGGTGCGGTTATGCTTTGCCATTGTAAACTAAACAGCCATTTATTTAGCTTTTAATATACTGCAATAGTAATTTTTCAGCTAATCTTAAATGTACGGTTCTGTTTCTATTATTTTGTTATTGAGTTGGTTTCTATTGAAAGACCAAAATTGCATTAGTTTCATTATCTTCTTCTATGTTGCTTAGCATATGCTAGTGATTTTCTATCAAAACAATGGAAAAATGCTATGTCAATAATGCCATGATCACTATATATTTCATATGATTCTTTTCTGGGGTGTAAAGAATATGCTCCTTTGCATTTTATATACGAATTCGTTATCCTTTTTTTAGAAGAGCAGAATGTGAAATGATATTCTACACTCATCTTAAACATGTTTTCTCCTGTCCTGATATTGGTTTCTGTTTATTCAGTTGGCAGATGTATGGTCTTGTGGGGTGACCCTATATGTTATGCTGGTGGGAGCATATCCATTTGAGGACCAAGAAGATCCAAAGAATTTCAGAAAAACCATTAATGTATGTTTGCCTTTTTATTTGACCTATCCTGACTGGGTTTACCTTTTCCCCTGTACTAAACTATATGGTTTTTTTTCATTATACGATGCAGCGAATAATGGCTGTTCAGTACAAGATTCCTGACTATGTTCACATATCTCAAGATTGTAGGCATCTCCTCTCTCGCATATTTGTTGCAAATCCAGCAAGGGTATGTTGCTGCCTTGGAACTTTGAATAATTTTTTGCTAAAAATGCTCGTGAATGTGTATAAGAATGAAAATTGGTGTATTTTTTGTGAAGACTAAATAACCTCTCAAATTATCCCGCACATAAACGCTTTTAACACACTGTTACTGCGCTAGGTTTGTTGTGGAAATGTTGAGGCCTGAAGTTGAATTCTTGGATCACAAATTAATTTTCTTGGTTCGTAATTGCTTTTACATAAATGTAGTGTATATGCTCTACAATCTTGCCTTGCTAAGAATTATTCTGTAGACTGCCTGGAAATAATTTTAATTGCCCATTTAACTGCATCTCTATTGTAAGTGTTGCATCACTTATTTATATCACTACCTTTGTCTTGACGATTTTGATATTCTGGCATTTGCTACAACTGGAAAGTCTTTTTTCAAATTTTGAAATATTTGTAAACAACAGGACACTCATATGCATATATAAGTAATAATTTTAGCCTGCAGTCTGAGTGTAAATAAATGAAATTAAAAAACTGTGGAGATATCAATATTCTGTAGGGAGAATGGGAGAGACCGACAAGGCCATGAGATCTTTTTGCTTGTTTTATACATTAACTAGCTCAAGTTTCTCAGCCTGATCTTTGATTCTATCAGTTAAGCACAATCTCTATTGTTTTTATTTTTAATCATGGTCCACTCAACTGCTTCAATTCTGTTTTCAGAGGATCACCATTAAAGAAATCAAGAACCACCCATGGTTTTTGAAGAACTTGCCGAGAGAGCTCACAGAAGCAGCTCAAACCATGTACTACAGAAAAGAGAACCCAACCTTTTCCCTCCAAAGTGTCGAAGACATCATGAAGATAGTGGAAGAAGCCAAAAATCCTCCCCCAGTTTCCCGGTCGGTTGGAGGCTTTGGCTGGGGAGGAGAAGAAGATGGTGATGCAAAGGATGAGGTTGAGGAGGGCGAGGATGAAGAAGATGAGTATGAAAAGAGAGTCAAAGAAGCACATCAAAGTGGGGAAGTACGTGTTGTCTGA

>FvSnRK2.2

ATGGAGGAGAGGTATGAGCCAATGAAGGATCTTGGGTCTGGCAACTTTGGAGTGGCGAGGCTGGTCAGGGATAAGAAGACCAGGGAGCTTGTGGCTGTGAAGTACATAGAGAGAGGCAAGAAGGTTGGTTTCTAATGTGTGCAAGTGTTTTGGTATTTGAGTGAGGATTGGAATTTCTGACTGAGTTTGGTGATGAAAAGCCTCACACTGGGTTTTGGTTTGGGATTCTGTTTCTCATTCGTTTTATCAATGATGTTGTTATGGTTTTGGATGTTAGTTAGGTGAGATTTGAGCTGAAGTTGACTCTCATTTTGATCAGAAACCCGTGTTCTGCTTTTTTTTCTTTTCTTTTCTTCTTTCTAGTTTCAGCTGCTGAATATTTAGATACGGAGAAGAGTTGGTGTGAGATTCCTTTTAGCTCTAAAAGCAGATCTTGGTTCTCAAATTTGGACCAACCAATGCTATTGTTAAAGTTCAAGTCTAAATCTTTATGCTTGTGCTGACCATGTCACTCTTTCTTTTCTGCAATATTGCTCCCAATAGCAAAGTTCATTTGTCTTTTCAGTTAATTATTGCTAAAAGTCATTACTGTTCTGAATTAATTTTGAGCAACCATAGCTATGTCAAGTTGTTTAATCATAGAGTATTAGATTTAGCTCATGGAGTATTACATTTAGCTCCTATCTGAATTTCATGTGTGTTTGATGTTTATTATCATGCAGAGTTGTCCCTCAGCTTTTGTTGCACATTAGACATGATTGTTTTTTTTTTTGGGTATCATTATGACTTCGAAGGATATGATTATATCCTTTTATTGTTGATCAAAGCCAGTTATGTTGTTAGGCTATATTTACATATGTTAAGGCCAAATTGCCTACTGCATGTCAAATCAAATATGAATCCTGTTATTCTGTTCTCGTTATCTTCCCATGTATGTATATGTAAGCTTATTATCGATATGAATGTTGTTTTGGTTTTCCGGTTTGCAGATTGATGAGAATGTTCAGAGGGAAATTATTAATCACAGATCCTTGAGGCATCCTAATATTGTCAGGTTCAAAGAGGTAATTAACTAGTATTGAGTTGATTGTGTGCGTACATGCTCTTTGGAGTGTGTATGGTGTGTTTAAGTATTCGAGCTTGTCATTTTGGTATTTTGGAATATCAGACTAATATCATGTTAAGATAACTCCAAGATAATATATCAAACTCGACGTGAGCACCAAAGAGCTTATATAAGGCAGTCTTGTGTTGCATGCTTGAAGTATAATTGTTTTACTGCAGGTCCTGTTGACTCCAAGTCATCTAGCTATTGTCATGGAATATGCAGCTGGTGGTGAACTCTTTGAGAGGATATGTAGTGCTGGTAGATTTAGTGAAGATGAGGTTAGCTTCTTCTGTTTACATGTTAGATGAAATCGTTAGGTTTTGCTGCATTAAGGAATTACTTGTTTAGAAGTTTTGATTTATGAGTATTTGTTAACTGCAATTCCTCCACACAACAGGCAAGATTTTTCTTCCAGCAGCTGATATCTGGAGTCAGCTACTGTCATTCCATGGTAATATATTTGATGCAGATACATTGTCAAATCGTTGTCTTGTTTAGTAATACTACTGCTTCTTTTAATCTTTTCTTACATTAGCCAACGATTATTCTGCAGGAAATCTGTCACAGGGATCTGAAGCTGGAAAACACACTCTTGGATGGAAGTGCGACACCACGTCTGAAAATTTGTGACTTTGGATACTCCAAGGTTTATGATTTTCTTGTTGAAATAAGACTTCATTTGGTTGATATGCAAATTTAATTTCTAACTAGTCTATTGGGGCATCTTAGTTATCTGATATACTATTATTTACTACCTTGCAGTCTGCTATTTTGCATTCGCAACCAAAATCAACTGTTGGAACACCTGCTTACATTGCTCCGGAGGTTCTGTCACGGAAGGAATATGATGGAAAGGTATCTAGTTTGTCCATATTGTTTCCTTACCAGTCTAGGAAATTCAATCATCTTAGATTAAAATTTCCAACTGTCATATGTGTTAAACAAATCTCAGTTACAGTATAATCTAAAACTCAATAAGAGATAATATCAGTTTTATTTTCTCTCTAAGTGGGTGAATACTTTGGTAGATTGCAGATGTTTGGTCCTGTGGTGTGACACTATATGTCATGTTGGTAGGAGCATATCCTTTTGAGGATCCCGAAGACCCTCGAAATTTTCGTAAGACCATTGAGGTGAGCTTCATCCAAACACTACATAGTTGGACATATATCTAAATTTCAACATGCTATACACTTGTTTCCGCTAACAATTAATATGTTAATTCCTGAAACAGAGAATTATGAGTGTCCAGTACACCATACCCGATTATGTTCGTATTTCAGCAGACTGCAAGCACCTACTGTCTCGTATTTTTGTTGCCAACCCATCTAAGGTAATTTTTCTTCCTACATACTTCTAAATTTTCCTTGTTCACTTGAGAGTTGAGACACATTATACAGGGAAATGGTCAAATGGATGAATAAGAACAATACTATAAATGTGGTGTTCAAATTGCTCTTACAGTTAAATTCGACCGTTCTAATTAGTCTGATTTCGAAACACAATCTTAACTGTCGATGGATTCTCTAGTTCTGTACTGATATTCGTATTTGTATTCTCACTACAGAGGCTCAGTCTTCCTGAGATAAAACAGCACCCTTGGTTTCTGAAAAATTTGGCAAAAGAGCTAATTGAGGTTGAGAAAAAAAGCTTTGCGGAAGTGGAACGTGACAACCCGACACAGAACATTGAAGAAATAAATAAGATCATACAAGATGCAAGGACACCAGGGGAAGGTTCCAAAGGAGTTGGCCAAGCTGTTGCAGGAGCAGGACCATCCGACTCTGATGATGTAGATTTGGACTCAGAAGTTGATCTTAGTGGTGACTTTGCTTGA

>FvSnRK2.3

ATGGATCGGTCTATGCTGACAGTTGGGCCGGGTATGGATATGCCGATCATGCACGATAGTGATCGGTACGAGCTGGTTAAGGATATCGGGTCGGGTAATTTTGGGGTGGCCAGGCTCATGAGGGACAAGCAGACTGAGGAGCTTGTTGCTGTTAAGTACATAGAGAGAGGTGAGAAGGTTAGTCTCTTTCTTTCTATCTTTTTTTCTTTAAACTTTCCTTCTTTTTTACCTGAAATGGGGTGTGTGTATTGGTTTTGTTTCTGTCTATTTTAGGAAATTTTTGAAGGGTTTGTTGGCTTTTGTGTTGTGATTGATGGGTTTTGTTGTTTTTGTAGCATTGTGTTGCTTTTGTGATTGGGTTGGTTTTGTTGTTCATATGAGGCATTGTTTTTCATGTTGGTTTTGAGATGGTAAGTTTCTAGATACAGGCAGTTGTGTGGTCTTTCCAGTTTTGAGCCGGTTCTTTTTTGGAAGTTACAGTTGAGGACCAGTAAGGTTAAAAAATTTTGGGACTTTTTTCTTTGCGCAACTTTGTTGAAATGGAGTTTTTGGTTCAATGTGACTTCACTGTGTTACTTGTCTGTGGAATATGTTTTATTAATCCTGTTTGATTGTTTGATTGATGAGCTTGGGTGTGAGCATTGACAACATTATTAGACAACCTCATTAGAAGTTTTGAGCGCCTTTGGTGAACTATTTTAGTTAATACCAGTCATATTAGCGAGGTTTTGTAGTCGAGTATCATTAGTGGACATTCTTAGTGCTTGGGGTGCTTTGGTGAAAAGGCTAGAAACTCAGCAATCAAATTGATGATGGAAGTAAAACATCTGCCATTTGGCAAACTGTTGCGTTACCATGGGGAAAGAATAAAAGAAATTTGACCTTACTTTTCTCAAGCGTAAACTGTAAAGGAGTTTACGTTTTCTTTTCTTTTGTATAACCATATACATATTTTTTTGTTTGAATGGTTGAATAGAAACAAGACATTATTGGTATAAGGAGAAATTATACAGCAGTGAATAAAAAAATCACACTAAAAGAAACCTCAGGAAACTGAAAGCTCCCAGAATAGAAAAAAGCTAGGACTTTGAACAAGACAAACTTAAAATCCTAGAAAATTGTGTGACCTGCCAAATGTTGCGAAATAGAAATGCTGAACTAAAAAGCAACCAAGGCCCAATGGTTGTCAAGTGTATGGTAAACACCTAGTGATCTAGTTTTTCGTGTTTTAACTCTATCAACAGTCAACGAATTGTGGAATACTCGTAAGATTTCCCAAAGAATTTGACGTTGGCATTAATAACGTTTGTGACTGCAGTTTCTTGGTAAGTAATATTATCCTTGGATTCATTTTAAATCCTTAGTATTAGAGAGATTGCACAGATCAAATGAAGAGAGTGAATCGATCTGTGTTGGGGGAAACCATTCTCTTGATTTGATCTGTGTAATCTCTCTAATACTTGGATTGTCATTATTAATAATTCTTTTTGAATGTCAATGAAACTTGTCAATTTAATCAGTAAACTTGTCATTTGATCAGCCAAGATATCTTCATCGATGCTGATCAGTTTTAAATTCTTCATTGTCCTGTCATGCTGATGTCCCACTCTTGTTCTTGTTAGCTTCAAATTAGTGTCCTTTCAATTTTGATTCTCCCTAATGATATATTTTTTTTCTCTAACAGATAGATGAAAATGTACAAAGGGAAATTATAAACCACAGGTCATTGAGGCATCCTAACATTGTCCGATTTAAAGAGGTTCGTTGAATTCTAGCATCCTATTTCATATTGTTCCCCACAGAGTTGAATAATATCAGTACTTGGCTGTAGGTAATATTGACACCGACTCATCTGGCTATTGTGATGGAATATGCATCTGGAGGAGAGCTCTTTGAGCGGATATGCAATGCAGGGAGGTTCAGTGAGGATGAGGTACTTATCCAAAACGTTTATGCTGTATCTAATAGTGTCTCCAACCAATTTGTAACGTACTTCTTCTGTTGCAGGCACGTTTCTTCTTTCAGCAACTTATATCAGGAGTCAGTTACTGTCACGCAATGGTATGTAAACAAAGGGGATTCATCTTTATTCGATTCTTTAGTACATAAAATATATACAACTAGATTTCAAAGCGGATATGTTTTTCTGTTCTTCCTGAAGCAAGTGTGCCATCGGGACTTGAAGTTGGAGAACACATTGTTAGATGGAAGTCCTGCTCCTCGTTTAAAAATATGCGACTTTGGCTATTCAAAGGTACTATATATATATTTATATGTCCCCCCTCATTTTTATGTAAATTATGACAGATCTCTAGTGATTCAATTCAAAGCAGAAATCCATGTATATAAGACATGGCTGTTTGAATGCTTATATGCATGTTCCTTAATTTCCTTAATTTATGGAATTTTCACGATTCTGATAATTTCTTGGGTGTCTACAATCACTTCATTTCTTATCTGTTTTTTCACTTTTTTATTTGGTAATGTTTATATTTGTTATATCCATCTAATTAGGCTTTCTCTCTTGTCTCAGTCTTCTGTGCTACACTCGCAACCAAAATCAACTGTTGGTACCCCTGCATATATTGCTCCCGAGGTGTTACTTAAGAAAGAATATGATGGCAAGGTAACAAAATTCGTTCATTGAAATCCTAGTTTTTCCTTGTTTTCCAAATAAGTACACTTTTGTATTATTTTAATCGTTGTATATATATGAAGCTATAAACATCAGCTGAAATCCCATGCTTCAAACACAATATTTAGATTAACATTTTATTGGTGTCAGAGGGCAAGTACTGGGACTTCTGCATGTAATTATGTAGAGTCATGGATGATGGACATTCTTTGCCGTATCCATGCATTGACATAATCATTTGATTCCATTTTCTTTTGTCCTGGAAAAAATGAATACATTATGCTAATATTTCTCTGTTGATGTCATTCAGATTGCAGATGTCTGGTCTTGTGGGGTGACCTTATATGTCATGTTGGTGGGTGCATACCCATTTGAGGATCCAGGGGAGCCTAAGAACTTCCGCAAGACAATACATGTAATTGTTTTTCCACCTTGCAAGTAATTTTGCCCTTTCACGTTGTTACTATTGAGCCTGGGCAGCTCTAATGTGGTAGTGTGGTTGACTAAGATAATGGATTTCTTTGCAGCGTATAACGAGTGTCCAGTACTCAATTCCCGACTATGTTCATATATCTCCAGAGTGCCGTCATCTGATCTCAAGAATTTTTGTGGCTGAACCGGAGAAGGTGGGTTACATTCATTGTATATGAGTTACGTTCTTGCACTTATACATCAGTTTCATATTCCAATTAAATCTACGTTCTGCTTGCTTATTCTTGAGGTCGTAACATTTAGCTATGGTCATGTATGTCTTTTCTTTTCCAGTGAAGTCATGTGTTGACATATTCTTATCATCCTCTCTTTGTGAAACTACCTCGTTTCACAAAATTATTAAACAATAGATTGCATCCTTTTTCTTTCTTTTTTTAATTTTTTTTTAAAAATTTTATATACTGCATCCTTCTTCAATAAAAAAAGGTGTGTGAATAGCACCACTCTATCTCTATCTCACCTATCTAATTTATGCTTTTATGCTTTTATCATCTCGCTTCAACATCATGTTTGAAGGACAGTACCATTTTCTAATTCGAATCTGGTTGAATATTATTGTCTGCAACAGAGGATAACAATTCCTGAAATAAGGAACCACGAATGGTTTCTAAGGAACCTTCCAGCAGATCTCATGGTTGAAAACACCATGAACAGCCAGTTTGAAGAGCCCGATCAACCCATGCAAAGCATAGATGAGATCATGCAAATTATTGCGGAAGCTACAATACCGGCTGCAGGGACTAACAATCTCAACCAGTATCTTGCTGGCAGCCTGGACATCGACAACATGGAGGAGGATCTTGATACTGATCCTGACGACCTTGACATCGACAGTAGCGGGGAAATAGTGTATGCAATTTGA

>FvSnRK2.4

ATGGAGAAGTATGAGTTTGTCGAGGATATAGGATCAGGCCATTTCGGCGTCGCCAAGCTCATGAGAAACAAAGAAACCAAAGAGCTCGTCGCCGTGAAATACATCGACCGCGGCCTCAAGATTGATCAGAATGTTGCCAGAGAGATCATCAACCACAGATCACTTTGCCATCCAAACATCATTCAGTTCAGAGAGGTGTTTTTGACTCCTACGCATCTTGCTATTGTTATGGAGTACGCCGCAGGCGGAGAGCTATTTGACCGAGTTGCCAATGCTGGAAGATTGAGCGAAGACGAGGCTAGATTTTTTTTCCGGCAGTTTATCTCAGGTGTAAGCCATTGTCATTCTATGCAAATATGCCATCGAGATTTGAAGCTGGATAATACTCTGCTGGACAGGACGAGCTCGTCTCCACGCCTGAAAATATGTGATTTCGGTTTTTCCAAGTCATCTTTGTTGCATTCAAGAACAAAGTCAATTGTGGGAAGTCCGGCGTATACTGCACCTGAGGTCTTTTCTCGAAAAGAGTATGACGGCAAGTTGGCGGATGTATGGTCTTGTGGGGTGAGCCTATATGTTATGGTGGTGGGAGCATATCCATTTGCAGACGAAAACGATCAGACGAATATCAGAGAAATCGTTAAACGAATAATGTCTGTTCGATACGTGATCCCCGGCTATGTTTACATATCTGAAGATTGTAGGCATCTCCTCTCTCGCATCTTTGTTGCAGATCCAGCAAGGAGGATCACCATTCAAGAAATCAAGAACCACCCGTGGTTTTTGAAGAACTTGCCGAGAGAGCTCACAGAAGAAGCTCAAACCAGAAAAGAAAACTCGACATGTTCCCTTCAAAGTGTTGAAGACATAATGAAGATAGTGGAAGAAGCCAAAAACATTCCTCCCCCAGTTTCCCGATCGGTTGGAGGCTCTGGTGATGCAGAGAAGACGAGTATGAAAAGAGAGTCAAAGAAGCACATCAAAGTGGGAAAAGAGATAGAGGAGGGCGATCTCAAGAAGACGAGTATGAAGAGAGAGTCGAAGAAGCACATCAAAGTGGGGAGGTACGGGGTGTGTCGGATGAGCAGCACCTGA

>FvSnRK2.5

ATGGAGAGGTATGAGATAGTGAAAGATATTGGTTCTGGGAACTTTGGTGTTGCAAAATTGGTGAAGGACAAATGGAGTGGTGAGCTCTATGCCATCAAGTTCATTGAGAGAGGCCAGAAGGTAGAGATTTTTTTTTTTTCATCCCCTATTTTTTCTTGATCGAAGTCTTACTGTTTTGGGTTTAAGCTCATTAATCTCTTGTGGGTTTGTTTGTTTGTTCATTTTTGTGATCTGAGTTTGTGTTTATTCCAACTGGGGTTTTGGTTTTAATGAGGAATTTCCTTGTGTTTTTTTACAGATTGATGAGCATGTACAGAGAGAGATCATGAACCACAGGTCACTGAAGCATCCAAATATCATAAGATTTAAGGAGGTATGATTCCTGTGTTTGATCACACCTTTCTCAATGATATGTTCTCTTTTGTGGAATAGTACTATTGGTATCTCAATGAGAAAAGATTTGTGCCACTAACCCTATTGGCCTGCAACTTAGAAGAATTTTAGAATCAAACTACTTTTTGGTCCATCATTCAACATTATTTTTCAAACTGTTTCCAAAGACTCATTGGCCTTGGAAGAGGGCTAAAGTCAAATATTCATCATTTCCAATTTGGCTTTGAATTGTTCTGCCTCTCTATCACTTGTTCTTGTTTTATTTAAAAACTTGCGGAAAGTTTCTTGGTACAGTTTTTCTAGTACTGTATATCTTATTTTCTGCTCGTATGTGCAGGTCTTGTTAACACAAACCGATTTAGCGATTGTCATGGAATATGCAGCAGGAGGAGAACTTTTTGAAAGAATATGCAATGCTGGTAGATTCAGTGAAGATGAGGTAATTGGAGCAAAGGCTGAAATGAATGGGAGCAGATTTTCGTCGCGATAAATTTATTTCTGTACATTTAGGGTATGATAATTTCGTTTTCTTCCAACCACAAATCATTTGGACTCTTAGTAGTTGATTTGAAATTTCAGGCCAGATTTTTCTTCCAGCAGCTGATTTCAGGAGTCAGTTACTGTCATTCAATGGTAAAAAGACTAACATAAAAACTGTAACATAATTTTCTCTCTGGAAATATGCTTGGCCTCACATCCTTTGCTGGTTTAATCTGAATGTATTTTTATTGCTGCAGCAAATCTGTCACAGAGATCTTAAACTGGAGAACACACTTCTAGATTCAAGCTCAGCTCCACGTCTCAAAATATGTGATTTCGGTTATTCCAAGGTAAATGAACTAAGCCGAGCAATCATGTTGAGTAGGGGTAATTTTAGTTACAGTTACTAATTACCTTTCTGCTTCATGTTATAGTCATCTGTGCTGCATTCACAACCCAAATCAACTGTAGGAACACCTGCCTATATTGCACCAGAAGTCCTGTCAAAAAAAGAGTATGATGGGAAGGTAGTTTGTTGGTTTCTTTCTTCTGTTTTTGAGTTGGCCTTATTGTGGACCAAATTTTAGGTCACCGAATTATTGAGACTCTTATTTTATCTTTTGAGAATCTTAGATAGCAGATGTTTGGTCTTGTGGGGTTACCCTATATGTGATGCTGGTTGGTGCTTATCCTTTTGAAGATCCAGAAGATCCCAGAAATTTCAGGAAGACACTTCAGGTTCATTGTTTTATTGTAGTAGCATTTTAGCTAGTTTTCCAATTCTCGAGGCTCAAGACATATAACATGCTAATGGATTTTGGCAGCGAATTCTCAGCGTCAGCTATTCAATTCCTGACTATGTACGCGTTTCCAGGGAATGTACACATCTTCTTTCTCGAATTTTCGTTGCTAACCCCGAAAAGGTTTTACTACTTCTTAGAATCATAGCCAGCGATTGAATGTCTGTCTGTTGATGTACATTCCAGTTGAGTCATTAGAAATGTTTACCTTAACATTTGAATTCTATGCAGAGAATTACAATCCCAGAGATAAAGCAGCACCCCTGGTTTCTGAAAAATTTACCATCGGAATTCATGGATGAAGATGACATGCAGATTGGTGAGGTTCAGAAAAATGAGATTTCACAAAGTGTTGAAGATATAGTGTCCATTATTCAAGAGGCTCGAAAACTTGGTGATGGCATCAAAGTTGGACATTTTCTTGGGAGCATGGACCTTGATGAGATAGATGATGCCGACATCGATGATATAGAAACTAGTGGTGATTTTGTATGTGCGTTGTGA

>FvSnRK2.6

ATGGAGAGGTATGAGATTGTGAAAGATATTGGGTCTGGGAATTTTGGGGTAGCTAGGTTGGTCAGAGATAAGTTGACCAGAGAACTCTTTGCTGTTAAGTTCATTGAGAGAGGCCAGAAGGTCAGACTTCATCTTCTTCACTCCTTTTACTTTTACCTTCTTCTTTTTTTCCCTGAAATTTTCTGGTATATTGAGTTTCTGTGGTGTTGCAGATAGATGAACATGTGCAAAGGGAAATCATGAACCACAGATCATTGAAGCATTCCAATATTGTTCAATTCAAAGAGGTAAGTGATTAGTGGTGAGTTAGGTACTTGGGTCATTTTTCATTTAAAAATGATAATGTTTCTCAAAGATCAATTTATAGAGGATTGACCAATGACCTGTTTTGTTTAATAAAGACAGTATATTATGATCTGTCTAAAGGAGAAAACTGTGAATGAATAATCTCTAAGCTTATGTAATGTCTTCTCCCACATAATCTCCTTGTTCCTTTTTTGAATGGCATGTCTATCAGATTCAGGTTTGTATAAGCTTGCTTTGCATCAGCATCTGTTGAGGTTGGATTGAAGACAGAACACGCTAATTTATGCTTCTAGTGTGCTTAAATTAGCGTACTTCCTGTCACTTCTTTAAAATGTTAAGCAGTAACATTCTATACCATGGCCAATTTCCGATCAATTCATGTGCTATAGTGATGGAATATTGGATGTCTTCCTGGCAGGTTCTTCTGACACCAACTCATTTAGCCATAGTAATGGAGTATGCTGCTGGAGGAGAACTCTTTGGGAGAATTTGTAATGCTGGTAGATTTAGTGAGAATGAGGTAAAGTAGAGGAGAACTCTTTGGGAGAATTTGCAATGCTGGTAGATTTAGTGAGAATGAGGTATAGTAGTTAACGAGGGGGTCAGGAAAATTATTGTATATGTCAGATTATGGGGTTCTTTTTGTTCCCTGAACATGAAATGGATATAAAGATTTACAAGCACTTTTACTTATATATCGGTCATAATATTACTAAAACTGACATTCTTCCGCAATTCTGTTGTTTTTTTGCTTCAGGCAAGGTTTTTCTTCCAGCAACTGATATCAGGAGTTAGTTACTGTCATTCAATGGTATGTCAATTGGAACTCTTCTACTAATGTGTGGTGTACCGTTTTTTTTTTTCTCCTAATGAAAGTATATAATTCGTTTGTGGCAGCAAATATGTCACAGAGACCTTAAGCTTGAAAATACACTCTTAGATGGCAGCACAGCACCTCGTGTCAAAATATGTGATTTTGGATACTCAAAGGTATTCTACAAACTGTATAGTAAGGATTCGGCATTGGGCGATCGGTTAATGAGACTGAGTAATTATTATTTCCCTTGGTTTTTGTAGTCACTACTGCAGTCTCAGCCAAAATCAACTGTAGGAACACCAGCCTATATTGCTCCTGAGGTCCTATCTAAAAAGCAATATGATGGAAAAGTAAATTCCAATTCCTGTGCCTTCTCATTGACACCATAAATTGTCATGGGTTAAGCAGCATTTGTAACTAATCTTAACATTATAGATTGCAGATGTTTGGTCTTGTGGAGTCACCTTATTTGTGATGATAGCCGGAGCATATCCTTTTGAAGATCCTGATGACCCTAAGAACTTCAGAAAAACCATTAATGTTAGTGACATTACAACTTTTCACCAATAAAATTGAACATGCTATGTATAGTGGAAAACAGTGTTATATCCCTCTAAACTGAATACTTTCTAGAGCTATGGCTAACAAGTTGTCTCATGCAGCGGATCCTTGCCGTACACTACTCAATACCAGATCATGTTCAAGTTTCCATCGAATGTAGACATCTTTTATCTCAGATATTTGTGGAAAACCCCGAAAAGGTAATAATGTAGCATCTGTTGAAGTGTTGAAGGTGGTAGACAATTCGAGTTAACTATTGAGGCATTAATCCCCCTAAAGAGGAAAAGAAAATCATGTGTGTTATAAGCGAACAATTCTATTCCTGACTTAATTAACTAGGTTCACTGCTAGATTGCTAGTTGTCTTGCAACCATTTCATACTCAGAGCAGTTCCATTCCTGACCAATATCTTGGCTTTGTTTATGTGTTTTGAGTTGATTTTTATTGGTAAGTGTTAGTCCTTTCATTGTGGTCTTTGCTCGCAGAGAATTACAATCGCAGGAATTAAAAGCCACCCTTGGTTCTTAAAGAACTTACCTATGGAGCTGATGGAAGGAGGAAGTTGGCAGAGCAATGATGTAAATATTCCATCCCAAAGCACTGAAGAAGTTCTGTCCATAATTCAAGAGGCGAGAAAATCAGTACCGTACCCAAACACAGTTACGCATCTCATTGAAGGCAACATGGATCTTGATGATTTGGATGCTGAAGTTGAAGATGTTGAAACAAGTGGTGATTTTGTGTGCCACTTGAGTAGTTAA

>FvSnRK2.7

ATGGAGAAGTATGAGGTTGTGAAAGATATTGGGTCTGGGAATTTTGGTGTAGCAAGGCTGGTCAGAGACAAGAGTACAAACGAACTCTTAGCTGTTAAGTTCATTGAGAGAGGCATCAAGGTCTGATTCTTCATTTTTCTCACCTCTTATGTTCCTTTTTGTGTTTCTGAGTTTTCTGTATTGGGTGTTCTTTTTCCCCCATTGTTTTGGTGTTGCAGATAGATGAAAATGTGCAAAGGGAGATCATGAACCACAGATCCTTGAAGCATCCCAATATTGTTCAATTCAAAGAGGTAAGTAGTTAGGTGATTTATCCACAGATTCTATTCCATCTTCTCTTCCAAGCATCATAATGCAATCTGAATTAATTTTCTGAGAGAGATAAGATGAGAAGAGAAGAAGAAATTAAGAATGAGTTTGCCTGCTACTTTATATCATTTCTTTATACACAAGTATTTTGCATTGAAAGATAGTGATTACATCTTCTATCGATTCTTCATCTCCATTGCATCAAGTATCTGTTTTCTTGGTCTGAATTATACTTTCTAAACATGCTTGAAATGGTACCTAGTAATCAGTTCTTTTCGATTACAGGGTTTATGACCTGAAAAATATGACCTTTACTTCCTCAGTCTCTTTTAATCATTCCAGGGTTTATGTTTAAGAGTATTGAGGTCTTTCTTTCATGCAGATATTGCTGACACCAACTCATCTAGCAATTGTGATGGAGTATGCTGCTGGAGGAGAACTTTATGACAGAATAGTCAAGGCCACAAGGTTTAGTGAGAATGAGGTAATGTAATTTTGGAGGGGGTGAGTCTATCTCATGACAGTTTATATGACTGGAATATGGGTACTCCACCGGTCCCATGGAAATGAGATCAATCCGAATTCTCTTTAAACTTTACTTTCAATTGGGAAAAAAGATCCATTCTGAAAGTAATTTTGACTTCACAATAAATCATTATATAGTTAAAAGTTTACAGTTGTTTGCATCAGGCAAGGTTTTTCTTCCAGCAATTGATATCAGGAGTTAGTTACTGTCATACAATGGTATGTAAAAGTTGCCACTCTTCAGCTAGTATGTTGTGTACTGCTGTTTCTTGTTTCTCATGATCTTTGGTATATTATTGACTGTAGTAATGAAATAATTCATTTTTGCTGCAGCAAATATGTCACAGAGATCTTAAGCTTGAAAATACACTCTTAGATGACAGCGCAGCACCTCGTGTGAAAATATGTGATTTTGGATACTCAAAGGTATTCAACAAATCCTAGAGGATCTGCAAATAGGATTTGGGGTATTGAGACTATAATTCATGGCTTTTCACGTTGTTTTGTAGTCATTACTGAGTTCTCTGCCAAAGTCTACTGTGGGAACACCTGCTTATATTGCACCTGAAGTTCTATCTAAAAAACAATATAATGGACAGGTAAGCCCAATTTTTTGTGCCTTCTCATCAACACTGTAAGTTTTGTGCTGCAGGCATTATGGATCATTTGTATCTAAAATTAAATTGTTATAGGTTGCAGATGTTTGGTCTTGTGGAGTCACATTATATGTCATGCTTGTCGGAGCATATCCCTTTGAAGATCCTAAGGACCCTATGAACTTTAGAAAAACAATTCGGGTGAGTAGCATTCTTACCGTTTCACCTGTCCACAACTCTTGTTTCCTAAATTAAGAGTTAAAACCTTATATATGTCTTATGGCAGAGGATCCTTACTGTACGCTACGCGATCCCAGATAGTGTGCGAGTTTCAGTGGAATGTAGACACCTCTTGTCAAAGATTTTTGTTGCAAACCCGGAAAAGGTAACATGCATTTCATTTCAGTGTTGTTTTGCTAGATAAATTAGACTTGTAACTTGAATATGCATATCAGGAGCCTCTTTCAATCTTGTTTTCCCTACTCGTGATTGCAAACTTCTGGTGTCTGATACACATTTCATGGTGAAACTTATGTGTCCTATTAGAATTCTCATTGTGGTCTGTCTTTATTTCTCTGTAGAGAATAACAATTCCAGATCTTAAGATGCACCGTTGGTTTGCAAAGAACTTACCTCTGGAAATGAAGGAAGGAGGAAGTTGGGAGAACAATGCTGTAAACACTCCATCCCAAAGCATTGAAGAAGTTCAGTCCATAATAAAAGAGGCAAGAAAACCTTTAAAGGTCCCAACTGTCAGTAGGCATTGCGTTGGAAGCAGCATGGCTATTGATGATGCTGAAGAATGA

>FvSnRK2.8

ATGCGCCAAGAACCCAGCCTTGTGCAAAATGTGCAACAGTGGAGCTGCGACACCATGATTAGTAGCGGCACCCCCACGGCCCCACCAGATATGGTAAAGACCAGGCCGAAAAGAATGACAGAATTGTTTTTATGTCGCGCGGTAAAAACTGGATCTGCAACTTGCATGGCCTCACATGATGATGATGATCTTCTTCTTTTTCAATGCTCTCGCTGTCCTTAACAGCCACAACATTTTGTCGCACATTGCACGAGTCAATCAATTTCTCAACTCTCTGCGGCTCTAAATTATTTCCAATACTTAAAAAAAGAAAGAATACAAGAATACAGATTTGTCCACAACACCCCTCACCCAACACGAAAATATCTACCGACTCAACTCACCTGTATTTTCCTTTTAAGGTCGAAAACCTCTCACTCTCTAACTCCCAAATTAGCCTAAGCCTAAACACCCTTTTGCGGTTAGACTAATTATTCATGCAAGTTGGTGTTTAACAAAGAATTAAGACTCTTGTGAGTTTTGGATTTTATGGAGAGGTATGAGATTTTGAAGGATATCGGAGCTGGGAATTTTGCGGTCACAAAGTTGGTCAGAGAGAAGTGTAGCGGAGAACTATATGCTGTCAAGTTGATTGAGAGAGGCAAGAAGGTTTGCTTGCTTCTTCATCATATTTCTTCACCGCTGGGTTTTCTGGGTATTCTTTATCATTTTATTTTATTTTATTTTATTGTTCTGGGTTTTGCAGATAGATGAAAATGTGCAAAGGGAAATAATGAACCACAGATCCTTGAAGCACCCCAATATTGTTGAATTCAAAGAGGTTAGTAAAAAGTATTGAATTTTCTGAATGTTCATGAGCAAGTGACTAATATTAGTAATGATTCTGCTTCTTCCCTTATATTGCATGTTTCTTTTCTTTGCCATTGACAGATACTGATGCTATGTTCTTTATCGGTTTCCCTTGAGCATTGTATAATCAAGTATGGTCATTTATGCTTAAATTGGTTGCTGAAATCATTTGTAGAGGAGCAAAATACCTATATTAAAGATTTGATCTTTACTTCTTCCACTCTTTGTTCATATGGAATGGAGGCTAATGCTGATAGATTGGTGTTTTAAGAGTTTTGAGGTGTCTTTCATCCAGGTCCTGCTGACGCCAACTCATCTAGGTATAGTAATGGAGTATGCTGCAGGAGGAGAACTGTATGAAAGAATATGCAAGGCTGGTAGATTTAGTGAGGATGAGGTAAAATAATTTCCACAGGGGTGAGTTTTATAGTGGCCCCTATAAATGGAATCAGTCCTAACTTTATTTTACTTTACTATCCAGGAAAAGAAACAAAAGATCCATCTTTATGTATTGAAGTACTTTTTAGTTTGCAATAGTTAATAATATAACAATAACTTACATATCCTTGTATCAGGCAAGGTTTTTCTTCCAGCAATTGATATCAGGACTTAAGTACTGTCATACAATGGTAAGTACAAATTGGCACTCTTCAGCTAATATGTTGTATTTCTCCTTGGTTGACGTTTAGTAAGGAACAGAGTCATTCTTGCAGCATATATGTCATAGAGATCTTAAGCTTGAAAATTCACTCTTAGATGGTGGCACAACACCACGTGTGAAAATTTGCGATTTCGGATACTCAAAGGTATTTCGCAGAACCTATGATATTTCAATTTTGGGGTTTCAGTTAGATGGGATGTTGAATAATGGTTCGCTTTGTTGTTGTAGTCGCTACTTCAATCTCAACCAAAATCTGTTGTGGGAACACCAAATTATATTGCACCTGAAGTCCTATCTAGAAAAAAATATGATGGAAAGGTGAGTGCAAGCTCTTGTGTATTTCATGGACACTAGTGTTTGCTATTGCACACTTTGAGCTTCTTTTTTTTTTTATCTAATCTTAGACAATATATAGATTTCAGATGTCTGGTCTTGTGGAGTCACCTTATTTGTCATGATTGTTGGGGCATATCCCTTTGAAGATCCTCAGGATCCTATAAACTTTACAAAAACAATTCGGGTGAGTGGCATCAGAACCGTTTCACTTCTCCCCTCGTTTCTGAGAGTTGTAATGTTTTACATGTCTCGTGGCAGCGCATCCTTAGTGTACACTACTCAATCCCACAGAATGTTGCAGTTTCCCGGGAATGTAGACACCTGTTTTCTAAAATATTTGTGGCAAACCCAGAAAAGGTAATGTAGGTACAGATTATTTTTGGTGATGAGCTGCTAGATGTTTACAATTATTAATCAGATGATATTGTTTCCACATTCTATCAAAGAGACAAACAAAATTATTAGTTATCTTGTAACCAGTTATTGTTATGCTGGTATATGAGCAGTTACTTTCAGTTTTCCTTTCCCTTTCTCATAAGGGTCATACTGCATCTATCTTTGCAGAGAATAACAATCCCTGAAATCAAGAGCCATCCTTGGTTTGTAAAGAACTTACCTATGGAAATGATGGAAGGAGGAAGTTGGGAAAGCAATGAGATGAACAATCCAGTCCAGAGTGTTGAAGAAGTTCAGTCTATAATACAAGAGGCAAGAAGACCTTTAAAAGTCCGAACTGTCAGTAGGCATCTCACTGGTAGCAGCATGGCTCTTGATGAAGCTGATGTTGGAGGTCCTGAAACAAGTGGTGATTTTGTGTGCTAA

>FvSnRK2.9

ATGGATCGGGCGGCGGTGACGGTGGGGCCGGCGATGGACATGCCGATCATGCACGACAGCGACAGGTACGATTTCGTGAGGGACATCGGGTCCGGGAACTTCGGGGTGGCGAGGCTGATGACGGACAAGCAGACCAAGGAGCTGGTTGCCGTCAAGTATATCGAGCGCGGCAACAAGGTCAGTTTCGGTATTTCAAATCTGGAGCAAGGAGGAGAGGGGAGAGTTTGTAAATTATGTAACCGATTTTTTTTGGGAGCATCCATTTATGGAAACCACGGTTTGGGCTTTTCGGCTTTGGGTGATGTTGTCATGCTTTTTTGGGGTTTCTATTTTTAGCATTTTGATCGGTTTTGGGGTTCTTTGCCGTTTTTGTCTTTATTATGACGCATTTATATCGTAATGGTGAGGGATTTAACTGTTTGGGGAAATTATATTCCGTATTCTGATTAACAGGGATTAATTTCTGATTAGCATATTGAGTTAAAATTGTTATATGATTAGCTGAACATGCTTGATGTGTAGTGCATCACTAAAAATGGAATTGCTTTTTATTGATTGGTAAAGAGGTTTGTGTGAGATTTTTTGGAGCAGTTTGGTTCTGACTGTGGTGCTGGGTGTTGCAGATTGATGAAAATGTTCAAAGAGAGATTATTAATCATAGGTCGTTGAGGCACCCCAACATTGTTAGGTTCAAAGAGGTTAGTGTGCTTTTCATTGATGGTAAGATTTCAGTTTTCGTTAAGGTAGGGTAATTGGCTGCTAATGTGTACTTTGAATTTGACTATCTATACAGGTTATTCTGACTCCTACTCATCTGGCCATTGTAATGGAATATGCTTCTGGAGGAGAGCTTTTCGAGCGGATATGCAATTCAGGGCGCTTCAGTGAGGATGAGGTCTTTATTCGATTTATGTCTACAATTGCAATCTTTTCTTTTTACCTTTGCTGATATGAGTGGTTACTTTTTTTTGTTTAGGCTCGCTTCTTTTTCCAACAACTTATATCTGGGGTCAGTTACTGTCATGCAATGGTATGTAAAGTTTTCTTTTTGGCTTTTCTAGCATTTATGTGTTTTCCATGGGGTTTGGAGTGGAAACTTACTTGGATTTGTATTTTTTCCTTGATGAAGCAAGTATGTCACCGGGACTTGAAGTTGGAAAACACTTTGTTGGATGGAAGTCCTGCTCCTCGCTTGAAAATATGTGATTTTGGGTACTCAAAAGTATTATTCTTTCTTTTCTATTTGTTAATCTCTCTGTTACTTATCTGGTGTATACGTTGTCTTGACATTTTTAATAGATGAAAAGAGAGCTCAGAGTTAGTCAAGATGGACTTCTGACAGAACACTTGTTTCTATATCAGTTGCTTATGAATCACTTAAGCTAATCCAAGCCCCTCCACCAGTGTTTTTTCTGTTAGAAACTTAAGGCTCACCTGATATCTCAAGCCCCTCTGTTCATGAATTTTGATGGGATCCTATTTGGCTAATCATTTTTCTTTTCATGAGACGTATGGAACTGCCACAGCAGCCATATATATGATCATAATTAAGTTAGGCAATTATACTCGATCATCTGTATGCATTTATTGGATCGTTATCTTTTATGCATCTGATATTTGTTATTTTGTCAGTCTTCAGTGCTTCATTCACAACCAAAATCTACAGTTGGAACTCCTGCATACATTGCTCCAGAAGTACTATTGAGGCAAGAGTATGATGGAAAGGTATGATTATTTTTTTAGTTTATTTTAGTTTATTTGTTTTACTGGAATTGCCTTGAGAATTTTATTTGATTATTTGCTAGCTTTCTTTCTGGACTAGTCCATGAAGCTGAATTTTAATAGTAATGTGGATTCAATTTTTCCTTGCTGTCCCATTTAGACTTCTTTGAAAGTTTGAAGAACTGCAATCTACAGAAAGCTCTATATTTCAGACTTCAAATAACACTCTAGCATGTGACAAATTGAAGGTTAAGGTCTTTTTTTTATTAGATTTATTGTTGTTTAGTTACTAATATGCATGGAAATCCGAAAGACTGGTCACTTTTGGTGGATCGTACAAACTTAAACTGCAAATGAGTGTCCGACTGTTAGAGAAAACTCAATTATTGCTTTGCCTTAGGTCTTGTCAATATGGAATCCTGTGTTAGGGCATATACATGTAGTCCTCGTTTTGACCTTTTCCATGAAGCAGCAGCTCTCAGAATTGAAGACAGTGATCTCCTCTGATAGGCAAAATCTTTTTACTGTACCATTGTACTTGACAATTAAATATCCTTAATGTTATTAAGATGTTGATGTTTTCATTCAATAACGACATTATATGATTCTCCCTACATTTTCATCTGTGCTCCTGCATCGGGAAGTCATGTCATAGCTATTGACTACATTTTAGGTACAGAATTAATGCAATGTGCATCTGCTAATTTTGTGTGTGTATATACAGTTTATGTATTACTCGTAAAAAAAAAAAAAAAAAAAAAAATACAGTTTCTGTATTAACTTGGAGCTTAATTACACTTTTATAAGACTCCTTATCTTGCATGTCATCTTTCTTGAAAACATTCATTGTCTTTTCTGTCATATGTAAAGATGAAGTTTTATATGAGTTCAACTAATGTAAGTTTCTATCGTGTTAATTGTCAGACTGCAGATGTATGGTCATGTGGGGTAACGTTATATGTGATGTTGATGGGATCATATCCTTTTGAGGATCCTGATGAACCAAAAGATTTCCGGAAGACTATACAAGTATGTCATTTGGCCTTATCACTACCTAAAGTTTCTTAATGTAAAATGTATGACTCAAGATATATCATCTACCGTCTCTTCGCAGAGAATTCTTAATGTCCAGTATGCAATTCCAGATTCTGTTCCATTATCTTGTGAATGTCTGGAACTGATATCAAGAATTTTCGTCTCAGACCCTACTGCGGTTAGTTTATTTTTCCTTCTGCTCTACATATTCAGATAACAGTTACTTTTCCTTCTATTACAATGTATATATGTCTAACAAAGTAAAGGGTGCATGTCTGGTTTTCAAAATTTTCCTGTTTGAAGCGATATGTTTCAGAGGTTCTGTCTGAATAAATATCTTCTCTGCATGCATGTGCCTTTTAATTGGAATTCATGCAATTTAGGAGTTAATTTTTTTTTTTTTTCTAATACTGCTGGTTGTCATGGAAAGAACAGCACGTAGTTTAAAATTTTGGGCTCTAATCATATGGAGTTTCAAATTTATCCCAAAGTCCAGGAAAGTTGCCATAACCGGCTTCAAGGGATATAAAACCTCTTTCTACTTGGAATCCGTTAAAGTAAACATGAGCAATGGGTGGATGATAAATTAATCTGAACGAGATCAGATAAGTTAGATTTGTCAAGTTAGATAGAATACAAGTATTACGGTACTATTGTATTTTCTCTAGTTCACTGTTTAGCACTTGATGCCAGCATCAGTGCTATTTTACTTGTTACAACATGAAAATACAGCAGAAATTTAGACACGTACTACTGATTGCAGAGATCTTGGAAGTTGTTGCTTTTGTTATGTTGGTTTGCGAGGTTCAGTTAGTAGAGACCTTACTATTGTTTATTTCTTCCACAATTTTTTCGTTCTTAATTGTGTCCATGTTTTATGCTTTGTATCAGAGAATTACAATTCCTGAAATAAAGAACCACCCATGGTTCTTGAAGAATCTCCCCGCAGATTTGATGGATGAGATGACAATGGGCAACCACTTCGAAGAGCCTGATCAACCGATGCAGAGCATTGATACAATCATGCAAATAATTGCTGAGGCCACCATACCAGCAGTTGGAATCCACAATCTTAGCCCATTCATGAATGACAGTTTTGACATGGACGATGATATGGATGACTTAGATTCAGAATCTGAACTAGATGTTGATAGCAGTGGGGAAATAGTGTATGCTATTTAA

>FvSnRK3.1

ATGGTGTTGAAGCTTGAAATGGATAATAAGTCTTATATATTGATGCAAAGGTACGAGTTAGGGAGACAACTGGGTAAAGGCACTTTTGCCAAGGTTTACTATGCAAGGAGCTTGATAACAAATCAGGCTGTGGCGATCAAGGTTGTTGACAAAGAGAAGATTATGAAGGTAGGGCTGATGGATCAGATAAAGCGTGAGATATCTGTTATGAGACTGGTTAGACATCCCAATATTATACACCTTTATGAGGTCTTGGCAACCAAAACTAAGATATACTTTGTCATTGAGTATGCTAAAGGTGGTGAGCTATTTAACAAGGTTGCTAAAGGAAAGCTGAAGGAGGATGTCGCACGGAAGTATTTCCAGCAGCTGATCAATGCTCTTGATTTCTGTCATAGCAGGGGAGTTTATCACCGGGATATTAAGCCAGAGAACTTGTTGCTAGATGAGAATGATAATTTGAAGATATCTGATTTTGGGTTAAGTGCCCTTGCAGAAAGCAAGCGCCAAGATGGTCTGCTTCATACCACTTGTGGTACTCCTGCCTATGTTGCTCCAGAAGTCATTAATCGGAAAGGCTATGATGGTGTGAAAGCTGATGTTTGGTCTTGTGGGGTGGTCTTGTATGTCTTATTGGCTGGTTATCTCCCGTTTCATGATTCAAATTTGATGGAGATGTACAGGAAAATCGGTAAAGCAGAGTTCAGATGCCCTAATTGGTTCTCACCAGAAGCACGTAGGCTATTGTGCAAGATGTTGGATCCAAATCCCAACACTAGAATTACCTTGGCGAAAGTTAGGGAAAGTTCTTGGTTCAGAAGAGGACCAAAATCCAAAGAGAAAGAGGTGGCTCCGGCGGCAACAGAAGCTTCCAGTCCCAGTGAGAATGAGAATAATTTGGCTGTTGAGGCAAACCAAGAGTCAGGGAGACCTTCAAACTTGAATGCTTTTGATATTATATCCCTATCTGATGGGTTTGATCTGTCCGGCTTGTTCGAGAAAAATTCTCTAAGTAGGGAAGCAAGATTCACTTCAAGAAAGCCTGCCACAGTCATCATCTCCAAGCTAGAAGAAATGGCCAAGTATCTGAAGCTAAAAGTGAATAAGAAGGATCATGGATTGTTGAAAATGGATAGACTGCAGGAAGGCAGAAAGGGTTTTTTGTCCATTGATGCAGAGATATTTGAGATCACTCCGAATTTTCATTTAGTAGAGGTCAAGAAATCAAATGGAGATACAATGGAATACCAGCAGATGTTGGAAGACATAAGGCCTGCTCTGCGAGATATTGTCTGGGTTTGGCAAGGGGAGCAAGAACAGACATCGCAGGTGCAGCAGCTACAAGAACAAGAAGAAGTGCAACAGCAACAACAAGAACAATTGCCTCAGAATCCGCCACAACTATAA

>FvSnRK3.2

ATGGAAAACAAAGGGAGTGTGCTGATGCAGCGATACGAACTGGGGAGGTTATTAGGCCAAGGAACCTTTGCCAAGGTCTACCATGCTAGGAACCTCAAAACCAACATGAGTGTGGCCATAAAGATAATTGACAAAGAGAGGATCTTGAAGGTCGGGATGATTGATCAGATCAAGCGTGAAATTTCTGTGATGCGGTTGATTAGACATCCAAATGTGGTGGAGCTTTATGAGGTGATGGCCAGCAAAACCAAGATTTACTTTGTCATGGAGTATGTCAAAGGTGGTGAGCTCTTCGACAAGGTCTCCAAAGGCAAGCTAAAGGAGGATGTTGCTAGGAAGTACTTTCAACAGCTCGTCAGTGCTGTTGACTACTGCCATAGTAGAGGTGTATACCATCGTGATCTGAAACCAGAAAACCTACTTTTGGATGAGAATGGAAATCTAAAGGTTTCAGATTTTGGACTGAGTGCCCTTGGTGAATCCAAGCGCCAAGATGGATTGCTTCATACAACCTGTGGGACTCCTGCATATGTTGCCCCAGAAGTAATAAACAGGAAAGGCTATGATGGTGCCAAAGCTGACATTTGGTCATGTGGGGTGGTATTGTTTGTTCTATTGGCTGGCTATCTTCCATTTCATGATGCAAATCTGATGGAGTTGTATAGGAAGATTGGTAAGGGGGAATTCAAATTCCCTAACTGGTTTACTCCTGAAGTACGCAGGTTGCTGTCAAAGATATTTGACCCAAATCCAAATACTCGGATATCTTTGTCTAAAGTAATGCAATCTTCTTGGTTCCGAAAGGGGCTGGTCCAGAAACCTGCAATTGTTGAATTACCAGTGAAAGAGCTTGCCCCTCTGGATGCTGATTCCATTTTTGGACCTGGTGAAGATAACAATTCTGTGACAGAGGCAAAGCAAGAATTAGTGCAGAAGCCGTCTAACTTGAACGCTTTCGATATCATCTCCTACTCCGCTGGCTTTGATTTGTCTGGACTGTTTGAAGAGGCTGAACAGAAAAAAGAAGTGCGGTTTACATCCAACAAAACTGCTTCAACCATCATCAGCAAGCTGGAAGACATAGCCAAGCGTCTGAAACTCAAAATCAAGAAGAAAGATGGAGGGTTGTTAAGAATGGAAGGGTCCACGGAAGGCAGGAAAGGGGTTTTGGGCATTGAAACTGAGATCTTCGAGATCACCCCGTCTTTTCACTTGGTGGAGGTGAAGAAGTCTAGTGGAGATACATTAGAGTATCAGAAGGTCATGAAAAAGGAGGTAAGACCAGGTCTCAAGGACATTATTTGGACTTGGCAAGGGGAGCAACAGCCGCAGCAACAAGAGCCACCACAACCAGAGCAGCAAGAGCAACAACCTTTGACGCTCCCAGTTCAAGTAGCCTCTCCCCAGGAGGCATAA

>FvSnRK3.3

ATGGCTGAGCTGCGCCCCCAAAACGGCGCCGTCTCTACCCCCACCACAACCTTCACCTCGAACACCTCCAAGACCAAGAACAACCCTCTCCTCCTCGGCCGCTTCGAAATCGGGAAGCTCCTCGGCCACGGCACCTTCGCCAAGGTGTACCACGCCCGCAACATCAAGACCGACCAAGGCGTCGCCATCAAGGTCATCGACAAAGAAAAGATCCTCAAAGGCGGCCTCATCGCCCACATCAAGCGCGAGATCTCCATCCTCCGCCGCGTCCGCCACCCCAACATCGTCCAGCTCTTCGAGGTCATGGCCACCAAAGCCAAGATCTACTTCGTCATGGAGTACGTCCGCGGCGGCGAGCTCTTCAACAAGGTCGCCAAGGGCCGCCTGAAAGAAGAAGTCGCTAGAAAATACTTCCAGCAGCTCATCTCCGCCGTCGGGTTCTGCCACGCCAGAGGCGTCTACCACCGCGACTTGAAGCCGGAGAATTTACTCCTCGACGAGAATGGTGATCTGAAAGTCTCCGATTTCGGGCTCAGTGCGGTTTCGGATGAAATCCGGCAGGACGGGCTGTTCCACACGTTTTGCGGCACCCCGGCGTACGTGGCGCCGGAGGTGCTGGGCCGGAAGGGCTACGACGCCGCGAAGGTGGATATATGGTCTTGTGGAATTGTTTTGTTTGTGCTCATGGCGGGGTACTTGCCTTTTCATGACCACAATGTTATGGCCATGTATAAGAAGATTTATAAGGGAGAGTTTCGGTGTCCGAGATGGTTCAGCTCCGAGCTTGTGAAGTTGCTGACTCGGCTTTTGGATACGAATCCGAATACGAGGATTACCATTGCGGAGGTGATGGAGAATCGGTGGTTTAAGAAGGGGTTTAAGCACATTAAGTTTTATATAGACCATGATGACAGGTTGTGTAATGTTCATGAGGATGATGGGGATGATAGTGATGCTAGTTCGGTGATGTCTGATATGTCAGAATCCGAGGCTGAATTCGAGACCAGGAGGAAGCTTACGACTTTGCCGAGACCGGCCAGTTTGAATGCGTTTGATATCATTTCGTTTTCGCCCGGGTTTGATTTGTCCGGCTTGTTTGAGGAGCGCGGGGAGGAGGCTAGGTTTGTGTCGGGTGCTCCGGTTGATAAGATTATATCAAAGTTGGAGGAGATTGCCAAAGTGGTGAGCTTTTCGGTGAGGAAGAAGGATTGCAGGGTGAGTTTGGAAGGGTCTAGGGAGGGTGTGAAGGGGCCATTGACGATTGCGGCTGAGATATTCGAGTTGACGCCCTCGTTGGTGGTGCTTGAAGTGAAGAAGAAAGCAGGGGACAAAGTAGAGTATGATCAATTTTGTAATACGGAGTTGAGACCGGGGTTGCAGAATTTGATGATTGAAGAATCTGCTGGAGGTTCTCTTGCTTCGGGAGGTTCGGTTGTTTCAGAAGGTTCTCTTGCTTCAGGTGGTTCAGTTGTTTCAGTAGGTTCTCCGTCGTCAGTTCATCATCTACCCTCTGATACTGAATAA

>FvSnRK3.4

ATGAATCAACCAAAAATCAAGCGTAGAGTGGGTAAATATGAGGTGGGAAGGACAATTGGCGAGGGAACATTTGCAAAAGTCAAGTTTGCTAGAAATTCTGAGACTGGAGAACCTGTGGCTCTTAAGATTCTTGACAAGGAGAAGGTTCTCAAACACAAAATGGCTGAACAGGTCTGTATGAATTGTTCTTGCTTGCAACTCACACACACACACACACACTCTCTCTCACCTGTGGATGGTTTCTTTTTATAATTGGGATGATAAATGCAATACATAACTATGTCAGAACTTGTTCTTATTAGTGCTTGGTGGGCTAATAACATTTTTCAATTACCTTCCCAAAAAGGTAGAAGGTTTGCAAATATTGTTTTGGCACATCAAATTTTGTAACTTCATTCTCAGCTCAATAATATATNNNNNNNNNNNNNNNNNNNNAAAATTCAAAAGAGCTGTTCAAATGTTCTTTTGGCTGAGAAAATTTGCTGACTTGATTCAGGCCCCTAGAGTGATGTCCAATTTTAGTTCAATGGTATCTGTGTACCAAGAAAGTGATAGATGGTTCAAATTTTTTTTTATTTGGTCGTTTAACTGTTATATTTAAGTCGTTTTACTTTTATATTTAAATACCTTTGGAGCCATCTGTCTCTGCAAAGCGATTTATATTGATCAGAACAGAATTAAATTATCATGCAAGTATATAATATAAGATCAGTTGGTTTAAATCAAGGTCTGTTCTATGTGTTTTTACAGATCAAGCGGGAAATAGCAACAATGAAGCTGATTAAGCATCCAAATGTTGTTCAGTTGTATGAGGTCTCTCTCTCTCTCTCTTAGTGCATTCGTTTGCGCTTAATTACAATCCCACAAAACACAATATCCACCTAGCAGGTTAGCTGAAGTATCAATATTCTATTGACAGATATGACCTATTATTTTTGTTCCCTTCATTTTGGCCCAATCCTGATCTTGCACTATAATCAAAATATAAAACAGACAGAAGGTTGGTATACATGAATCTACTATTAAAAGTAATACGTAGGTGATTGTATGAATGCGCAAGATGATTTAATGGGCTGGGGAAAGACAACTTCACATAGAAATCAGTAAAATTAGAACAACAGTTTGGTTCCTTGAGTTGATTTCCTTATACTGCATTCTCTTTATGTATAAATCTATGCATTTTCAGGTCATGGGGAGCAAGACGAAGATATTTATAGTGATGGAGTTTGTTACTGGGGGAGAGCTCTTTGATAAAATTGTGAGTGCCTAAAGTCTCTGCCAGTCAGTATTATAACCTTATCTGCTTTGTTAGTCAAATAACACATTATTATTCAATTACAATTGCTGTTTTCATATTCCAATTCATCATTGATTATCCTTAGTTATGATGTTATGCTGCTCAGTCATTTCTGTACTTGGATGTCTTATTCTTGTTAGATTCAAGCTCTTCTGATTATTTGGTAGATCTTGCATATCTTCTCACAAAAACTTATTTTGCTTTGAAGGTAAACAATGGTCGGATGAGAGAGGATGAAGCACGTAGATATTTCCAACAGCTTATTAATGCAGTTGATTACTGCCATAGCAGAGGTGTCTATCACAGAGACCTGAAGGTATTTGACCTTTGCAGAAATTGATTTAATAAAATGATTATTGGAAATTGATGCTAAATAATCACTCAATTTCGTTTCATTTCGTGCAGCCAGAAAATTTGCTATTGGATGCCTATGGGAACCTTAAAGTTTCTGATTTTGGATTGAGCGCACTATCTCAACAAGTCAGGGTATAAATAATAGTATTAATTGCATTCCTCTTGTTCTCACCTCATCTACTACCCATTTGTTCTGATAGATGTTCACCTATGTTTTGAGGAAGTTCAACATACATTTGCAAATATTTTATAGTCACATCTTCACTGTTTCTTTGAAATTTTATAAGAATTTCTGGAATTCAGAGGGATTTGTATGGCCTTTAGTACTGCAAGCATTTAATCCTTCTGTCTCTTAGTATAGACTTTCATCAATGGCATACACTTGAAATTTTATAAGAACTTCTGGAGTTCAGATGGGTTTGTTTGCGAAGAGTAGGGCCTTTAGTTTACTCTTGCAAGCATTTAATCCTTCTGTCTCTTAATGTAGAATTTCATCAGTGGCATAAGCTTCGGTGTACCTGTTATTGTAATTAGCTTGCCTTTTTTCCATATTGCATCATGCACATTGGGTCCAACATTTTTCTCTTACTCTTAAGATTGAATTGGCAGGATGATGGCTTACTTCACACTACCTGTGGAACTCCAAATTACGTTGCTCCTGAGGTGTGTTGTTCATGCCAAACTTCGTCTATGCATATGTATTTTTCTACTCTATTGGTCCCTAATCCTAACTCAACCGTAATTGTGTATAGGTCCTTAATGATAGAGGCTATGATGGAGCAACTGCGGACTTGTGGTCATGTGGAGTGATACTCTTTGTATTACTTGCAGGTTACTTGCCGTTTGATGATTCCAATCTTATTAACCTCTATAGAAAGGTGAGCATTTGACTTATTCTACTTGAACTATATGTCATTTATTTATCCGCATTTTTACATTTTTTCTCTCTATCTGCAGATCTCAGCAGGTGAATTTACTTGTCCCCCTTGGCTGTCGTTTGGTGCCATGAAACTAATAGCTCGAATTCTGGATCCCAACCCTATGACAGTAAGCATTCATGCATTTTCTATGATAAGCTAGCGGCTTCAATACCAACTCTTATATCTGAAATCTGATGTATACTATTGCTCCACTGTCTATAATACGTCTCTCTTACAATGATCGATAATCATGTGAAAACCATATTCATGCTAAAACCCAACGGTTCTACATGCTGATGATTGAAATGTATTGGAAGTGACTGCAAGGTTTTTGATAGCATACTAGTATTTGTTATTATATACAACATATATCAGTATGTAACTTTATTAGTGTGCTGGTGGTACACAAAATTCGATGGCAAACTATGACTTCATTCTTGCTTGAAGAATCAATGTTGCAACTTTTCATTGTTGTAGTTTGGGTATTTCCCTTTACCTAAGGGGGTTTTTCGATTGCATGTCATAATTGATTGCATGTCATAATTGTTCCATGTCTTTCAGTGTGTACATTATTTCATCTTCTTTTTTTAGAGCTCATGAAGTATGAGATAATTATAGAAATCGACACATTAAGACTTGAATTTGATGATAATATATTTTCTAGTTATGAAGTGAAAGACAAGAAAGGAAAAGAAATGATTCCTTTTTCTTGTAATTCATCCCTATTTATTTTTCTTTTGGCTTGATGTTCCATGATAGCTTCCTCCTTTGTGGAGGTTTAATTTTAATGTATTTTTTTTCTTTTGTAAATATTAAGATATGTAAGGCATTTCTCAGAGATTTACTTAGCTTCATATCCATAGACTGTTCGAGTACTTTGAGCATGCTATATCAAATCTTTTTGAATCATGGGCTGAAAATTTTCATATACCTCATTGCTTTCTTTCCTCAACTACAACAGAGCATACATTACCGGGCTCAGTAATCTAATATATAGTGAACTCACTGAACTGAATAGAATTCAAACATTAACATCCATAACTGAAAATTCTATGTACCAAATGTTAGTGCGTGGAAATTCTTAAGTGTCCTCTCACCACCAATTCATCATTTAGTAACAATAAATTATTTTCTTCTGTTCTATGGCAGCGTATCACTATTTGTGAGATTCTGGAAGATGAATGGTTCAAGAAAGATTACAAGTCACTTATGTTTGAGGAGAAAGAAGATACAAACTTGGATGATGTAGAAGCTGTTTTCAAGGATTCAGAAGTATGCTGAAATGCTTATGTCCATAGGATTGGAATTTATTACATCTTTGGGTTCTAAAGAATGTTAAGTTATTCATGAATTGCTGATATTTCGGTCTCTTGACAGGAGCACCATGTAACAGAGAAGAAGGAAGAACAACCAACAGCTATGAATGCTTTTGAGTTAATTTCAATGTCAAAGGGGCTGAACCTTGGGAATTTGTTTGATGTAGAACAGGTAAGATTCAAGTAGGCTTCTGGCTTTCATTCGGCACATCCATATTCCCATTACAATTTACAATGTCCAAAAGCTGAAATTTCAATTAGTCTTGGCTCAGCATTCTATGTTTTACATGAGAGAAAAATGGTCTGATGAATGTCTGCATCACAGAAGATGATCCCAGTACGTGAAAACTTTTGTTGTGTTTATTTTCTTTTTCGAAAGACTCTTTATAACCTTTTTACTACATTCTAAAAAAAAACCTTATTTACTCCTTTGTTGATTTTTTTTGTTTGTTTTGGTAACCTATTACCTAGTCGTTTTTTGTTTATTAATGTTATTTTTTTTGTTTTCTGTTTTCTGTTTTCATTTTATTTTTAATTTGCACACAATTTGCAGGGTTTTAAGAGAGAAACAAGATTCACATCTAGATGCCCTGCAAACGAGATAATTCATAAAATTGAGGAAGCTGCAAAGCCCCTTGGTTTTGACGTACAGAAGAAAAATTACAAGGTTAGAACTCATACAATTGACATCCAGGTTATGAATCCTGGAGTTTAGACTTCAGATTTTGAAATCTGTGACTTATTTTCTCAGTTGAGGCTAGAAAACATGAAAGCTGGGAGAAAGGGAAACCTTAATGTTGCAACAGAGGTACACGATGATGCGATTAAACTTCAATAATCACTATCAATCGTACCTTTTACTAACCCTTGTCATGGTCAGATATTTCAAGTTGCACCTTCTCTTCATATGGTTGAGGTGAGAAAAGCCAAAGGGGATACGTTGGAGTTCCACAAGGTATCCTCTCTTCTCTTGATTTTCATTGTATACATATATACGTTGGTGCTTGTTGTATAATCGTGACATTACATGGTTACTTGATGTTAGTTTTTCCTTGTTCTTGTGCCTTTTTTAAGGATTTTTTTTTCTGTACTTGTAATTTTATTTTGCATATTTCATTATTTACCGCATATTTCATTATTTACCCTGTGACTTTATGGTTCTACTTATTTACGCGTATATCTGACTGAGGTCAAGTTTTGTACAAGTTTTTGACTGTTTAATTTGGTTCACCTCATGGTGATTAAAACTATCATATCGGTGATAGTACTATAAAACCAGACATTCAACTGATTTTTTTTTTTCCAATAATAGTTTGACCACTAGTGGTAGAAAGTGATTCTCAAAAAANAAAAAAAAAACAAACAAACAAACAAAGTTGTAGAAAGTGAAATAACTTGGTATTTCCTTGCAGTTTTATAAGAACCTCTCAACCTGCTTGGAGGACGTTGTTTGGAAGACTGAGGAGGACATGCTAGAATGA

>FvSnRK3.5

ATGATTGGCTTCCGGTTAAGGCAGTACAAAAAATCATATGGTAAGCCACCACTCCATCCTTCCTTCTTTGGGCCCCACACGTTCCCAACACGATAATCATAATCTTCTTCTATATTCTCCTCCTTCTTCTTCTTCTTTCATATCCTGCCACTCTGTTCTCTCTGCCACCCAAAATTTGCCCGAACGTTAAAGAAACACAGACCCAGTAGAACAAAGATCGAAGCTTGAGCCGCCGGAGGTCGGACGCAGTTGGGAAACTTGAGCCGTGTGATCTTGATCATAGGAGGATGAGTGCTCCCAAGTCGCCGAGGATGAGGACCCGGGTCGGCAAGTACGAGCTGGGTAAGACTCTCGGGGAGGGTACCTTTGCCAAGGTCAAGTTCGCCAAGAACACCGAAACAGGGCAGTGCGTGGCCATCAAAATCCTTGATCGTGAGCAAGTCCTCAAGCACAAGATGGTTGAGCATGTATGTAACTATCTTTATTATTAGCTTTATACATCATGTTCATTATGAAATGTTGGATTATTGATTGATATATATGGTGATCATCAGTAATCATGTTAGTTCTTCGGCAGTGTATGAACCCACCACCTATTAGATGATCATAGTCCTTTGTATTTGTTGTTAACTTTTATTTTTTATAACAAAAAATGCTCTTGACCTTAATTTCTCTGCGTTTGATATTGTTTAAAGTTTATACTATAGAGATTGGAGGGCAGATGGATTGAGTTGGTGGGTCAGGGAATGTTTGTTTTATATCAATAATTGGTGTTTGATATACTACAGGTGGGCCAGTATATGTGTTGATGTTGATTTTTTGTTGTTGTTGCGAATATATGAGTTGGTTGTAGATTTGTAATGTGAGGCCAGAATGTAGATATTGATGTCTGATTTCTGTTTTGGTGCTTGAATTGGTAGATAAAAAGAGAGATATCGACGATGAAGCTGATCAAACACCCGAATGTGACGCAAATGTTTGAGGTACCATATGAAAATGATATGCAGTCGATTGCAGGAAAGGATTTCTGGGAATTCTGAGTGATGTTTTTTGTGTTTGTGCATAGGTTATGGCAAGCAAAACTAAGATCTACATTGTTCTCGAGTTTGTTGATGGGGGTGAGCTCTTCGACGAAATAGTAAGCAATTTCTGTTTGACCCTACATGATTGGGTTGCACAAAACTGTTTCTTTGATTTTTTAGGACACAAGTCTTATGTTTGATCCAGAAAGCATTCTCCTTATTGCATTTCTGTTTGCATAGGCCAAAAATGGGAGACTGAAAGAGGACAATGCCAGGAGATACTTCCAGCAGCTCATTAATGCCGTGGATTACTGTCATAGTAGAGGCGTGTACCATAGGGATTTAAAGGTTTGCAGACTCTGCAACTTTATTCCTGAATTTATTGGTTACTTGGTGTGTTCAGGGTTGAAATTTTAGTTTCTAGAGTAATAATTTGATTAACTTTAGACTACATCTTTTGCAGCCAGAGAATCTTCTCTTAGATTCATTCGGTGTCCTTAAAATTTCAGATTTTGGATTGAGTACATTTGAACAACAAGTGCGGGTGAGTAATTTCTTGTGTTGGTTCTATATTTTAGTCATATCCAATTGTGAGATGTAGGTTACTCAAATTAGAAAACAAGAACTTCCCCTTCCTTTGAGGAGTATCTGTGATGTTGTTGTTGATTGGAAGTTTTAATTAAGTCATGTTCCATTGACCATGTATCTTCTCCTTTACTTGCCTTTGATTTTGCCCAATTACAAGACCATGGATATTATGAAATGCTCAAGAACACGCTATATATTTTTTCATTGCAGGAAGATGGGCTGCTTCATACTGCCTGTGGGACTCCAAATTATGTTGCTCCTGAGGTATATGTTTCTGACTTCATTAAACTAGTTAATTGCTTTTTAGGTTTTATCTAACTGTTTCACAAATTTGGTATGTGTGTATCAGGTTCTCAATAATAAAGGATACGAGGGTAAATCATCTGATGTTTGGTCTTGCGGGGTGATCCTTTTTGTACTTATGGCTGGTTACCTGCCTTTTGATGAACCAAATCTAATAGCTTTGTACCGAAAAGTATGTATAACCAAGCTTTAGCTTCTGCTGTTTGAAAGAAAGGAATCTCATATAACATAACAGTGCATGCTACTGTGAATTTCAGATATGCAAAGCTGAATTTTCATGTCCAGCATGGTTCTCATCTGGTGCAAAGAAACTGATACACCGTATACTTGATCCAAACCCTGCTACAGTGAGTTTCAAAACTCAGTTTCTGTGCCAATATTATTTATCGAATCCATGTGATCTTCACATATGTTCTGAAAAAAGAAAAAAAGAAAAAAAATTATATCATTGTTAATAAGCTGTGGGCATAGAAACTCTGTAATTTGGTGCATATATCTCATGCCAGGTTTGATCCTGTATTATAGAAGAATAGCTTGATAGGTGCTCTTTTTTCATTTTATGCAGAGGATGACAATTCCTGAGATATTAGAAAATGATTGGTTTAAGAAAGATTACAAGCCAGCACAATTTAAAGAAGAAGATAACATAAATCTTGATGATGTGGATGCTGTTTTCAACAATTCGAAGGTAAGACTTTTAGTCAGATCTCTTTATTTATTTCTCTCTAACATGAAGTCTGTGCTTCTCTTTATTCAGAAGATCGTTTTAGAAAAATTTGATTGTCTTTTGAGATTTCTCATTGCTGAATCAGTTTTTCTCTTTTTTTGTTTGGTTGTAGGAAAATTTTGTAACAGAAAGGAGAGAAAAACCTACATCAATGAATGCTTTTGAGCTTATTTCTCGGTCACAGAGTTTCAATTTAGAAAATTTATTTGAGAAGCAGATGGTAAGTGCCAAGTAGCATACAATGTTCCTAATTTCTTTGTAGCGCCCAAAATTATTCTGATAACAATTGCTGCTTTCTGGTAATTTGGAATTCTTTAGGGTCTTGTGAAACGAGAAACCCGTTTTACTTCTCAACGCCCTGCAAATGAAATCATGAGTAAGATTGAGGAAACTGCAAAGCCTTTGGGCTTTAATATTCGCAAGAAAGACTACAAGGTAATGAGTTTTACTCTCTCTGATGTTACACTGCATATGACTTAATGCAAGTAACTTCTGAAAAAAAATGCTGCATATCTTTTCTAATCATAAGCTTTTTCAGATGAAGTTGCAAGGTGATAAGCATGGAAGGAAGGGTCACCTGTCTGTAGCCACTGAGGTACAGACTTGTTAGATAACGTGTATACTTGTACTTGTATATTCTGGAAAAGAAGTTCACAATCAAATTTAGGAAGAAGTGAACATTTAGATAAAATGATGTTTACTTGTGTAAGTGTAAAATTTGGTTATAAACTTATAATCTGTCGAATTTTATCTTGTTACATAAGGTGTTCGAGGTGGCTCCCTCCGTGCACATGGTAGAACTCAGGAAAACTGGCGGTGACACACTAGAGTTTCACAAGGCAAGTCATCACTGA

>FvSnRK3.6

ATGGCGGCGAGGGCGGCGGGCGTTGGGAGTCGGACTCGGGTCGGGAGGTACGATCTGGGTCGGACCCTAGGGGAGGGCAATTTCGCCAAGGTCAAATTTGCGAGGAACGTCGAGACTGGTGAGAATTTCGCTATTAAGATTCTGGATAAAGAGAAGGTGCTCAAGCACAAGATGATCGGCCAGGTATGTTTTTTCATTTAATTTTATTTAAAGTTTAAAATTTTCGCTGAATTTTCTGGAAAAATATGTTCTTGGCTAATTGGATGTTTAAAATTAAGATGAATGTTTAGTCTTTTTTTATTATTAATTGTGTGATTAATGTGAGCTAATTGCAAAATGTGGGGAAGGAAAAAGTCAAACTTTGAATGAATGATTGACGTGCATGATTGTTTGTCATTTTTTAATCTGGGTTATTTTACTGTTTGATCATAGCCTTTTTTTGACTTTAACAGCTCTGGTGTCATAATCATAAGATTAAGAGCCTTTTTTGACTTTGAAGAATTTAGTAACTGAAAGAGTTATATACTGTTCATTATCATGTTATAGTGATGACTTGGCCTCCAAGTTTCCAATGATTTCAATCTTAAAGCGTATTCTTGATAGAAATGGATCACCTGCAATTTTGCACGCATTGATAGAAATGGATGCCGGCGATTGTTGCACGGATTAACGATCCCGGCATATACTGTTTGTATGATGTCGGAGGAAAACCAAACTACACTTGACTACTTGACTTGTAATTGAATTACATTTGCTATTTCGGCCAAATAAAGAGGAAAGATTTATACTGAGCTTGTTGGTAGTTCTGTTGATAGTAAGCTCAGCGAAAGAGAAACAAGCTAATCAAATAGTATTACTTGCCATGTTTGATCCTCAGCTAACCCTACGGTGCTGGTTGGTATCTGATGTACAATTATTTGTTAAGACAGGAGCCTTAATCTGATTTTAAAACTGTGATCTTTATGAAGAACTATGTCTTGATTAATTGAAGATATTGAGAGATTGTTTCTTATGTTAATCCTCTTTGCAGATCAAACGAGAAATATCAACGATGAAGTTAATTAGACATCCCAATGTAATCCGTATGTATGAGGTTAGACATACTCTTATCATCCTTTTCCATCTGGAGTAATAAAATTTGTTTTACTATGACTTTCCTGTTTCTAGCGTTCATCTCTCTCTCTGTCTCTCTCTCTCTCTCTCCTGTTCTGTGTTGTTTGTAAGTGATTTCGTGCTGTATATTGCTGTAGGTGATGGCTAGCAAGACAAAAATATACATCGTTTTGGAGTTTGTGACTGGTGGGGAACTATTTGACAAAATTGTAAGATTCACTAGTTCAACTGTCATGCTGGACTTTCTATCTTAAATTTATGGTCAAAAATTAGAATTTCTGATATTTTCTTACTCCAGGCAAGTAAAGGAAGATTGAAAGAAGACGAAGCAAGGAAGTATTTTCAGCAGCTTATAAATGCAGTGGATTACTGTCATAGCAGAGGTGTTTTCCATAGAGACCTGAAGGTGTGTTCTTATTTTCCCACTTTTTAAAAACATACTCCAATTTATTCATTTATGATTAGTTCGAACTTATAATCTGTAAGAGACTTCCTGCTGTATGTGACACCCTAAGGCCACCAAACATGTTTACACTAGTTTTATTCATACAAAAGATAAAACTTCATTTCCTTAGAGAGAGTGCAAAAGGTAGCAATTATGTGAAGATAATAGTTCAGATGTCATGTGATAAATAAAGGTCTGTATATAAACAAAAAGAAACACACAAGTAACTGTATGATGAAAAGAAATACACACGACCTAGACAGTTAGGTGCTCGTCTTCAAAACTTCAAATCGTAGTTTTAAACTTCAGCCTTTTTTTGTTTAAAGTTCCTCACTTCATTAACTGTTGTTACGGTTGTAAATTGCAGCCAGAGAATTTGCTGCTGGATGTCAATGGAGTGCTTAAAGTTTCAGATTTTGGGCTCAGTGCGCTGCCTCAGCAAGTTCGAGTGAGTATCATAACTTCATGCCTAACAGAAATTGTGAACCCTATTAATCTGTTTGATGCTTGATACGAAGGATTGCTTTTGGAGAAATAGTTTTATCCCCTTTTTTACTTATGGTCTTAAAAATATATTGGAGTAGGTAGGTGCCGCTATACAATTATTTGTTAGAAAAGGTGTGACTGATTGTCTAATCCCTTTTCTTTAGGAAGATGGCTTACTTCACACAACATGTGGGACACCAAATTATGTTGCCCCTGAGGTATATTACATTAAGACATTGTTTTTCTTTTTCTTATTTCTTCCTCTTAGTGCCTCTTCTTTTCTAGGTCATCAACAACAAAGGGTATGATGGAGCGAAGGCAGATCTATGGTCTTGTGGCGTTATTCTTTATGTCTTGATGGCTGGCTATTTGCCTTTTGAAGATTCCAATCTCATGGCATTATATAAAAAGGTCATTTTCTATCTCTCTCTTCTTCTCCAACCTGTATATTCTCTATCTCCTAGCTTACTTTGTTTTCTTTTTCTTTTCATCTGTTTTACAAACCATATTTTTTTAATAAATATTTCATATCCTTGTGTTAAAACACATACACTCCTAGTTTAAGTGACTATCAGAATGACAGGATATTGTGTGTGAGATGGACTGTTTCCACGTATTCATAGATTTTGAAGTGTGCTTGTTTCTATATGTCTGACATGTTCTAAATCCTTTTTGAAAGCTTTATTTACAGACACTGTTCAATATTATGGCTCATACAAGATTGGATTTTCAGAATTGTCTTAATAATCATTTAGAAAACTTAAGAATACAGCATACCATAATATGGTCGTTCTAATCCTGCTATTTTTTACTTTATTTTTTATGAATAAATATAGATCTTCTTGTGATCACAGATATTCAAGGCTGAGTTCTCGTGTCCTCCATGGTTCTCCTCAAGTGCAAAGAAGCTAATTAAGAGAATCTTGGACCCTAACCCCTTGACAGTATGTCCTGGTCCCTAAACTTTTTACTTATAGCTCTTCGTTTTCAAAAATACAGTGTTTGTTATTTTTTCTTACTATTATTTTTATATGCAACTACTATACAAATTAATTGTATGAAACAGCGAATTACATTTGCTGAGGTCATCGAGAATGAGTGGTTCAAGAAAGGGTATAAACCACCTAGTTTTGAACAAGTTGATGTTAGTCTCGATGATGTGGATGCAATATTCAATGATCCTGGGGTAAGAGTGTTGCATTAGGTATTAAGGTTTATTCTGGTTTATAATGCTAGGAATAATTTCCAACTTTTCCTCTTCTGCACAGGATTCTCAGAACTTTGTAGTTGAGAAGCGAGAGGAACGACACGTGCCCGTTACCATGAATGCCTTTGAGCTTATCTCTACATCTCAGGGCCTGAATCTTAATAGTCTTTTTGAGAAACAGATGGTAATTTTGAAATTACTAAATTCTTATATGTATACCATTATGTAGATTGCTATTCTCATAAATTTAAAAATTTGTAAGCCTTAACTACTTATTCATTCATTGACTAGGAACTTGTTAAACGAGAAACAAGATTCACATCCAAACGTCCTGCTAATGAGATTATTTCTAAAATTGAGGAAGCTGCAGCACCTTTGGGTTTTGGTGTGAAGAAAAATAATTTTAAGGTACAATACACCTCACTCTCTCTCTCTCTCTCTCTCCCCATCTATATGTGATGTGAAAACCAAAGTATCTTTATTTAATCCAATGTACCTGTTATATCTCAGTTGAAGCTTCAAGGTGAAAAAACTGGTCGTAAAGGTCATCTATCTGTTGCAACAGAGGTACGTGTTCAGTTTAGGCATCCCAACTTCTTTTCAGAAGAATTTCCATGAGTAATGGATTATAAATTTTTTTTTTTTTTCCTTTTTGCAGATTTTTGAGGTGGCCCCTTCACTCTACATGGTTGAAGTCCGCAAGTCAGGGGGAGACACCCTGGAATTTCACAATGTATGTCACAATTGCTGAAACACTCATGGCATTTGATAACTATTGTTAATTCATATTTGAGATTGGCTTTACAAGTAAGGGACTCCAGGTATGCTGGTTGCTAGTTGTTGTGGGACAATGCTGGTGTTACCACCTTGTTTCATTTCATGACAGGCTCACGCTTAGAAATCGGTCTCCTAGTTTGTGGGTGTGATTTTAAACCAGAGGAACATGCATATTACACAGAAATTGGACTTGTTAAACTGATAATTATTTTTATCTTTATTAAATAAGAGACCAATACCACACTAAGCGGTGTCAACCTTTGAGATCCTGGAGAAGCTAGACTAGAGTTGTTGCTTACAGCCTTGCATGTTTTTTGCTTTTGTTTTTTGTTTTATTAGAAGGTACTTGTAGTATGTTGGTTACCATGATGCTTTGGGAATGATCCCTCCCACAGTTAAACTCCAACTATGACAAGCTTGCACAAATGTTGCTTCATTCTTCTCTTGTTAGCTTTTGTTATTTGCACATTTTAATTTTATTGGCTTTAATTTACAGTTCTATAAGAACCTTTCAACGGGGCTGAAGGATATTGTCTGGAAGTCAGGAGATGATGCAAGGAAGGAGGCAGAATTTGGTTAGTGTTATTGTATACTAGCAATGTTCAAGATCTTGTTTCAGGAGTTGATTTGACTTATTTTTCTGATAGATTATTGTTCAATAGAAGTTTCACATTTCCTTATATTGTGTCAGCAACTTGGCACTGTTCGTCTTGATTTTTTCTCATGATATAGATTTGACTCAGCAAACTGATATCATTGTGGTTTTTTCAAGTTTCTTAGAAAACCAAATGTAGAAGATGTAGGATTCAGCACAGGTTGAAGTTGAACCCTTTTGAAAGCAAGATCCTCACCAGTGTCCTTTATTCCTCTGCAGGTTCCGCTTCTAGTTCTGGTACTGCCGCTGGTGCTGGTGCTGTGTCATCTACATGA

>FvSnRK3.7

ATGGCTAATGAGAAGAGCGCCGGTTCCGCCTTGCTTCACGGAAAGTACGAGCTGGGCCGGATGCTGGGACATGGAACCTTCGCGAAGGTGTACCATGCCCGGAACTTGAAGACGGGAAAGTCCATGGCGATGAAGGTGGTGGGGAAAGAGAAGGTGATCAAGGTCGGAATGATGGAGCAGATTAAGAGAGAAATCTCGGTGATGAGGATGGTGAGGCACCCCAACATCGTCGAGCTTCATGAGGTCATGGCAAGCAAGTCCAAGATCTACTTCGCCATGGATCTCGTCCGCGGCGGCGAGCTTTTCGCGAAGATAGCGAAAGGAAGACTGAAGGAGGACGTGGCCAGAGTCTATTTCCAGCAGCTGATCTCCGCCGTGGATTTCTGCCACAGCCGAGGAGTCTACCACCGCGATCTGAAGCCGGAGAATCTCCTGTTAGATGAAGACGGTGACTTAAAGGTCACTGATTTCGGGTTGAGTGCTTTCTCGGAGCACTTGAAGCAAGACGGGCTGTTGCACACCACTTGCGGCACGCCGGCTTACGTGGCGCCGGAGGTGATCGGGAAAAAAGGCTACGACGGAGCTAAGGCGGATCTTTGGTCGTGTGGAGTTATCCTCTATGTCCTGCTCGCCGGGTTTCTTCCGTTTCAGGATGACAACTTGGTCTCCATGTATAGGAAGATTTACAAAGGTGACTTCAAATGTCCGCCGTGGTTTTCTTCCGAGGCGAGAAGACTAATCACGAAGCTTCTGGACCCGAACCCGAGTACCAGAATCTCCATTGCGAAGATCATGGATTCGTCTTGGTTCAAAAAATCTATCCCGAAAACGGTGAAGTCTAAGAAAGAGCGAGAGTTCGATGAGACTACGGAGAAGACTTCGAAGCAGATGGAGACATTGAACGCGTTTCATATCATTTCGCTCTCTGAAGGGTTCGATTTGTCTCCTCTGTTTGAGGAGAAGAAGAGGGAGGAGAGAGAGGAGCTGAGATTCGCGACGACGCGGTCGGCGAGCAGTGTCATATCGAAGCTGGAGGAGGTGGGGAAAGCCGGCAAGTTTAAGGTGAAGAAGAGTGACTCCATGGTGAGGCTGCAGGGCGAGGCGAGCGGCAGGAAAGGGAAGTTGGCTATTGCGGCGGAGATTTTCGCTGTGACGCCGTCGTTTCTGGTTGTGGAGGTGAAGAAAGACAATGGTGATACTCTGGAGTATAATCAGTTCTGTAGTAAAGAGCTCAGACCGGCGCTCAAAGACATTGTCTGGACCAATTCGGCCCCCCCTGCTTGA

>FvSnRK3.8

ATGGAGAACAAGAAAGCAAACATATTGATGCACAAGTACGAGCTGGGGCGCCTTCTCGGGAAAGGTACTTTCGCCAAGGTTTACCATGCTCGAAACTTGAGGACCGGCCAAAGTGTTGCCATTAAGATCATAGACAAAGAGAAGGTGCAACAGGTTGGATTGATTGATCAAATCAAGCGTGAAATTTCGGTCATGCGCCTTGTTAGGCACCCCAATGTTGTTCAGCTCTATGAAGTGATGGCCAGCAAGACCAAAATCTACTTTGCCATGGAGTATGTGAAAGGTGGTGAGCTCTTCAACAAGGTTGCCAAAGGGAAGCTCAAGGAAGACATAGCCCGAAAATACTTCCAACAGTTGATTGGAGCCGTTGATTACTGCCACAGCCGCGGAGTTTATCACCGTGACATCAAGCCAGAGAATCTCCTGGTTGATGAGCATGGTAACCTCAAGGTCTCAGATTTCGGGCTGAGTGCATTGATAGAGTCAAGAGGTCAAGATGGTCTGTTGCACACCACTTGCGGAACTCCTGCTTATGTAGCACCAGAAGTGATCAACAAGAAAGGTTATGATGGTGCCAAGGCAGATACATGGTCATGCGGGGTAGTCCTGTATGTTCTTTTAGCTGGTTTTCTTCCATTCCACGACACAAATCTCATGGAAATGTACAGGAAGATCAGCAGAGGAGACTTCAAGAGTCCACAATGGTTCCCTCCAGAGGTTCGTAAGCTACTTGCACGGATTCTTGATCCGAATGCCACCATGAGAATAAGCGTGGATAAGATCATGGAGAACAGTTGGTTTAAGAAGGGGTTTAAGCATATTGATGCCCCGTTACCAATTCCATGTGATCCAAGCACATCTATCAGTGATGTGCATTCTGCTTTTGGATCACCAGACAGTTCAGAAGGCAGTTCTAATAGGAAAGCGGAAACTACTAATGCAGCAAGCCCCATGAGGCCAACTAACTTCAATGCCTTCGACATCATATCTCTCTCACCGGGATTTGATCTATCTGGTTTGTTTGAGGGTGATCACAAGCATAGATCATCACAGTCACGATTCACCACTACAAAACCAGCATCTACTATTGTTTCGAAATTTGAACAGATTGCACAAATGGAGAGATTCAGATGCATGCAGAAGGACGGGACTGTCAAATTGCAGGGCAGCAGGGAAGGAAGGAAAGGGCAGCTTGGTATTGATGCTGAGATTTTCGAAGTCACACCTTCGTTTTTCGTTGTGGAGGTGAAGAAAACAGCTGGGGACACATTGGAATACATTCAATTCTATGACCATGATTTAAAGCCCTCTCTTAAGGACATAGTATGGACTTGGCAAGGAAATGATCCACAGCAGCAACACCAGCCAGCAACTCAAGTCTCTTGA

>FvSnRK3.9

ATGCCGGAGATCGAGGTCGTGTCCGACGCCGGCGACGGCGCATCGGAGGCCTCTTCGTTGGACGAGACCGGTGGCGCCCTGTTCGGGAAGTACGAGCTGGGGAAGCTCCTCGGCCGCGGCGCATTCGCCAAAGTCTACCACGCGCGTGACGTCAGCTCCGGGCAGAGCGTGGCGATCAAGGCGGTGAGCAAGCAGAAGGTGCTGAAAGGCGGCTTCACGTCGAACGTGAAGCGCGAGATCTCGATCATGCGGCGGCTGCAGCACCCCCACATCGTCAAGCTCTACGAGGTCTTGGCCACCAAGACCAAGATCTATTTCATCATGGAGTTCGCGAAAGGCGGCGAGCTTTTCGGGAAAATCTCCAAAGGTCGGTTCAGCGAGGATCTCAGCCGTCGGTACTTCCAGCAGCTGATCTCCGCCGTTGGATACTGCCACTCACGCGGAGTCTACCACCGTGATTTGAAGCCGGAGAATTTACTCTTAGACGAGAATTGGAACTTGAAAGTTTCGGATTTCGGACTCTCCGCCGTGACGGAGCAGATCCGACCAGACGGGCTTCTCCACACTCTCTGCGGCACCCCGGCTTACGTGGCGCCGGAGATTCTCGCCAAGAAAGGCTACGACGGCGCCAAGGTGGATATATGGTCGTGCGGGATCATTCTCTTTGTTCTCAACGCCGGCTATCTTCCGTTCAACGATCCCAATCTCATGGTAATGTACCGGAAAATCTACAAGGGGGAGTTTCGCTTTCCGAGGTGGACGTCGCCTGGCCTGAGACGGTTGATTTCGAGATTGCTTGACACGAACGTCGAGACGAGGATCACCGTCGACGAGATCATCAAGGATCCTTGGTTCAGTGTTGGCTACAAGGACGTCAAGTTTCACTTGGAGGATTTTAACTTGAAGGAGTGGAGGGACGAGGACAACGACACGCCGTTGAATGCTTTTGATTTGATTTCATTCTCGTCGGGGTTCGACATCTCGGGGCTGTTCCGGAAGCCGGAGATATCCGACTGCGGGGAAAGGTTTGTTTCGGCAGAGACGCCGGAGAGGATAATTCAGAAGGTGGAGGAGGTGGCATTGGCGGAGGGGATGACGGTGATGGAGAAGAAGACCTGGGGGGCAAAGTTGGCAGGGCAGAATGGTAATTTGGTAGTTGCTATTGGGATTTACCGTTTGACGGAGAAGCTGGTGGTGGTGGAGTTGAACAAGAGGGAAAGAATAGGGGAGAATTGCCAGCAGATATGGAAGGACAAGCTGAGGCCGCAGCTGTCGTGTTTGATATACAAACCGGAAGAAGAACAAGTCTCCGGTGAATAA

>FvSnRK3.10

ATGATGTGGATGTCATATCCACAAAGTAGGTCTCATGCACATATTACCATATTAGTTCTATCAAATTTCCAAAACTAGTATAAAGTAACTAATGTTTTCAAAGTCATCAACAAACAAGCGTGGTGTTGTTAAAATTGAAATGAAACAAAGCTAACTTTGATACAAAGCTAACACATGTACAAAATAACTAATGTCTTTAAAGTCATCGACAAACAAGCGTGGTGTTGTTTTTTCATAAAATTGAAATGAAACAAAGCTAATGTTGAAACAAAGATAACACATGTGACATGTCTCGGTTTTTATTAACTTTATTCGTTCATTTTTAGATGTAAATCGACTTAAAAGAAAGGGTTATATAAAAATCATTTAGGAGCTGAACATAATTTCTATATATGCATCGAGTTACAGACCATGTGAATATGCTGATCATACTAACAAAGTTTCACTCAGCATCTAAATTTATGAAATATTATTACCAATAATACTATTATTTACAGCGTTACGCAAAATAATATAGGAAGATAAACGACCCGTCACGTCATCTGGAGGCTATCACAAAATTTCAGCGCTTAAATTTGGACTTTCTCTTCACCTCTCTCTTCCCATCTAGAGCTTCACAGCTTCGTCGGAGCCCAGAGACCCCTAGAATCACCTCACTCCGGCTTCAGAAGGTTCCCCTTCGCACGTGCCTCGCTTCTCCGGCGACCGGAATATTCGCATTTCCCGGTAAGTATCACGGCCTTATGCTCTCGATATTCTCTTTCCGTTTCAATCGCCGATTCGGCCTCTGCGATCGCCGGTTTCCGATTCCTTCGTCTTTCGGTTGCTTGATTCCTCCGTTTTTTCGCAGTGAAATTGAAGCTTCTTGAGGTTTGGGAGCTTTTTTGAGCTTCGGAGGTCAATGAGAGATTAGGAAAGACGATGATGAAGAAGAAGGTGACCAGAAATGTAGGCAAGTATGAGGTTGGGCGAACGATCGGCGAAGGAACCTTCGCCAAGGTCAAGTTCGCCAGGAACGCCGAGACCGGCGAGAGTGTTGCCATGAAGGTCTTGGCTAAAAGCACCATTCTCAAGCACAGAATGGTTGATCAGGTACTCACTTTCTCCATTATCTTGCTCTAATTTAATGCGGTATCGAATTCGGAATCGGTTTGAGTTCAAGCTACAGTAGTTGTTGTTTTAATAAGATGTTTATATAGGTGTGATGAATTGTGTGTTTTCAGATTAAGAGAGAGATTTCGATAATGAAGATTGTCAGGCATCCTAATATAGTGAGGTTGCATGAGGTAATCTCCCCGATTTCGTAATATGTTTTGAAGCTGATGTATGTTGTTGTTGTTGTCATCGCAGCTGAAATGAGTGTTTTATTGCTCTGAAATTCTAGGTTTTGGCTGGCCGGACTAAGATATATATAATTCTCGAGTTTGTAACTGGAGGAGAGTTGTTTGACAAAATTGTGAGGTTTCTTCAAATTTGGGACCTTCATCGTGGCTAATTCACTACTGACTGAGACTTGTGACGATTTGTTTTGATTCATAGGTCCATCAAGGAAAGCTTCGTGAAAATGAATCAAGGAAATACTTTCAGCAGCTTATAGATGCGGTGTCTCATTGTCACAGCAAGGGTGTTTACCATAGAGACCTGAAGGTGCAACTCAACATTCAGTTTGTCAGTAATTTTCACTTGGTTCTGTGATTTTGCGGAAGTGGTTGATTACTGATTCGCTCTTTGCTTTCCTTGTGATTTGCGTTTACAGCCTGAAAATCTTCTCCTTGATGCTTATGGAAATTTGAAAGTTTCTGATTTTGGACTGAGTGCATTGCCCCAGCAAGTAAGCCTTATCTAATTCTGTTTGCATTCAACAACATATGAGAAGTTACTTTGATGCACATGCTCTTATTTCTTTATCCTTTTTAATCTCTCATTGGCGTTCCTGTTTTGCCTATATCCTGAGCAGGGGGATGGTCTTCTTTTCACAACATGTGGAACCCCAAACTATGTCGCCCCTGAGGTAGATCTTTTACACATTTTGATGCTGCATTTACCACAAAAAATGATAAGATGGAAATGATGTTTCTTAGATTATAGCCAAAAACAGTAATTATAGAAATCAGGTGGAAGAGAGGAAATACAGGAGGCTAGATAATATTGTCTCAATACCCTGGCCTAAGGAGTTGGAGGCGTAGTAAGGAGAGAACATTCTTTGGACGTCTTACTCATTGTGGATCTAGGAGTCTAGGTTGCTTCTTCTTTATTAAATTATTGTTTCTCACCAAATTAAGGTTACTTGGACATGTATGTATTTACACTTCCTAGTTTCCTAGCTGACATCTTTACAGCTCATAGATAGCTTTACAGAGCAAAATTTAATTTCCAGTTACAACCATGTTATCATGATTGGTTATGCAACACACATCTATGACTTTCTAGAGTTTCTTTGTTGTCTCACAAGTTATATATATTTTCCTAACATCAAAGCCGTTCACCTTTAAAGACAAAGTTCTTCTTTGCTTCCCTTTAAATTATGCTGAATGTGTGAGAAATGGAGGGAGGGAGGGGAAGACAAAACTAATATAATGCTGATTATGTATCATTGGCAAACAGGTGCTGGGCAGTAAAGGTTATGATGGTGCAGCTGCTGATGTTTGGTCGTGTGGGGTCATCCTTTATGTTCTTATGGCTGGATACCTCCCATTTGATGAGGCAAACCTTGCTGCCTTATATAAAAAGGTATGTTGAACTTACTGGAATTAATATGCATAAGGAATGCATATAAATTTGGAATTGCACACCATGCCTAGCACAAGGGAACTTCCTTCTTTCCCCCGTCTCTTTTTTCCCTTCTGAGTCGCTTTATATAGACTTCAGGATCACTTTTATGCATATAGCATCCATTGGAGAATCTTCACACCTCACCATCACTCGAACAAATCTTTCTGTGTAGACTATCATAATTATAATATTTAATATAGTTTTAACTCTTCTTTTGTCTTGTCTGTAGATTAACGCAGCAGAGTTCTCCTGCCCGTTTTGGTTCTCTCCAGGGGCAAATTCATTAATACATAAAATACTTGATCCCAATCCTAAAACTGTGAGTATATTCTTTCACCATATGCAATTTGAGTATTGTCTGTGCCAACTGATGTTAAGGTCTATACTAGTAATTTCATATCATATATATTTTGTTTGGGGAAAATCATAATGATTATTCAAATATTATAGCCAACTTAGTTATAATGTCAATTGTGAAAGAAGAAAAAGGACTACGAAAACCATATCTGCATTGCGGAGTCAGGGTTATGGTTTCCCTTTGCTTTCTTAACTAGTTCTCTTGCTAATGCAGCGTTTTCGGATTGAAGAAATCAGAAAGGATCCATGGTTTAGAAAAAATTATGCGCCTGTTGAATATAGAGAAGATGAGGAAGTCAGTTTAGATGATGTTCGTGCAGTCTTTGAGGACATTGAGGTTGGTTGGCCTTTTTCTTTTGTTCTACTCCATTTTCTTTGAGGAACTTTGTAATTGGCATTATTTATGCATTTTAAAATCTATTTAGATTGCTAAGTAAGGCAGTCTGTGTCAACTGTTGGTATTTCACAGGACCAATATGCAGAAGAAAGAACAGATAATAAAGACAGCGGTCCTTTATTAATGAATGCATTTGAGATGATTACCCTCTCCCAAGGGCTAAATTTATCTGCTCTGTTTGACAGGAGACAGGTATAGTCGTATACTTTACGTCTGTCTGCATATATCTCTGAGAATGACCCTTGCCGGATATTGTTTCATAGTTCCACAAGTACATCTGTAACTTACTCTTTTAGTAGAAAGTTTGAATGATCACATTTTGCTTCTCTGTAACTTGCCTTTTTATAGAAAAATGTTACTTCTCTGTTAGAAGTACAAAGAATTGTTTCTTTGTATTCCTGCAGATTTATCGGATCTCTCTTTTTCTGGTTTGTCTGCGTTGCTCACATAGTTTAGTAAAAGCTCTCTATAGGACCTATAGTATAGGTTGATTCCCAAAAACATGCAAGAAATGTGATGATGTAGTGTTGCAAAATCTATCCGTACTGGAATTCTCTTTCTCTAAAGTCTCTTCTCCTGCAGGATTATATAAAAAGACAAACTCGTTTTGTATCCCGCAAACCAGCTAAAGTTATAATTTCAAATGTAGAAGCTGTTGCAGAATCAATGAGTCTCAAGGTCCATACACGTAATTTCAAGGTATGTTTTGTAATTTTACCATTACTTATTAGTGTATATAATCCATCTTCCCGAATCTTATACGATGACTAATGAGAGGATTTGAGGTTGTAGTATAACTGGTTTGAGTACCTTGTTGTGTACTGAAAGCCAATATGTATTACATATGGCGCTACCTGAAAGTTGTTTAAATCTATATCAACTTTTATAAGACGTGTTCTAATGTAGTTTTTAATTTTGCATCAGACAAGACTTGAAGGGATATCTGCAAATAAGGCTGGACAATTTGCAGTTGTCCTTGAGGTATTAAGCGTTATCTGCATGCATATTTCATACTTTAAGTATCTTTCCAGAGGAAGCCTTGAATTGATTCCGCCGTTGTTTAACATGGTCGTTATCCTACCTCATGTAGATTTTCGAAGTTGCACCATCCCTTTTCATGGTAGATGTTCGAAAGGCAGCTGGTGATACTCTTGAATATCACAAGGTCAGATTCCTACTCTGATTATTCAGATTCATGAACTCAAAAGATTCTTACATTGTTTCTCCCCCTTACACTACATTGTGAATGCCTGTGGATCTGTTTTGCAGTTTTACAAAAATTTCTGCGCCAAGTTAGATGACATTATATGGAAACCAAAAGACGGTATGGCCAGTTCCAATATACTTAGAACGACGACTTGCTGA

>FvSnRK3.11

ATGGAGGAAAGGACGGTGTTGTTCGGCAAATACGAGACGGGAAGGTTGCTGGGCAAAGGCACCTTCGCCAAAGTGTACTACGGCAGACAAATAGAGACCAACGAAAGCGTGGCGATCAAAGTGATAAGCAAAGAGCAGGTTAAGAAGGAAGGCATGATGGAGCAGATCAAGCGCGAAATCTCCGCCACCCGATTACTCCGCCACCCAAACATCGTCCAACTCAAAGAGGTCATGGCCACCAAGACCAAAATCTTCATCGTCATGGAGTACGTCAAAGGCGGCGAGCTGTTCGCGAAAGTCGCCAAGGGAAAGCTGAAAGAAGATCAAGCCCGCAAATACTTCCAGCAATTGATCAGCGCCGTCGATTTCTGCCACAGCCGCGGAGTCTCCCACCGCGACTTGAAGCCCGAGAATCTCCTCCTGGACGAGAACGGGGACCTCAAAATCTCCGACTTCGGGCTCTCCTCGCTCCCGGAGCAGCTCCGAAACGACGGCCTTTTACACACGCAGTGTGGCACCCCTGCTTACGTGGCGCCCGAGGTCCTGAGGAAGAAAGGCTACGACGGTTCGAAAACTGATATATGGTCGTGCGGAGTGATTCTGTTCGTGTTGCTGGCGGGGTTCCTTCCGTTTCAAGACGAGAACATCATGAAGATGTACCGCAAGGTTTTCAAGGCCGAATTCGAATGCCCGCCCTGGTTCTCAACCGAGGCCAAACGCCTCGTCTCCAAGTTGTTGGTGTCCGATCCTGAGCGCCGTATCACCATCCCTGAAATCATGCGCGTGCCTTGGTTCCGCAAAGGCTACACGCGCCCCCTCGCCTTTTCGCCTCCCCCTGCTTCCTCTGACAAGTCATTTGATGAAGATTTTGGTTCTACTGCCCCTGCTCCTGCTGATGCTGCCAACCACAAGTCGCAGTCGCCCAACTTCTTCAACGCGTTCCAGTTCATCTCATCCATGTCTTCCGGATTCGATTTGTCCAATTTGTTTGAGAGCAAGAGAAAAGCAGGGACCATGTTCACCTCCAAGTGCTCCTCAGCGGCTATTATGGCCAAGATTGAGCATGCTGCCAAGGCATTGAGCTTCAAGGTGGGGACGGTTAAGGACTTCAAGTTAAGGCTACAAGGCCCAAACGAAGGCCGGAAAGGGAGGCTTTCGGTGACCGCGGAGGTCTTCGAGGTGGCGCCGGAGGTCGCGGTGGTCGAGTTTTCCAAGTCTGCCGGGGATACTTTGGAGTACGCCAAGTTTTGTGAGGAAGATGTTAGGCCAGCATTGAAAGACATTGTCTGGACCTGGCAAGGTGACGGTAACAAAGTCGATGGTGAAGAATGA

>FvSnRK3.12

ATGGTGATCATAAGCAAGGGGCAGGAGAAGAGCAGTGATCAGAGTAAGAAGGGAATGCGACTTGGGAAATACGAGTTGGGGAAGACTTTGGGTGAGGGCAATTTCGGCAAAGTCAAGTTTGCTAAGGACGTCGGCTCCGGCCAACCTTTCGCCGTTAAGATTCTTGAGAAGAAGAGAATCACTGATCTCAATATCGCCGACCAGGTTCTTTGTTTTTTTTTGGTTTTCAAAGTTTGATTCTTTTTCTTTGTAAAGTTTCAATCTTTCATTTGATGGGTTTTCTGGGTAGTGTTGATGATTGGAAAAAGTTTCAATTTTTTTTTCTATCTTTTAAGTAATGGGGTTGTTTTGGTTAAGTTTCTTGATTGTTTTTGCTTTCATCTTTATCTTTTGGTTCAAAAGTTTTCATCTTTTTGTTTTTTTAGCTGGAATGTTTTGATCTTTCATCAATGGGATTTCTGGGAGGGGCTTTAGTTTTGATGTTCTAAATGGTTTTGCATGCAAGATGTGTTTTGCGATGTCATTTGGCTGTATACGAGTTAATGTACAAACAATTTAGAAACTGAAACTGGCATATTTGGCTCTGGTAAAAAAAAAAAAAAACTGCCACATTTGGCATTCATGACGTTTATAGTCGTCTATTATGATCTGACTTTTCTGCAACAAGAAACTGAGAATTGGCATTCATTGTTTATGGTTCAACATAATACTACTTCAATTTTCGTTTCTGTTTTAGTTTTGTGTTTATTGGTTCAATAGTAACAAAATTCTTCATTGCAGATAAAGAGGGAGATTGGCACTTTGAAGCTTCTAAAACATCCAAACGTAGTCCGATTACATGAGGTATTCTTCAGGATATGCTAAACTTGAACTGTGCCAACACTACTGTCTTGGTTTATGTCCGTTATTTTTCATGAACCTCAATCATCAGCAGCATAATCTTTGTGATTTTCCACGAATTTCTCATATGATAATGATCCCATATTTTAATGGTCCATAAACAGCATCAGCACCAATCTGGGATTTCCGGTAGAGCTTAATGTTCTTTATTAGAGCACATGCTGAGTTCTTACTACTAATCAATACGATTACGCGTTTGACATAATCTTTGGTAGATTAGGTAGCCAAAACTGCGTGGCGTACAGTAACTGTATCCTATTAGCTTCCTAAATAGGATTATCACTATATCAGCTACTTTTACAGTTACCCTGAACTTTAAACAGTCTCCATTCTTCGATCTGATTAATCCACCTAATACGACCTTCACCGGATCAAATGACTTCTCTGAGATGGCGATAGATCTCAGCTTGATAAACATGTGCAACTACTCTCCACAGATTTGTTTGTTGGTTGCAAATTATAATGATGAGCTGGACCACTGAATTATTTTCTTGGGGCTCAGTTTCAATCAACCACAGTACCACACCAAGTGAGCCAACTGTCATATTGGCTCCTTGAGAGAAACCTTTGCCCCCATTCTGATGGATAAGATCATCAACACTGGTTGTGTCTACCAGTATCTTCCCAGACATTAAAATCCAAGCATGATTGCCTCAACTTGGATATATGATATGTTGTTTTCAGGGTATAGTTTTACTTAATGTAGCATAGGATATATCATAGATTCTTGGATACGTCCTTTTGGGACTAATATGAAACGAAAAGTATTTAACAGAAAATAAATACTAGGATGATGCTGTGATTAATGACTGGCCTTTTAGAATATGAATTTGTTGAGGTAGAACGGGTCTTTTGAATTACCAGCCACTTGCTTTGCCTTGTGCAAATGTGGCATAGGTATCCATTTGTTCAAGTAGCTGTATTTAAACATTGTTCCTTTTGAATGTTTTCCCATTGGTCAGCCTATGCATGGGATCAAGTACTAAACCATTCTATGCAATTGAGACATATTTGTTAGCAACTTAGCACCAATAGTTTGTCGAAAAGCTTGGTTGCACATATGATTGAGAAAGCACAAAAAATATTCCAACCATTCACATATTTTTACATGTTGAATAGATTCTTCCTTTGATCTCAATAGAGTGCTTTAGACTAGTTTTTAAACATCAAAGCGATGGCCAACAATGCTTCTACTAAATCTGCTAATTTTACTTATCGTGGAAACTATGCAGGTCGTGGCAAGCAAAACCAAGATTTACATGGTCTTAGAATATGTTACTGGCGGGGAATTGTTTGATAAAATTGTAAGGAGCTCTTAGTTTGAATTTTATATTCAGAGGCAGGAAGTTGATTATGCTAATAAGTATTTTATGTTAGCAGGCACAAAAGGGAAGACTTAAAGAGTCCGAAGGTAGAAAGCTTTTTCAACAGTTAATTGATGGTGTGAGCTACTGCCACAACCAAGGTGTTTTCCATCGGGATCTTAAGGTACTTTCTACTTTCGGCAGGATACTTAATGTTGTATAGGTTTCTAATTTGCTCACATCTTATATTGGATGTTTTGATGGCAGCTGGAGAATATTCTTGTTGATTCCAAAGGAAACATAAAGATATCTGACTTTGGCCTTAGTGCTTTGCCGCAGCATTTTAGGGTACATTATTCTTCCCTTCCAATCTAAGCATTAAAGTTTAACATTTTTGAGTCCTGTAAACCCTTGCATTATGTCTGAATATCAGGAAGATGGTTTGCTGCATACAACCTGTGGAAGCCCCAATTATGTTGCCCCTGAGATCCTTGCTAATAGAGGGTATGATGGTGGCACCTCTGATATATGGTCATGTGGTGTCATCTTATATGTCATTCTAACAGGGTATCTCCCGTTCGATGATAGGAATCTTGCAGTTCTCTATCAAAAGGTATTGACACCTTCTCTTTGGTCTTTCCAAGTTATATAGCCGGGTAACCAAAAATCTTGTATCATCTGATATTCATTAAAATGAAGTTATCGTTTTTGGATGGATTTCAGATCTTGAAGGGGGATGTTCAGATACCCAAATGGTTATCACCTGGAGCACAAAACTTGATAAGAAGGGTTCTCGATCCCAGTCCTCTCACCAGAATAAACATGACAGACATCAAGTCAGATGAATGGTTCAAGCAGGATTACTTTCCTGCAAAAGCTGACGAAGAAGAAGAAGATATAAACGTTGATACCGAAGCTTATTCAATAAAAGAAGTGGTATGAACCAACATTTTATGTGAACTGCTCACTTTTTATGTACATCATAGTTTTCTTATCTGCTATAGCTGAGACAATTGTTTGTTTCTGTATCAGCCATCTGAAGGGGAAAAGAGTCCAGATTTGCGGCACTCACCCACCCTTATCAATGCCTTTCAGTTGATTGGAATGTCTTCATGTCTAGACCTCTCTGGATTCTTTGAGAAAGAGGTGAGTGTAAAACTAATCATATAACACATCTCTGAAATGATTTCTTCATATTGAATAACCCATGTCCGCGAAGTTGATATCTGTATTCTTTCTAAATTAATATAGGATGTGTCTGAGAGGAAGATCAGATTTACTTCCAACCACTCTGCAAAGGATTTGCTAGAGAGGATTGAAGAAATTGTAATAGAGATGGGATATGCTGTCCAGAAGAAAAATGGAAGGGTAAGTATTTTGTAACACGTTATCTTCCCTTCACACAGAACCCAGAAATAGCTCTTTTTAAACGATATATACAAACTGGTTTTTTGCTGCAATATTAGGCTAAAATAATGTTGCTACCTTCAACATTTCAGTTAAAGGTGATGCAAGAGAACAAGGGGCAGAGAAATCTGGGCAGTCTCTCAGTTGCAGCAGAGGTATTTCATTTAAACCCAGAAACTTGTGCATGTTGTAATGTTTACTTGTTTCTCAAGTAACATGTTTGTTACTTTAGCGGTGAACTCATAGTTGTCCCTGTAATTGCATCAGGTGTTTGAGTTAAGCCCAACATTACACGTAGTTGAATTAAGAAAATCATACGGAGATCCGTCTGCATATAGACAGGTATATACTCCATTACTCCCTCTCTCTACAACCTTACTGTCTCTTGCTCTCCTTCCTTTTCTCTCCTCTCAGATTTCAGGCACTGACATCAACTTTTGGGTATGTGCAGTTGTGTAAAAAGCTATCAAATGAGTTAGGTGTTCCATCGAGCCAAGAATTGTTGGCCAGCGAGGTATTGAAGTCCAGTTCTTTGCAGAGTCAAACGGCGTAG

>FvSnRK3.13

ATGGGCCGTGGGCCCCCACCACCCACCCCTTCCTCCGCCGCCGCCGCCGCCGCCACCACGACCACCAACCTCCTCGGAAAGTATCAAATCAGCCGGATGCTGGGCCGCGGCAGCTTCGCCAAGGTCTATAAGGCCCAAACCATCGCCGACGAGACTCCCGTCGCCATTAAAATCATCGACAAGCTCAAAACCCACGCCGCGATGGAGCCTCTGATCCTCCGGGAGATCTCCGCCATGCGCCGTCTCCAGGACCACCCTAACATTCTCAAAATCCACGAGGTCATGGCCACCAAGTCCAAGATCTACATCGTCGTGGAGCTCGCCACCGGCGGCGAGCTGTTCGCCAAGATCTCACGGCACGGCAAGTTGCCGGAGTCTCTGGCCCGCCGCTACTTCCAGCAGCTCGTCTCCGCCCTCCGCTTCTGCCACGAGAACGGCGTCGCTCACCGCGACGTGAAGCCGCAGAACCTCCTCCTCGACGGCAACGGCGACCTCAAAGTCTCCGATTTCGGACTCTCCGCCCTACCGGAGCAGCTCAAAAACGGCCTCCTCCACACCGCCTGCGGGACTCCGGCGTACACGGCGCCGGAGGTGCTCTACCGGGTCGGGTACGACGGGTCCAAGGCCGACGCGTGGTCCTGCGGCGTCATCCTCTTCGTCCTCCTCGCCGGACACTTGCCGTTCGACGACAGCAACCTGGTGGCGATGCACAAGAAGATCCAACGCCGCGATTACGTCATACCGGCGGCGATTTCGAAACCGGCGCGGCGGATCATATACCAGCTCCTCGACCCGAACCCGAACACGCGGCTGAGCGTGGAGGCCGTGATGGAGAAGGCGTGGTTCCAGAAGGCCATAGATCTGAAGCTAGTCTCCGACGGCTGCGATGTCTTTGAATTGGAGAAGCCGCCGGCGAAATGCGACGTCGTTTCGGGGATGAACGCGTTCGACATAATATCCATGTCGTCGGGGCTGGACTTGTCGGGGCTTTTCGAGGCGGAGAACAGGAGCGAGAGGCGGTTCACGGCGAATGTGGCGGCGGAGAAGGTGGCGGAAAAGGTGGGGGAGGTTGGGGAGAGGATGGGGTATAAAGCGGAGAGAGGGAAAGGAGGGATGAGCGTTGGGTTGGGGAAAGGAGGGAAAGGGAAAGGACGACGTGTCGCTTTGGTGGTGGAGATGATGGAGGTGGCGGCGGGTTTGGTTTTGGGGGAGGTCAAGATGGTGGAGGGTGGTGTTGAGTTTCCTGAGCTGCTTTGGGAGGACTTGAAGACTGGGTTGGGGGACGTTGTGGTGTCTTGGCAAAACGGTGGCGTTTAG

>FvSnRK3.14

ATGCCAGAGATCGAACAACAGCAGCAGCAAGTAGTGCTCCGCATCCCAGACAATGCCCTGTTCGGGAAATACGAGCTGGGCAAGCTTCTGGGATGCGGGGCCTTCGCGAAGGTGTACCACGCAAGGAATGTGTTCACCGGCCAGAGCGTGGCCGTGAAGGTGATCAACAAAAAGAAGCTAAACGGTACCAGCCTCATGTCGAACGTGACACGCGAGATTTCCATTATGAGACGGTTACGGCACCCAAACATTGTCAAGCTTTACGAAGTCATGGCCTCCAAGACTAAGATCTACTTCATCCTTGAGTTCGTCAAAGGCGGCGAGCTTTTCGCTAAGGTCTCGAAGGGAAGATTCTCGGAGGCTCTGAGCCGGAAATATTTCCAGCAGCTGATCTCCGCCGTAGGGTACTGCCACTCTAGGGGAGTCTACCACCGTGATCTGAAGCCGGAGAATTTGCTGGTGGACGATAACGGGAATTTGAAAGTTTCGGACTTTGGTCTCAGCGCGGTGACGGGTCAGATCCGACCCGACGGGCTGCTCCACACGCTTTGCGGGACGCCGGCTTACGTGGCGCCGGAGATCTTGACGAAGAGAGGGTACGACGGAGCAAAGGTGGACATGTGGTCGTGCGGTGTGATTCTTTATGTATTGAACGCCGGGTTCTTGCCGTTTAACGACCCGAATCTGATGGCGATGTACAAGAAGATTTACAAGGGGGAGTTCCGGTGCCCCAAGTGGATGTCGCAGGATTTGAAAAGGTTCTTGGGTCGGGTCCTGGATACCAACCCGATGACCCGGATCACCGTGGACGGCGTCCTGAACGACCCGTGGTTCAGAAAAGGCGGCGAGTACAAGGAGATCAGTTTCTATGATGATGACAAACCGGACGAGGAGCAGAAATGCATGACGAATTTGAATGCTTTCGATATCATTTCTTACTCGGCGGGTCTGGATCTTTCCGGGTTGTTCGAGTCGACTAACCCGATCCAGGACTCGGAGCGGATCGTGTCGTCGGAGACGGTGGAGACGATGGTGGGGAGAGTGGAGGAGTTTGCTAAGGAGGAGAAGTTGAGGGTGAGGAGGAAGAAGGATTGGGGGATGGAAATGGAAGGGCAGAATGGTAATTTGGTGATTGGAGTGGAGGTGTCGAGGTTGACGGAGAGTTTGGTGGTTGTGGAGGCCAAGAGAACAGGCGGGGAGTCTGGGCCGTTCAATGACATGTGGAATAAGCTCAAACCCCGTCTTGTCTTAGGCCGTGAAGAAACAGTCGAAGAAGACTTGTCTTTGATCGCTTCTTCTTCTTCTTTGTCGTCTGTATAG

>FvSnRK3.15

ATGGTGGTTAGGAAAGTTGGCAAGTATGAAATTGGGAGGACGATCGGTGAAGGGACCTTTGCGAAGGTGAAGTTTGCTCAGAACACTGAGACCGGTGAGAGTGTGGCCATGAAGATCATCGATCGGAGCTCCATCATCAAGCACAAGATGGTCGACCAGGTAAACCCAATTAGTGCAGCCTTTTGGTTTTGAATTGAGTAAAGTTTGCAACTTTTTCTGTTGGGTTGTATTGGTGAGGGAGTTGAGCTTGACCCAGTTAGAAAATGATTCTAAAGCTTGCAACTTTCTTAGTTGGGTTGTTGATCATCTATGTTGGTTTTGGATCGAGTAGAAATTTCCAAAGCTTGCAACTTTTATTTTAGTTGGGTTGTTTTGGTTTAGGAACTTGAGCTTGACCCAGTTTGAATATTTGTGTTTGTTTTTGAAAAATGATTCTAAAGTTTGTGAGTTTCGAATTTAGGTTGTTGTAACTTGGGAGTCAATTCACTTGTTTGCTATGTTTAATTTTGAATTGATTGGAGAGGAAATATTGACCATTTTTGATTGAGTTGGTTTTGGATTAGGGAGTTGAGCTTGAAATGTTGATGTTTATGATGCAGATCAAGAGGGAGATATCTATAATGAAGCTTGTGAGGCATCCCTATGTAGTTCGTTTGCACGAGGCATGTGATATATCTACTCCCGACTTTTAAGTAGTTGTACGCATAATGTTGAGAAATGTAGGTAGCTAACTGACTCTATGATGATGTTGTGTGGTGTAGGTTCTGGCGAGTCGGACCAAGATTTATATAATATTGGAGTTCATTACTGGTGGTGAATTGTTTGATAAAATTGTGAGTAGTCTCTGACTTCAAATGCCTGTGCTTAAGATGGCAATGGACTGTTTAAGAAAGTGGTGATACGATGTTAACACTGAGAAAAAATTGTTGATTATTCAGGTTCATCACGGACGTCTTAGCGAAGGTGAAGCCAGGAGATTCTTCCAACAGCTTATTGATGGAGTGGATTATTGCCACAGCAAAGGAGTGTATCATAGAGATTTAAAGGTCTTCCTTTTAATGCATCTACCAAATATTTGTGATTGTTTCTTGCTGTCTTGTGAAATTCATGTCATTGTTTTTTGTGCAGCCGGAAAATCTTTTGCTTGATTCCATCGGAAATTTAAGAATTTCAGATTTTGGACTGAGTGCATTGCCTGAACCCGTAAGATTCTGGTCTCATCTCTCTCTTTTTTTTTCTGACATTTTTACAGTTGAGAGATATAGTATGGGATTGTTGGCATATTAATAATCAATGTATGTTGTTTGCACACAGGGAGTCAGCCTCCTTCGCACGACTTGTGGGACCCCTAACTATGTTGCACCTGAGGTACAATAAATTTAGCCACCTTCTGAGTTGGATTTTGACTATACCTCAAGTGCTGAAATGGTTTGTTTCATGCATGTGACTGCTTTGATTTGTATTTATCTTGTAATTCCTTGCTCTAGGTTCTCAGTCACAAGGGTTATGATGGTGCCGTGGCAGATGTTTGGTCCTGCGGGGTCATCCTGTATGTTCTCATGGCTGGATACCTTCCATTTGATGAGCTTGACCTGACCACATTATATAGTAAGGCATGAACCTCTTTCATGTTTATTATTTTTATTATTGTTTGTGGTGTGGAGTACAAGAATTATGAACTGGTATTTTGGACCAATATGTGTAACTGTTAAAGTTGTATGACTAATTAATGTTTTCTCTTACGTAGTCTACTTAATTTGTAGATTTGAGAACTATGCTTCTGAAGAATTGTCGATCACGTAGTTTTTCAAACTAATGCATTTGGTTGTGAAATGTGAAGGAACATGAAATACTGCCTCTGTTCCTTACTTTTTAAAGTCCTTTCACCTAATTTTGACGTGCAGACATTCTAGGCTATTTCATTTCATTGTCTGGACTCTAGCTTAGTTCTTTTTTCAAATTATTTTGCCTCATCTGATATCTACTAGTGATGAAGGATTTGAAACTGTGCTATAATATATAAATGTCAGATGTGATAAGCCATTGTCATTTTTCTTCTGCAGATTGAGAAAGCACAGTTCTCATGCCCAAGTTGGTTTCCGATGGGAGCAAAATCCTTGATTCATAGCATTCTAGATCCAAATCCTGCAACTGTAAGTATTGGCTAGGTTTCTAGACTTGCTCATTTATGTGCACAAAATGTAGGTGTCAGTGTTTTGGGGGTCATGTTGCTTTGGTATATGCAATACATATCATGGAATTGCATAACATGTAACCATCCAACATGAGGAATAGTTGTTTAATTCATAATAACAAATGGCAAAAGAGAAATACTGTTCAGAAGTACTACCAAGTTTTGTGTTTTAACTTTTAAGTGATAAGAAACGGTCTGTTAAACCTATGCTGACAGTTTTGAATTTTCTTTTCGGTACATCTCAACTTTGATAGTTAGAGTTTTAGTTATATCCCAGTTCTTTAACCGCAGCACCCATTTTCCATAGAGTAATAGCCAACAGTTCACCCTTCAGAGACCTTAAACACAGTACCACTAGGTTGTCCTACATTATATCTGGAACCATAGAAGGAAAGGACAACTAGCACAGGACTACCTCTTTTGGGTTTGATGAATGAGGAGTTATTTTAGCAGCCCCCTGAAAGGCTATATATCCTAATTTGACAACACGAGCTATATGAGCATGACATCCATATCTAGATATCTCGATAGAGGCATTAGAACAAGACTGGGCCCAGGAACAGCTTGAAGAGAACTTAAGAAGTTTTAGGTGCTGAAGACATCGGGACAGAACTGAGCAAGACAAAACGAAAAAAATAAAATAAAAAATTAAAAACTCCATGTTGCAGAGGATGATTCTTCCGATGAGGGACATAATGACATATGCAAATTTGATGGTGGTGTTTTTGCAGCATCTAAGGAGGATGACAAGTTGCATTCAAAAAGAATGTGTTTTTTCCAGGTGGCTAAGCAGATCTATAGTAATGCCTATGCCTTGGAGAATCCATCTTTCCTCAAATCAGTTCAGCCTTTGAATTTCACCTTACACCATACTCATACCACGAGGAAAAAGCATAGAAACCTTTTTTGATGCCTGACTCTACCAAAAACCACGGATGCCGACCCATCCCACCCTATTGTCCATTGCTTGCCTGCATATTATCTATAGATTATACGTGGCTACCAAAAACCAAAGATGCCAACCGATCCACACCCTATAAATAATGCCTGTCCATTGCCTGCCTGCATAAGTGCATATTACCTATAGATTATACCTGGCTAACAAAGAGGAGTTAATTGAACTCCTCTTCATGAGTTGTACCAAGTGCAGGAGGGTTGATGGAGATCAGAGATACACATGTCGACAATGGATGAAACAAAAGTGAGATAAATTTTAGAAAAGTCTAGAATTATTAGATTTGCTGGAGTCCGTGTAGTTGTCAGTTACATGATTTTAGATGTTTCATTTAAGATGCATTCTTCGAATTGGGTCATGTTTGCAGTTTTTGAATGTCTTTCATGTCAGTTAGCAATAATAGGCGTTTAGAAGTCATTGATGTGGGTCTTTGAATGTTGGGTTCGGGTATCTTCTGTAGCTCTATATAAGGGTCACTATGTATTGCTTGAACCAGTGGAATTGCTTGTAGCTCTATATAAGGGTCACTATGTATTGCTTGAAACCAGTGAATTGCTTGTGTTTTCTTTCATAGAGTATCTCTTGATTGAAATCTTTGTGAGTTTGTCTGATTTCCAAGAACCCTTCTTGTGTGATTCCAGAATTCCTATGTCCAAGAACCCTTCGTTGTTCTCTAACAACAACACTCATTTCCCCAGATTATCACCCTATAATTAACCCTTGGACCTTAAACACAATACCTTGTCATACATTAATCTGCTTTAGTTCACCATTCTGGGGTTTGGGGCCATGGACTGATGCTAGTATGATAGAATTATGTGGAAGAACGCATTTGGCTGTTTGGTTATACTGGTCTAAGTTCTTGCAACACTATTTCTTTTAGTTCCTATATGGTTCAAATGAAGAATAGGCTACTAGGATGTTCCATAGAAACACATGTAATATTTGCTTTCTAACAATTAAAAAGCGGTAATGTGATTGTTAAAGTTGGTACTTATAGCTTGCAGATTTTTTTTAGGTACTATTTGTATATAATGGAAAATTCTAAACATGAATAAGTTGTATTTGGGGATTGGATTTCTAATTAGAATAGGGGGTTAGTTATTACCAACTATGAATACTGTTTTATTATTAATATTCAGAGTTTCACATGCTTGGCTTCTGCAATTATTTTAAGTTCTCTAATAAGTTTCTTTCCAATTAGAACAGAAAAAACAATGCGAAGTATAATTTATCTGTAGCGTACTAACAATCAGCGATCAGTTCTTAATAATGTAGAACCCTATCAATCTAATAGTGGGAAGGTGGTGCTCCATCTGTGTCCCCTTGTTTGAGCCATGCAAGCCATGGATCTTTTTATTTGGTTTGGTAATTCTATGTTTTCACCATCATACGCAATCTAAAGAGGTTTATTCCCATTAGCATTACCTTATTCTGAATAGTCTTGATGTGCGCCTTTGTTAATTACTATAAGAGGTTTATTCTGACATGGGTCCTTTGTTAATGGGAATAAACCTCTTATCTCAAGGCCTTTATAATGCCGCACCAAGGTTCTCTAGATGTGGTGATTTATCAGAAAAGTATGAACTAGACTAATTACTATAATTGAAGTCTGTGGATTGAATTCTTGGTAGTAATGTTATAAGGTTTATTTTGAATTACTTTCCGTTAGTTATACCGAGTCGTTTCTGAAATGTGTTTATCGTGTGTGGCTTTGTGTCAATGCAGCGTATTACAATTCAACAGATCAGAAATGATGAGTGGTTTCAGAAAAATTATGTTCCTCCCAAAATATTAGAATATGAAGATGTAAATCTTGATGATGTAAATGCTGTCTTTGACGAGACCGAGGTTGGTAAGGTTTTGAGAAGATACATTACTTAGTTGAGATTGAAAGCTATATTAACTTGCTTGATATATATGGCTCTATGTAGGAAGAAAGGGATAGCGAGCACCAGGGAAGTGAGGACATGGGTCCTTTAGTTCTTAATGCATTTGACTTGATCATCTTATCTCAAGGCCTGAACCTGGCGTCAATGTTTGACCGTGGCAAGGTAAGACATTAGCTTATAATTTATTTCCCTCTATCTATAGTAGCTGAGTAAATGATACCTCTACATTTGTATTGTGGCCATTACATGTTGGGTATTTCATATCTGCAGCCTGGAAACTAGCTAATTTAGTAAACTTATCTTTCAATCTGTCACACTATGAATGAATTGAAATTAATCTTGAACATCATGGATTTCAATGTTGTCACATAAGAGATATGGTTTTCTTGTGTCAGTTTGCTAGTAATAAAAATGTATTAATTTCTTTCCTCATTCATAGGATTGTATGAAGTATCAGACTCGCTTTGTCTCACAAAAGCCAGCAAAGGTCGTACTATCAAGCATGGAAGTTGTTGCACAATCAATGGGTTTCAAGACACACATCCGCAACTATAAGGTATACAGCTAGGATTTTCTACACTATTCCACACCTTTGGTCCGTGATTTATTTTTTCTTTCTCCAATATCATGATATATACAAAGTCTTTATTCAGTCTTTCACTGTCTAATGTGAGTGTTTCCTAGGACTGGATAACATTTCTTGAAACATCAACTACTAAAGCAACACACTCTGTTCTGTTATTATTTCAGATGCGAGTGGAGGGTCTTTCGGCAAATAAAACTTCTCATTTCTCCATTATTCTCGAAGTAAGCAGGCACTTCTGATGCTAAGCCTGAAATAACGCTTTTTAAATTAGTAAATTGAATCCCTGATTCTTTTCTTTATTGAATGAAGATTTTCGAAGTTGCTCCAACCTATTATATGGTAGACATTCAGAAAGCTGCTGGAGATGCTAGTGAATTCCTCAAGGTTAGTTTTTAGTTTGAATCTCATTATAAGGTAACTTACTTCACCTTTAGGTGTACTCATGTCAGGTGTGTCTCTCCTTGCGAGTAGTGTACTGCTTTATCTTTAGTTTCTGATTCCCTACTGTTCTACAGTTTTACAAGAACTTTTGTAGCAATCTTGAGGATATCATCTGGAAACCGCCTACTGAATCTAGCAAATCAAGGATCAGCAAGAGTAAAAGTAGAAAGTGTTGATATCTTACACAGCTGCTTTCACTCCACGGTCATTGCTGGACTTGTACTTGACAACGCTTTTGGAGGCATGGGGCATTAA

>FvSnRK3.16

ATGGACAAGGAGGGGGAGGTGTTGCTGGGGAAGTACCAACTTGGGAAGCTCTTAGGGCAGGGGGGCTTTGCAAAGGTCTACCACGCAAGGAGCCTCAAAACCAACCAGATTGTAGCCATTAAGATCATCAGCAAAGAGAAGGTTTTTGAATTGGGACTCGTTGATCAGACCAAAAGAGAGATTTCTATTATGAGACTGCTTAAACACCCCAACATAGTGCAGCTATATGAAGTCATGGCCACCAAAAAGAAGATCTACCTTGTCATGGAATACGCAGAAGGCGGCGAGCTTTTTCAAAAGATAAATAAAAGGAGGCTCAAGGAGGAAGCAGCAAGGAGATTCTTCCAACAACTCATCACTGCTGTCGACTTCTGCCACAAGAGAGGTGTTTTCCATCGCGATTTGAAGCCCGAGAACTTGCTGCTAGATAAAGATGGAGTGCTGAAGGTGTCGGATTTTGGGTTGAGTGCGTTTTCTGAATCGAAGAGGAAACATGCTTTACTGCATACAACTTGTGGCACTCCTAATTATGTAGCTCCTGAAGTTATCCGGCTTGGAGCCTATGATGGAGCTAAAGCTGATATATGGTCTTGTGGGGTGATCTTGTTTCAACTATTGGCTGGTTATCGTCCCTTCGATGACTCAAATCTAAACAACATGTTTAGGAAAATATGTGCATCAGAATATCGATGTCCCCGGTGGTTTTCCGATGACATAAGAAAGCTCTTGTTTGGAATCCTCAACACAAATCCGAATGAAAGGTTTCTTGCTTCAGACATAATGAGAAGTAGCTGGTTTCAAGAAGGACTCAGCTCCAAGATTAAAACAGAAGTGGAAGATGTTGATGGGGAGTCTGATGACTGTGACAAAAGCGAGAACCAAGAGACTATTACACCCGCCACTTTGAGTGCTTTCGATATCATATCTCTCTCTAGTGGATTCGATCTTTCTGGCTTGATGATGCAGAAGGATGCAAAGAGATCAGCTGTGCAGTTCACATCCGCGCAATCTGCGACATCCATCATGACAAAGCTGAGGGATATTACTCGGAAACTGAAGCTGAAATCGAAGAAAGAAGGAGCATCATTGAAGTTAATGAAGGGGGCATTATCCATAGAAGCCGAAATTTTTGAGTTCACTCCATCCTTTCATTTAGTGGAGATGAAGAAATCTAATGGGGATACATTTGAGTTTAGGAAGATGGTAGACGAGGATATAAGGCCGGCTCTCAAAGACGTCGTCTGGACATGGCAGGGTGAGCGCAGTAACAACAATAGCAGCATCTGCGTTTGA
