# Supplementary material for: Genome-Wide Characterization of Snf1-Related Protein Kinases (SnRKs) and Expression Analysis of SnRK1.1 in Strawberry
Source: Genes (Basel). 2020 Apr 16;11(4):427. doi: 10.3390/genes11040427 (PMC7230852; doi:10.3390/genes11040427)
Supplement: Supplementary file 1 [file genes-11-00427-s001.zip › Supplementary File S4.docx]

Supplementary File S4: Coding sequences of strawberry SnRK genes.

>FvSnRK1.1

ATGGATGGAGCAATTGGCCGTGGAGGCAGCAGCGCAGACGCGTATTTACCGAATTACAAGCTTGGAAAAACTCTTGGTATTGGTTCTTTTGGCAAGGTCAAAATTGCCGAGCATGCATTAACTGGCCACAAAGTTGCTATCAAGATTCTTAACCGGCGCAAGATAAAGAACATGGAAATGGAAGAGAAAGTGAGAAGAGAAATCAAAATATTAAGACTGTTTATGCATCCTCACATTATACGACTCTATGAGGTCATTGAAACACCATCAGACATTTATGTTGTTATGGAGTATGTCAAGTCTGGAGAGCTTTTTGATTATATAGTAGAGAAGGGTAGGCTACAGGAAGATGAAGCTCGTAACTTTTTTCAGCAGATAATATCTGGTGTGGAGTACTGTCACAGAAATATGGTTGTTCATAGAGACCTGAAGCCAGAAAATTTGCTTCTGGATTCCAAATGCAATGTGAAGATTGCTGATTTTGGCCTAAGCAATATTATGCGTGATGGCCATTTTCTTAAGACAAGTTGTGGTAGCCCAAATTATGCTGCTCCTGAGGTTATTTCTGGCAAACTATATGCGGGGCCTGAAGTAGATGTATGGAGTTGTGGTGTGATATTATATGCTCTTCTTTGCGGTACTCTTCCTTTTGACGATGAAAACATTCCCAACTTGTTCAAGAAAATTAAGGGCGGGATATACACTCTTCCAAGCCATTTGTCACCTGGTGCGAGGGATTTAATCCCACGAATGCTTGTGGTGGATCCAATGAAGCGAATGACCATCCCTGAGATTCGTCAGCACAAATGGTTCCAGGCTCATCTTCCTCGTTACTTAGCTGTGCCCCCACCAGATACAATGCAGCAAGCGAAGAAGATTGATGAAGAAATCTTACAGGAGGTGGTAAAGATGGGATTTGACAGAAACCATCTTGTTGAATCTCTGCGTGGTAGATTACAAAATGAGGGAACCGTTGCTTACTACTTATTATTGGACAACCGGTTTCGTGTATCCAGCGGCTACCTTGGGGCCGAGTTTCAGGAGACTGTGGAATCTGGTTTCAATCGTATGCATCAAGGTGAGCCTGCTTCTTCACCTGTTGGGCACCGCCTTCCAGGATATATGGAGTTTCAAGGAATGGGCTCTTCACCTTTTAGACAGCAGTTCCCTGTTGAAAGGAAATGGGCTCTTGGACTTCAGTCTAGGGCTCATCCTCGTGAAATAATGACCGAAGTCCTTAAAGCTTTACAAGAACTGCGGGTGTGTTGGAAGAAGATAGGTCACTACAACATGAAATGTAGGTGGGTTCCTGGCACACCTGGTCATCATGAAGGCATGGTCGACAATCCTGTAAACAATAACCATTATTTTGGGGATGAATCCAGCATCATAGAAAATGATGGCATTATGAAGACACCCAATGTTGTCAAGTTCGAAGTGCAGCTTTTCAAAACTCGGGAGGAGAAGTATCTGCTAGATCTGCAGAGGGTCCAGGGTCCGCAGTTTCTCTTCCTGGATCTCTGTGCCGCTTTCCTTGCACAGCTTCGTGTTCTTTAA

>FvSnRK2.1

ATGGAAAAGTATGAGCTCGTCAAGGACATAGGATCCGGCAATTTCGGCGTGGCCAGGCTCATGCGCAACAAAGAGACCAAAGAGCTCGTCGCCATGAAATACATCGACCGCGGCCTCAAGATTGATGAGAATGTTGCCAGAGAAATCATCAACCACAGATCACTCCGCCACCCCAACATCATCCGATTCCGAGAGGTGGTTTTGACTCCTACGCATCTTGCTATTGTTATGGAGTATGCCGCCGGCGGAGAGCTCTTTGAACGTATTTGCAATGCTGGCAGGTTCAGCGAAGATGAGGCTAGATATTTTTTCCAGCAGCTTATCTCTGGTGTTAGCTATTGTCATTCTCTGCAAATATGCCATCGAGATTTGAAGCTCGAGAACACCTTGCTTGATGGCAGCCCCGCTCCGCGCCTCAAAATCTGTGATTTCGGTTATTCTAAGTCATCTTTGCTGCATTCACGACCAAAGTCAACTGTGGGAACTCCAGCGTATATTGCACCTGAGGTTCTTTCTCGAAGAGAGTATGACGGCAAGTTGGCAGATGTATGGTCTTGTGGGGTGACCCTATATGTTATGCTGGTGGGAGCATATCCATTTGAGGACCAAGAAGATCCAAAGAATTTCAGAAAAACCATTAATCGAATAATGGCTGTTCAGTACAAGATTCCTGACTATGTTCACATATCTCAAGATTGTAGGCATCTCCTCTCTCGCATATTTGTTGCAAATCCAGCAAGGAGGATCACCATTAAAGAAATCAAGAACCACCCATGGTTTTTGAAGAACTTGCCGAGAGAGCTCACAGAAGCAGCTCAAACCATGTACTACAGAAAAGAGAACCCAACCTTTTCCCTCCAAAGTGTCGAAGACATCATGAAGATAGTGGAAGAAGCCAAAAATCCTCCCCCAGTTTCCCGGTCGGTTGGAGGCTTTGGCTGGGGAGGAGAAGAAGATGGTGATGCAAAGGATGAGGTTGAGGAGGGCGAGGATGAAGAAGATGAGTATGAAAAGAGAGTCAAAGAAGCACATCAAAGTGGGGAAGTACGTGTTGTCTGA

>FvSnRK2.2

ATGGAGGAGAGGTATGAGCCAATGAAGGATCTTGGGTCTGGCAACTTTGGAGTGGCGAGGCTGGTCAGGGATAAGAAGACCAGGGAGCTTGTGGCTGTGAAGTACATAGAGAGAGGCAAGAAGATTGATGAGAATGTTCAGAGGGAAATTATTAATCACAGATCCTTGAGGCATCCTAATATTGTCAGGTTCAAAGAGGTCCTGTTGACTCCAAGTCATCTAGCTATTGTCATGGAATATGCAGCTGGTGGTGAACTCTTTGAGAGGATATGTAGTGCTGGTAGATTTAGTGAAGATGAGGCAAGATTTTTCTTCCAGCAGCTGATATCTGGAGTCAGCTACTGTCATTCCATGGAAATCTGTCACAGGGATCTGAAGCTGGAAAACACACTCTTGGATGGAAGTGCGACACCACGTCTGAAAATTTGTGACTTTGGATACTCCAAGTCTGCTATTTTGCATTCGCAACCAAAATCAACTGTTGGAACACCTGCTTACATTGCTCCGGAGGTTCTGTCACGGAAGGAATATGATGGAAAGATTGCAGATGTTTGGTCCTGTGGTGTGACACTATATGTCATGTTGGTAGGAGCATATCCTTTTGAGGATCCCGAAGACCCTCGAAATTTTCGTAAGACCATTGAGAGAATTATGAGTGTCCAGTACACCATACCCGATTATGTTCGTATTTCAGCAGACTGCAAGCACCTACTGTCTCGTATTTTTGTTGCCAACCCATCTAAGAGGCTCAGTCTTCCTGAGATAAAACAGCACCCTTGGTTTCTGAAAAATTTGGCAAAAGAGCTAATTGAGGTTGAGAAAAAAAGCTTTGCGGAAGTGGAACGTGACAACCCGACACAGAACATTGAAGAAATAAATAAGATCATACAAGATGCAAGGACACCAGGGGAAGGTTCCAAAGGAGTTGGCCAAGCTGTTGCAGGAGCAGGACCATCCGACTCTGATGATGTAGATTTGGACTCAGAAGTTGATCTTAGTGGTGACTTTGCTTGA

>FvSnRK2.3

ATGGATCGGTCTATGCTGACAGTTGGGCCGGGTATGGATATGCCGATCATGCACGATAGTGATCGGTACGAGCTGGTTAAGGATATCGGGTCGGGTAATTTTGGGGTGGCCAGGCTCATGAGGGACAAGCAGACTGAGGAGCTTGTTGCTGTTAAGTACATAGAGAGAGGTGAGAAGATAGATGAAAATGTACAAAGGGAAATTATAAACCACAGGTCATTGAGGCATCCTAACATTGTCCGATTTAAAGAGGTAATATTGACACCGACTCATCTGGCTATTGTGATGGAATATGCATCTGGAGGAGAGCTCTTTGAGCGGATATGCAATGCAGGGAGGTTCAGTGAGGATGAGGCACGTTTCTTCTTTCAGCAACTTATATCAGGAGTCAGTTACTGTCACGCAATGCAAGTGTGCCATCGGGACTTGAAGTTGGAGAACACATTGTTAGATGGAAGTCCTGCTCCTCGTTTAAAAATATGCGACTTTGGCTATTCAAAGTCTTCTGTGCTACACTCGCAACCAAAATCAACTGTTGGTACCCCTGCATATATTGCTCCCGAGGTGTTACTTAAGAAAGAATATGATGGCAAGATTGCAGATGTCTGGTCTTGTGGGGTGACCTTATATGTCATGTTGGTGGGTGCATACCCATTTGAGGATCCAGGGGAGCCTAAGAACTTCCGCAAGACAATACATCGTATAACGAGTGTCCAGTACTCAATTCCCGACTATGTTCATATATCTCCAGAGTGCCGTCATCTGATCTCAAGAATTTTTGTGGCTGAACCGGAGAAGAGGATAACAATTCCTGAAATAAGGAACCACGAATGGTTTCTAAGGAACCTTCCAGCAGATCTCATGGTTGAAAACACCATGAACAGCCAGTTTGAAGAGCCCGATCAACCCATGCAAAGCATAGATGAGATCATGCAAATTATTGCGGAAGCTACAATACCGGCTGCAGGGACTAACAATCTCAACCAGTATCTTGCTGGCAGCCTGGACATCGACAACATGGAGGAGGATCTTGATACTGATCCTGACGACCTTGACATCGACAGTAGCGGGGAAATAGTGTATGCAATTTGA

>FvSnRK2.4

ATGGAGAAGTATGAGTTTGTCGAGGATATAGGATCAGGCCATTTCGGCGTCGCCAAGCTCATGAGAAACAAAGAAACCAAAGAGCTCGTCGCCGTGAAATACATCGACCGCGGCCTCAAGATTGATCAGAATGTTGCCAGAGAGATCATCAACCACAGATCACTTTGCCATCCAAACATCATTCAGTTCAGAGAGGTGTTTTTGACTCCTACGCATCTTGCTATTGTTATGGAGTACGCCGCAGGCGGAGAGCTATTTGACCGAGTTGCCAATGCTGGAAGATTGAGCGAAGACGAGGCTAGATTTTTTTTCCGGCAGTTTATCTCAGGTGTAAGCCATTGTCATTCTATGCAAATATGCCATCGAGATTTGAAGCTGGATAATACTCTGCTGGACAGGACGAGCTCGTCTCCACGCCTGAAAATATGTGATTTCGGTTTTTCCAAGTCATCTTTGTTGCATTCAAGAACAAAGTCAATTGTGGGAAGTCCGGCGTATACTGCACCTGAGGTCTTTTCTCGAAAAGAGTATGACGGCAAGTTGGCGGATGTATGGTCTTGTGGGGTGAGCCTATATGTTATGGTGGTGGGAGCATATCCATTTGCAGACGAAAACGATCAGACGAATATCAGAGAAATCGTTAAACGAATAATGTCTGTTCGATACGTGATCCCCGGCTATGTTTACATATCTGAAGATTGTAGGCATCTCCTCTCTCGCATCTTTGTTGCAGATCCAGCAAGGAGGATCACCATTCAAGAAATCAAGAACCACCCGTGGTTTTTGAAGAACTTGCCGAGAGAGCTCACAGAAGAAGCTCAAACCAGAAAAGAAAACTCGACATGTTCCCTTCAAAGTGTTGAAGACATAATGAAGATAGTGGAAGAAGCCAAAAACATTCCTCCCCCAGTTTCCCGATCGGTTGGAGGCTCTGGTGATGCAGAGAAGACGAGTATGAAAAGAGAGTCAAAGAAGCACATCAAAGTGGGAAAAGAGATAGAGGAGGGCGATCTCAAGAAGACGAGTATGAAGAGAGAGTCGAAGAAGCACATCAAAGTGGGGAGGTACGGGGTGTGTCGGATGAGCAGCACCTGA

>FvSnRK2.5

ATGGAGAGGTATGAGATAGTGAAAGATATTGGTTCTGGGAACTTTGGTGTTGCAAAATTGGTGAAGGACAAATGGAGTGGTGAGCTCTATGCCATCAAGTTCATTGAGAGAGGCCAGAAGATTGATGAGCATGTACAGAGAGAGATCATGAACCACAGGTCACTGAAGCATCCAAATATCATAAGATTTAAGGAGGTCTTGTTAACACAAACCGATTTAGCGATTGTCATGGAATATGCAGCAGGAGGAGAACTTTTTGAAAGAATATGCAATGCTGGTAGATTCAGTGAAGATGAGGCCAGATTTTTCTTCCAGCAGCTGATTTCAGGAGTCAGTTACTGTCATTCAATGCAAATCTGTCACAGAGATCTTAAACTGGAGAACACACTTCTAGATTCAAGCTCAGCTCCACGTCTCAAAATATGTGATTTCGGTTATTCCAAGTCATCTGTGCTGCATTCACAACCCAAATCAACTGTAGGAACACCTGCCTATATTGCACCAGAAGTCCTGTCAAAAAAAGAGTATGATGGGAAGATAGCAGATGTTTGGTCTTGTGGGGTTACCCTATATGTGATGCTGGTTGGTGCTTATCCTTTTGAAGATCCAGAAGATCCCAGAAATTTCAGGAAGACACTTCAGCGAATTCTCAGCGTCAGCTATTCAATTCCTGACTATGTACGCGTTTCCAGGGAATGTACACATCTTCTTTCTCGAATTTTCGTTGCTAACCCCGAAAAGAGAATTACAATCCCAGAGATAAAGCAGCACCCCTGGTTTCTGAAAAATTTACCATCGGAATTCATGGATGAAGATGACATGCAGATTGGTGAGGTTCAGAAAAATGAGATTTCACAAAGTGTTGAAGATATAGTGTCCATTATTCAAGAGGCTCGAAAACTTGGTGATGGCATCAAAGTTGGACATTTTCTTGGGAGCATGGACCTTGATGAGATAGATGATGCCGACATCGATGATATAGAAACTAGTGGTGATTTTGTATGTGCGTTGTGA

>FvSnRK2.6

ATGGAGAGGTATGAGATTGTGAAAGATATTGGGTCTGGGAATTTTGGGGTAGCTAGGTTGGTCAGAGATAAGTTGACCAGAGAACTCTTTGCTGTTAAGTTCATTGAGAGAGGCCAGAAGATAGATGAACATGTGCAAAGGGAAATCATGAACCACAGATCATTGAAGCATTCCAATATTGTTCAATTCAAAGAGGTTCTTCTGACACCAACTCATTTAGCCATAGTAATGGAGTATGCTGCTGGAGGAGAACTCTTTGGGAGAATTTGTAATGCTGGTAGATTTAGTGAGAATGAGGCAAGGTTTTTCTTCCAGCAACTGATATCAGGAGTTAGTTACTGTCATTCAATGCAAATATGTCACAGAGACCTTAAGCTTGAAAATACACTCTTAGATGGCAGCACAGCACCTCGTGTCAAAATATGTGATTTTGGATACTCAAAGTCACTACTGCAGTCTCAGCCAAAATCAACTGTAGGAACACCAGCCTATATTGCTCCTGAGGTCCTATCTAAAAAGCAATATGATGGAAAAATTGCAGATGTTTGGTCTTGTGGAGTCACCTTATTTGTGATGATAGCCGGAGCATATCCTTTTGAAGATCCTGATGACCCTAAGAACTTCAGAAAAACCATTAATCGGATCCTTGCCGTACACTACTCAATACCAGATCATGTTCAAGTTTCCATCGAATGTAGACATCTTTTATCTCAGATATTTGTGGAAAACCCCGAAAAGAGAATTACAATCGCAGGAATTAAAAGCCACCCTTGGTTCTTAAAGAACTTACCTATGGAGCTGATGGAAGGAGGAAGTTGGCAGAGCAATGATGTAAATATTCCATCCCAAAGCACTGAAGAAGTTCTGTCCATAATTCAAGAGGCGAGAAAATCAGTACCGTACCCAAACACAGTTACGCATCTCATTGAAGGCAACATGGATCTTGATGATTTGGATGCTGAAGTTGAAGATGTTGAAACAAGTGGTGATTTTGTGTGCCACTTGAGTAGTTAA

>FvSnRK2.7

ATGGAGAAGTATGAGGTTGTGAAAGATATTGGGTCTGGGAATTTTGGTGTAGCAAGGCTGGTCAGAGACAAGAGTACAAACGAACTCTTAGCTGTTAAGTTCATTGAGAGAGGCATCAAGATAGATGAAAATGTGCAAAGGGAGATCATGAACCACAGATCCTTGAAGCATCCCAATATTGTTCAATTCAAAGAGATATTGCTGACACCAACTCATCTAGCAATTGTGATGGAGTATGCTGCTGGAGGAGAACTTTATGACAGAATAGTCAAGGCCACAAGGTTTAGTGAGAATGAGGCAAGGTTTTTCTTCCAGCAATTGATATCAGGAGTTAGTTACTGTCATACAATGCAAATATGTCACAGAGATCTTAAGCTTGAAAATACACTCTTAGATGACAGCGCAGCACCTCGTGTGAAAATATGTGATTTTGGATACTCAAAGTCATTACTGAGTTCTCTGCCAAAGTCTACTGTGGGAACACCTGCTTATATTGCACCTGAAGTTCTATCTAAAAAACAATATAATGGACAGGTTGCAGATGTTTGGTCTTGTGGAGTCACATTATATGTCATGCTTGTCGGAGCATATCCCTTTGAAGATCCTAAGGACCCTATGAACTTTAGAAAAACAATTCGGAGGATCCTTACTGTACGCTACGCGATCCCAGATAGTGTGCGAGTTTCAGTGGAATGTAGACACCTCTTGTCAAAGATTTTTGTTGCAAACCCGGAAAAGAGAATAACAATTCCAGATCTTAAGATGCACCGTTGGTTTGCAAAGAACTTACCTCTGGAAATGAAGGAAGGAGGAAGTTGGGAGAACAATGCTGTAAACACTCCATCCCAAAGCATTGAAGAAGTTCAGTCCATAATAAAAGAGGCAAGAAAACCTTTAAAGGTCCCAACTGTCAGTAGGCATTGCGTTGGAAGCAGCATGGCTATTGATGATGCTGAAGAATGA

>FvSnRK2.8

ATGCGCCAAGAACCCAGCCTTGTGCAAAATGTGCAACAGTGGAGCTGCGACACCATGATTAGTAGCGGCACCCCCACGGCCCCACCAGATATGAGGTATGAGATTTTGAAGGATATCGGAGCTGGGAATTTTGCGGTCACAAAGTTGGTCAGAGAGAAGTGTAGCGGAGAACTATATGCTGTCAAGTTGATTGAGAGAGGCAAGAAGATAGATGAAAATGTGCAAAGGGAAATAATGAACCACAGATCCTTGAAGCACCCCAATATTGTTGAATTCAAAGAGGTCCTGCTGACGCCAACTCATCTAGGTATAGTAATGGAGTATGCTGCAGGAGGAGAACTGTATGAAAGAATATGCAAGGCTGGTAGATTTAGTGAGGATGAGGCAAGGTTTTTCTTCCAGCAATTGATATCAGGACTTAAGTACTGTCATACAATGCATATATGTCATAGAGATCTTAAGCTTGAAAATTCACTCTTAGATGGTGGCACAACACCACGTGTGAAAATTTGCGATTTCGGATACTCAAAGTCGCTACTTCAATCTCAACCAAAATCTGTTGTGGGAACACCAAATTATATTGCACCTGAAGTCCTATCTAGAAAAAAATATGATGGAAAGATTTCAGATGTCTGGTCTTGTGGAGTCACCTTATTTGTCATGATTGTTGGGGCATATCCCTTTGAAGATCCTCAGGATCCTATAAACTTTACAAAAACAATTCGGAATGTTGCAGTTTCCCGGGAATGTAGACACCTGTTTTCTAAAATATTTGTGGCAAACCCAGAAAAGAGAATAACAATCCCTGAAATCAAGAGCCATCCTTGGTTTGTAAAGAACTTACCTATGGAAATGATGGAAGGAGGAAGTTGGGAAAGCAATGAGATGAACAATCCAGTCCAGAGTGTTGAAGAAGTTCAGTCTATAATACAAGAGGCAAGAAGACCTTTAAAAGTCCGAACTGTCAGTAGGCATCTCACTGGTAGCAGCATGGCTCTTGATGAAGCTGATGTTGGAGGTCCTGAAACAAGTGGTGATTTTGTGTGCTAA

>FvSnRK2.9

ATGGATCGGGCGGCGGTGACGGTGGGGCCGGCGATGGACATGCCGATCATGCACGACAGCGACAGGTACGATTTCGTGAGGGACATCGGGTCCGGGAACTTCGGGGTGGCGAGGCTGATGACGGACAAGCAGACCAAGGAGCTGGTTGCCGTCAAGTATATCGAGCGCGGCAACAAGATTGATGAAAATGTTCAAAGAGAGATTATTAATCATAGGTCGTTGAGGCACCCCAACATTGTTAGGTTCAAAGAGGTTATTCTGACTCCTACTCATCTGGCCATTGTAATGGAATATGCTTCTGGAGGAGAGCTTTTCGAGCGGATATGCAATTCAGGGCGCTTCAGTGAGGATGAGGCTCGCTTCTTTTTCCAACAACTTATATCTGGGGTCAGTTACTGTCATGCAATGCAAGTATGTCACCGGGACTTGAAGTTGGAAAACACTTTGTTGGATGGAAGTCCTGCTCCTCGCTTGAAAATATGTGATTTTGGGTACTCAAAATCTTCAGTGCTTCATTCACAACCAAAATCTACAGTTGGAACTCCTGCATACATTGCTCCAGAAGTACTATTGAGGCAAGAGTATGATGGAAAGGTCTTGTCAATATGGAATCCTGTGTTAGGGCATATACATGTAGTCCTCGTTTTGACCTTTTCCATGAAGCAGCAGCTCTCAGAATTGAAGACAGTGATCTCCTCTGATAGGCAAAATCTTTTTACTACTGCAGATGTATGGTCATGTGGGGTAACGTTATATGTGATGTTGATGGGATCATATCCTTTTGAGGATCCTGATGAACCAAAAGATTTCCGGAAGACTATACAAAGAATTCTTAATGTCCAGTATGCAATTCCAGATTCTGTTCCATTATCTTGTGAATGTCTGGAACTGATATCAAGAATTTTCGTCTCAGACCCTACTGCGAGAATTACAATTCCTGAAATAAAGAACCACCCATGGTTCTTGAAGAATCTCCCCGCAGATTTGATGGATGAGATGACAATGGGCAACCACTTCGAAGAGCCTGATCAACCGATGCAGAGCATTGATACAATCATGCAAATAATTGCTGAGGCCACCATACCAGCAGTTGGAATCCACAATCTTAGCCCATTCATGAATGACAGTTTTGACATGGACGATGATATGGATGACTTAGATTCAGAATCTGAACTAGATGTTGATAGCAGTGGGGAAATAGTGTATGCTATTTAA

>FvSnRK3.1

ATGGTGTTGAAGCTTGAAATGGATAATAAGTCTTATATATTGATGCAAAGGTACGAGTTAGGGAGACAACTGGGTAAAGGCACTTTTGCCAAGGTTTACTATGCAAGGAGCTTGATAACAAATCAGGCTGTGGCGATCAAGGTTGTTGACAAAGAGAAGATTATGAAGGTAGGGCTGATGGATCAGATAAAGCGTGAGATATCTGTTATGAGACTGGTTAGACATCCCAATATTATACACCTTTATGAGGTCTTGGCAACCAAAACTAAGATATACTTTGTCATTGAGTATGCTAAAGGTGGTGAGCTATTTAACAAGGTTGCTAAAGGAAAGCTGAAGGAGGATGTCGCACGGAAGTATTTCCAGCAGCTGATCAATGCTCTTGATTTCTGTCATAGCAGGGGAGTTTATCACCGGGATATTAAGCCAGAGAACTTGTTGCTAGATGAGAATGATAATTTGAAGATATCTGATTTTGGGTTAAGTGCCCTTGCAGAAAGCAAGCGCCAAGATGGTCTGCTTCATACCACTTGTGGTACTCCTGCCTATGTTGCTCCAGAAGTCATTAATCGGAAAGGCTATGATGGTGTGAAAGCTGATGTTTGGTCTTGTGGGGTGGTCTTGTATGTCTTATTGGCTGGTTATCTCCCGTTTCATGATTCAAATTTGATGGAGATGTACAGGAAAATCGGTAAAGCAGAGTTCAGATGCCCTAATTGGTTCTCACCAGAAGCACGTAGGCTATTGTGCAAGATGTTGGATCCAAATCCCAACACTAGAATTACCTTGGCGAAAGTTAGGGAAAGTTCTTGGTTCAGAAGAGGACCAAAATCCAAAGAGAAAGAGGTGGCTCCGGCGGCAACAGAAGCTTCCAGTCCCAGTGAGAATGAGAATAATTTGGCTGTTGAGGCAAACCAAGAGTCAGGGAGACCTTCAAACTTGAATGCTTTTGATATTATATCCCTATCTGATGGGTTTGATCTGTCCGGCTTGTTCGAGAAAAATTCTCTAAGTAGGGAAGCAAGATTCACTTCAAGAAAGCCTGCCACAGTCATCATCTCCAAGCTAGAAGAAATGGCCAAGTATCTGAAGCTAAAAGTGAATAAGAAGGATCATGGATTGTTGAAAATGGATAGACTGCAGGAAGGCAGAAAGGGTTTTTTGTCCATTGATGCAGAGATATTTGAGATCACTCCGAATTTTCATTTAGTAGAGGTCAAGAAATCAAATGGAGATACAATGGAATACCAGCAGATGTTGGAAGACATAAGGCCTGCTCTGCGAGATATTGTCTGGGTTTGGCAAGGGGAGCAAGAACAGACATCGCAGGTGCAGCAGCTACAAGAACAAGAAGAAGTGCAACAGCAACAACAAGAACAATTGCCTCAGAATCCGCCACAACTATAA

>FvSnRK3.2

ATGGAAAACAAAGGGAGTGTGCTGATGCAGCGATACGAACTGGGGAGGTTATTAGGCCAAGGAACCTTTGCCAAGGTCTACCATGCTAGGAACCTCAAAACCAACATGAGTGTGGCCATAAAGATAATTGACAAAGAGAGGATCTTGAAGGTCGGGATGATTGATCAGATCAAGCGTGAAATTTCTGTGATGCGGTTGATTAGACATCCAAATGTGGTGGAGCTTTATGAGGTGATGGCCAGCAAAACCAAGATTTACTTTGTCATGGAGTATGTCAAAGGTGGTGAGCTCTTCGACAAGGTCTCCAAAGGCAAGCTAAAGGAGGATGTTGCTAGGAAGTACTTTCAACAGCTCGTCAGTGCTGTTGACTACTGCCATAGTAGAGGTGTATACCATCGTGATCTGAAACCAGAAAACCTACTTTTGGATGAGAATGGAAATCTAAAGGTTTCAGATTTTGGACTGAGTGCCCTTGGTGAATCCAAGCGCCAAGATGGATTGCTTCATACAACCTGTGGGACTCCTGCATATGTTGCCCCAGAAGTAATAAACAGGAAAGGCTATGATGGTGCCAAAGCTGACATTTGGTCATGTGGGGTGGTATTGTTTGTTCTATTGGCTGGCTATCTTCCATTTCATGATGCAAATCTGATGGAGTTGTATAGGAAGATTGGTAAGGGGGAATTCAAATTCCCTAACTGGTTTACTCCTGAAGTACGCAGGTTGCTGTCAAAGATATTTGACCCAAATCCAAATACTCGGATATCTTTGTCTAAAGTAATGCAATCTTCTTGGTTCCGAAAGGGGCTGGTCCAGAAACCTGCAATTGTTGAATTACCAGTGAAAGAGCTTGCCCCTCTGGATGCTGATTCCATTTTTGGACCTGGTGAAGATAACAATTCTGTGACAGAGGCAAAGCAAGAATTAGTGCAGAAGCCGTCTAACTTGAACGCTTTCGATATCATCTCCTACTCCGCTGGCTTTGATTTGTCTGGACTGTTTGAAGAGGCTGAACAGAAAAAAGAAGTGCGGTTTACATCCAACAAAACTGCTTCAACCATCATCAGCAAGCTGGAAGACATAGCCAAGCGTCTGAAACTCAAAATCAAGAAGAAAGATGGAGGGTTGTTAAGAATGGAAGGGTCCACGGAAGGCAGGAAAGGGGTTTTGGGCATTGAAACTGAGATCTTCGAGATCACCCCGTCTTTTCACTTGGTGGAGGTGAAGAAGTCTAGTGGAGATACATTAGAGTATCAGAAGGTCATGAAAAAGGAGGTAAGACCAGGTCTCAAGGACATTATTTGGACTTGGCAAGGGGAGCAACAGCCGCAGCAACAAGAGCCACCACAACCAGAGCAGCAAGAGCAACAACCTTTGACGCTCCCAGTTCAAGTAGCCTCTCCCCAGGAGGCATAA

>FvSnRK3.3

ATGGCTGAGCTGCGCCCCCAAAACGGCGCCGTCTCTACCCCCACCACAACCTTCACCTCGAACACCTCCAAGACCAAGAACAACCCTCTCCTCCTCGGCCGCTTCGAAATCGGGAAGCTCCTCGGCCACGGCACCTTCGCCAAGGTGTACCACGCCCGCAACATCAAGACCGACCAAGGCGTCGCCATCAAGGTCATCGACAAAGAAAAGATCCTCAAAGGCGGCCTCATCGCCCACATCAAGCGCGAGATCTCCATCCTCCGCCGCGTCCGCCACCCCAACATCGTCCAGCTCTTCGAGGTCATGGCCACCAAAGCCAAGATCTACTTCGTCATGGAGTACGTCCGCGGCGGCGAGCTCTTCAACAAGGTCGCCAAGGGCCGCCTGAAAGAAGAAGTCGCTAGAAAATACTTCCAGCAGCTCATCTCCGCCGTCGGGTTCTGCCACGCCAGAGGCGTCTACCACCGCGACTTGAAGCCGGAGAATTTACTCCTCGACGAGAATGGTGATCTGAAAGTCTCCGATTTCGGGCTCAGTGCGGTTTCGGATGAAATCCGGCAGGACGGGCTGTTCCACACGTTTTGCGGCACCCCGGCGTACGTGGCGCCGGAGGTGCTGGGCCGGAAGGGCTACGACGCCGCGAAGGTGGATATATGGTCTTGTGGAATTGTTTTGTTTGTGCTCATGGCGGGGTACTTGCCTTTTCATGACCACAATGTTATGGCCATGTATAAGAAGATTTATAAGGGAGAGTTTCGGTGTCCGAGATGGTTCAGCTCCGAGCTTGTGAAGTTGCTGACTCGGCTTTTGGATACGAATCCGAATACGAGGATTACCATTGCGGAGGTGATGGAGAATCGGTGGTTTAAGAAGGGGTTTAAGCACATTAAGTTTTATATAGACCATGATGACAGGTTGTGTAATGTTCATGAGGATGATGGGGATGATAGTGATGCTAGTTCGGTGATGTCTGATATGTCAGAATCCGAGGCTGAATTCGAGACCAGGAGGAAGCTTACGACTTTGCCGAGACCGGCCAGTTTGAATGCGTTTGATATCATTTCGTTTTCGCCCGGGTTTGATTTGTCCGGCTTGTTTGAGGAGCGCGGGGAGGAGGCTAGGTTTGTGTCGGGTGCTCCGGTTGATAAGATTATATCAAAGTTGGAGGAGATTGCCAAAGTGGTGAGCTTTTCGGTGAGGAAGAAGGATTGCAGGGTGAGTTTGGAAGGGTCTAGGGAGGGTGTGAAGGGGCCATTGACGATTGCGGCTGAGATATTCGAGTTGACGCCCTCGTTGGTGGTGCTTGAAGTGAAGAAGAAAGCAGGGGACAAAGTAGAGTATGATCAATTTTGTAATACGGAGTTGAGACCGGGGTTGCAGAATTTGATGATTGAAGAATCTGCTGGAGGTTCTCTTGCTTCGGGAGGTTCGGTTGTTTCAGAAGGTTCTCTTGCTTCAGGTGGTTCAGTTGTTTCAGTAGGTTCTCCGTCGTCAGTTCATCATCTACCCTCTGATACTGAATAA

>FvSnRK3.4

ATGAATCAACCAAAAATCAAGCGTAGAGTGGGTAAATATGAGGTGGGAAGGACAATTGGCGAGGGAACATTTGCAAAAGTCAAGTTTGCTAGAAATTCTGAGACTGGAGAACCTGTGGCTCTTAAGATTCTTGACAAGGAGAAGGTTCTCAAACACAAAATGGCTGAACAGATCAAGCGGGAAATAGCAACAATGAAGCTGATTAAGCATCCAAATGTTGTTCAGTTGTATGAGGTCATGGGGAGCAAGACGAAGATATTTATAGTGATGGAGTTTGTTACTGGGGGAGAGCTCTTTGATAAAATTTCATTTCTGTACTTGGATGTCTTATTCTTGTTAGATTCAAGCTCTTCTGATTATTTGGTAGATCTTGCATATCTTCTCACAAAAACTTATTTTGCTTTGAAGGTAAACAATGGTCGGATGAGAGAGGATGAAGCACGTAGATATTTCCAACAGCTTATTAATGCAGTTGATTACTGCCATAGCAGAGGTGTCTATCACAGAGACCTGAAGCCAGAAAATTTGCTATTGGATGCCTATGGGAACCTTAAAGTTTCTGATTTTGGATTGAGCGCACTATCTCAACAAGTCAGGGATGATGGCTTACTTCACACTACCTGTGGAACTCCAAATTACGTTGCTCCTGAGGTCCTTAATGATAGAGGCTATGATGGAGCAACTGCGGACTTGTGGTCATGTGGAGTGATACTCTTTGTATTACTTGCAGGTTACTTGCCGTTTGATGATTCCAATCTTATTAACCTCTATAGAAAGATCTCAGCAGGTGAATTTACTTGTCCCCCTTGGCTGTCGTTTGGTGCCATGAAACTAATAGCTCGAATTCTGGATCCCAACCCTATGACACGTATCACTATTTGTGAGATTCTGGAAGATGAATGGTTCAAGAAAGATTACAAGTCACTTATGTTTGAGGAGAAAGAAGATACAAACTTGGATGATGTAGAAGCTGTTTTCAAGGATTCAGAAGAGCACCATGTAACAGAGAAGAAGGAAGAACAACCAACAGCTATGAATGCTTTTGAGTTAATTTCAATGTCAAAGGGGCTGAACCTTGGGAATTTGTTTGATGTAGAACAGGGTTTTAAGAGAGAAACAAGATTCACATCTAGATGCCCTGCAAACGAGATAATTCATAAAATTGAGGAAGCTGCAAAGCCCCTTGGTTTTGACGTACAGAAGAAAAATTACAAGTTGAGGCTAGAAAACATGAAAGCTGGGAGAAAGGGAAACCTTAATGTTGCAACAGAGATATTTCAAGTTGCACCTTCTCTTCATATGGTTGAGGTGAGAAAAGCCAAAGGGGATACGTTGGAGTTCCACAAGTTTTATAAGAACCTCTCAACCTGCTTGGAGGACGTTGTTTGGAAGACTGAGGAGGACATGCTAGAATGA

>FvSnRK3.5

ATGATTGGCTTCCGGTTAAGGCAGTACAAAAAATCATATGTTGGGAAACTTGAGCCGTGTGATCTTGATCATAGGAGGATGAGTGCTCCCAAGTCGCCGAGGATGAGGACCCGGGTCGGCAAGTACGAGCTGGGTAAGACTCTCGGGGAGGGTACCTTTGCCAAGGTCAAGTTCGCCAAGAACACCGAAACAGGGCAGTGCGTGGCCATCAAAATCCTTGATCGTGAGCAAGTCCTCAAGCACAAGATGGTTGAGCATATAAAAAGAGAGATATCGACGATGAAGCTGATCAAACACCCGAATGTGACGCAAATGTTTGAGGTTATGGCAAGCAAAACTAAGATCTACATTGTTCTCGAGTTTGTTGATGGGGGTGAGCTCTTCGACGAAATAGCCAAAAATGGGAGACTGAAAGAGGACAATGCCAGGAGATACTTCCAGCAGCTCATTAATGCCGTGGATTACTGTCATAGTAGAGGCGTGTACCATAGGGATTTAAAGCCAGAGAATCTTCTCTTAGATTCATTCGGTGTCCTTAAAATTTCAGATTTTGGATTGAGTACATTTGAACAACAAGTGCGGGAAGATGGGCTGCTTCATACTGCCTGTGGGACTCCAAATTATGTTGCTCCTGAGGTTCTCAATAATAAAGGATACGAGGGTAAATCATCTGATGTTTGGTCTTGCGGGGTGATCCTTTTTGTACTTATGGCTGGTTACCTGCCTTTTGATGAACCAAATCTAATAGCTTTGTACCGAAAAATATGCAAAGCTGAATTTTCATGTCCAGCATGGTTCTCATCTGGTGCAAAGAAACTGATACACCGTATACTTGATCCAAACCCTGCTACAAGGATGACAATTCCTGAGATATTAGAAAATGATTGGTTTAAGAAAGATTACAAGCCAGCACAATTTAAAGAAGAAGATAACATAAATCTTGATGATGTGGATGCTGTTTTCAACAATTCGAAGGAAAATTTTGTAACAGAAAGGAGAGAAAAACCTACATCAATGAATGCTTTTGAGCTTATTTCTCGGTCACAGAGTTTCAATTTAGAAAATTTATTTGAGAAGCAGATGGGTCTTGTGAAACGAGAAACCCGTTTTACTTCTCAACGCCCTGCAAATGAAATCATGAGTAAGATTGAGGAAACTGCAAAGCCTTTGGGCTTTAATATTCGCAAGAAAGACTACAAGATGAAGTTGCAAGGTGATAAGCATGGAAGGAAGGGTCACCTGTCTGTAGCCACTGAGGTGTTCGAGGTGGCTCCCTCCGTGCACATGGTAGAACTCAGGAAAACTGGCGGTGACACACTAGAGTTTCACAAGGCAAGTCATCACTGA

>FvSnRK3.6

ATGGCGGCGAGGGCGGCGGGCGTTGGGAGTCGGACTCGGGTCGGGAGGTACGATCTGGGTCGGACCCTAGGGGAGGGCAATTTCGCCAAGGTCAAATTTGCGAGGAACGTCGAGACTGGTGAGAATTTCGCTATTAAGATTCTGGATAAAGAGAAGGTGCTCAAGCACAAGATGATCGGCCAGGTGATGGCTAGCAAGACAAAAATATACATCGTTTTGGAGTTTGTGACTGGTGGGGAACTATTTGACAAAATTGCAAGTAAAGGAAGATTGAAAGAAGACGAAGCAAGGAAGTATTTTCAGCAGCTTATAAATGCAGTGGATTACTGTCATAGCAGAGGTGTTTTCCATAGAGACCTGAAGCCAGAGAATTTGCTGCTGGATGTCAATGGAGTGCTTAAAGTTTCAGATTTTGGGCTCAGTGCGCTGCCTCAGCAAGTTCGAGAAGATGGCTTACTTCACACAACATGTGGGACACCAAATTATGTTGCCCCTGAGGTCATCAACAACAAAGGGTATGATGGAGCGAAGGCAGATCTATGGTCTTGTGGCGTTATTCTTTATGTCTTGATGGCTGGCTATTTGCCTTTTGAAGATTCCAATCTCATGGCATTATATAAAAAGATATTCAAGGCTGAGTTCTCGTGTCCTCCATGGTTCTCCTCAAGTGCAAAGAAGCTAATTAAGAGAATCTTGGACCCTAACCCCTTGACACGAATTACATTTGCTGAGGTCATCGAGAATGAGTGGTTCAAGAAAGGGTATAAACCACCTAGTTTTGAACAAGTTGATGTTAGTCTCGATGATGTGGATGCAATATTCAATGATCCTGGGGATTCTCAGAACTTTGTAGTTGAGAAGCGAGAGGAACGACACGTGCCCGTTACCATGAATGCCTTTGAGCTTATCTCTACATCTCAGGGCCTGAATCTTAATAGTCTTTTTGAGAAACAGATGGAACTTGTTAAACGAGAAACAAGATTCACATCCAAACGTCCTGCTAATGAGATTATTTCTAAAATTGAGGAAGCTGCAGCACCTTTGGGTTTTGGTGTGAAGAAAAATAATTTTAAGTTGAAGCTTCAAGGTGAAAAAACTGGTCGTAAAGGTCATCTATCTGTTGCAACAGAGATTTTTGAGGTGGCCCCTTCACTCTACATGGTTGAAGTCCGCAAGTCAGGGGGAGACACCCTGGAATTTCACAATTTCTATAAGAACCTTTCAACGGGGCTGAAGGATATTGTCTGGAAGTCAGGAGATGATGCAAGGAAGGAGGCAGAATTTGGTTCCGCTTCTAGTTCTGGTACTGCCGCTGGTGCTGGTGCTGTGTCATCTACATGA

>FvSnRK3.7

ATGGCTAATGAGAAGAGCGCCGGTTCCGCCTTGCTTCACGGAAAGTACGAGCTGGGCCGGATGCTGGGACATGGAACCTTCGCGAAGGTGTACCATGCCCGGAACTTGAAGACGGGAAAGTCCATGGCGATGAAGGTGGTGGGGAAAGAGAAGGTGATCAAGGTCGGAATGATGGAGCAGATTAAGAGAGAAATCTCGGTGATGAGGATGGTGAGGCACCCCAACATCGTCGAGCTTCATGAGGTCATGGCAAGCAAGTCCAAGATCTACTTCGCCATGGATCTCGTCCGCGGCGGCGAGCTTTTCGCGAAGATAGCGAAAGGAAGACTGAAGGAGGACGTGGCCAGAGTCTATTTCCAGCAGCTGATCTCCGCCGTGGATTTCTGCCACAGCCGAGGAGTCTACCACCGCGATCTGAAGCCGGAGAATCTCCTGTTAGATGAAGACGGTGACTTAAAGGTCACTGATTTCGGGTTGAGTGCTTTCTCGGAGCACTTGAAGCAAGACGGGCTGTTGCACACCACTTGCGGCACGCCGGCTTACGTGGCGCCGGAGGTGATCGGGAAAAAAGGCTACGACGGAGCTAAGGCGGATCTTTGGTCGTGTGGAGTTATCCTCTATGTCCTGCTCGCCGGGTTTCTTCCGTTTCAGGATGACAACTTGGTCTCCATGTATAGGAAGATTTACAAAGGTGACTTCAAATGTCCGCCGTGGTTTTCTTCCGAGGCGAGAAGACTAATCACGAAGCTTCTGGACCCGAACCCGAGTACCAGAATCTCCATTGCGAAGATCATGGATTCGTCTTGGTTCAAAAAATCTATCCCGAAAACGGTGAAGTCTAAGAAAGAGCGAGAGTTCGATGAGACTACGGAGAAGACTTCGAAGCAGATGGAGACATTGAACGCGTTTCATATCATTTCGCTCTCTGAAGGGTTCGATTTGTCTCCTCTGTTTGAGGAGAAGAAGAGGGAGGAGAGAGAGGAGCTGAGATTCGCGACGACGCGGTCGGCGAGCAGTGTCATATCGAAGCTGGAGGAGGTGGGGAAAGCCGGCAAGTTTAAGGTGAAGAAGAGTGACTCCATGGTGAGGCTGCAGGGCGAGGCGAGCGGCAGGAAAGGGAAGTTGGCTATTGCGGCGGAGATTTTCGCTGTGACGCCGTCGTTTCTGGTTGTGGAGGTGAAGAAAGACAATGGTGATACTCTGGAGTATAATCAGTTCTGTAGTAAAGAGCTCAGACCGGCGCTCAAAGACATTGTCTGGACCAATTCGGCCCCCCCTGCTTGA

>FvSnRK3.8

ATGGAGAACAAGAAAGCAAACATATTGATGCACAAGTACGAGCTGGGGCGCCTTCTCGGGAAAGGTACTTTCGCCAAGGTTTACCATGCTCGAAACTTGAGGACCGGCCAAAGTGTTGCCATTAAGATCATAGACAAAGAGAAGGTGCAACAGGTTGGATTGATTGATCAAATCAAGCGTGAAATTTCGGTCATGCGCCTTGTTAGGCACCCCAATGTTGTTCAGCTCTATGAAGTGATGGCCAGCAAGACCAAAATCTACTTTGCCATGGAGTATGTGAAAGGTGGTGAGCTCTTCAACAAGGTTGCCAAAGGGAAGCTCAAGGAAGACATAGCCCGAAAATACTTCCAACAGTTGATTGGAGCCGTTGATTACTGCCACAGCCGCGGAGTTTATCACCGTGACATCAAGCCAGAGAATCTCCTGGTTGATGAGCATGGTAACCTCAAGGTCTCAGATTTCGGGCTGAGTGCATTGATAGAGTCAAGAGGTCAAGATGGTCTGTTGCACACCACTTGCGGAACTCCTGCTTATGTAGCACCAGAAGTGATCAACAAGAAAGGTTATGATGGTGCCAAGGCAGATACATGGTCATGCGGGGTAGTCCTGTATGTTCTTTTAGCTGGTTTTCTTCCATTCCACGACACAAATCTCATGGAAATGTACAGGAAGATCAGCAGAGGAGACTTCAAGAGTCCACAATGGTTCCCTCCAGAGGTTCGTAAGCTACTTGCACGGATTCTTGATCCGAATGCCACCATGAGAATAAGCGTGGATAAGATCATGGAGAACAGTTGGTTTAAGAAGGGGTTTAAGCATATTGATGCCCCGTTACCAATTCCATGTGATCCAAGCACATCTATCAGTGATGTGCATTCTGCTTTTGGATCACCAGACAGTTCAGAAGGCAGTTCTAATAGGAAAGCGGAAACTACTAATGCAGCAAGCCCCATGAGGCCAACTAACTTCAATGCCTTCGACATCATATCTCTCTCACCGGGATTTGATCTATCTGGTTTGTTTGAGGGTGATCACAAGCATAGATCATCACAGTCACGATTCACCACTACAAAACCAGCATCTACTATTGTTTCGAAATTTGAACAGATTGCACAAATGGAGAGATTCAGATGCATGCAGAAGGACGGGACTGTCAAATTGCAGGGCAGCAGGGAAGGAAGGAAAGGGCAGCTTGGTATTGATGCTGAGATTTTCGAAGTCACACCTTCGTTTTTCGTTGTGGAGGTGAAGAAAACAGCTGGGGACACATTGGAATACATTCAATTCTATGACCATGATTTAAAGCCCTCTCTTAAGGACATAGTATGGACTTGGCAAGGAAATGATCCACAGCAGCAACACCAGCCAGCAACTCAAGTCTCTTGA

>FvSnRK3.9

ATGCCGGAGATCGAGGTCGTGTCCGACGCCGGCGACGGCGCATCGGAGGCCTCTTCGTTGGACGAGACCGGTGGCGCCCTGTTCGGGAAGTACGAGCTGGGGAAGCTCCTCGGCCGCGGCGCATTCGCCAAAGTCTACCACGCGCGTGACGTCAGCTCCGGGCAGAGCGTGGCGATCAAGGCGGTGAGCAAGCAGAAGGTGCTGAAAGGCGGCTTCACGTCGAACGTGAAGCGCGAGATCTCGATCATGCGGCGGCTGCAGCACCCCCACATCGTCAAGCTCTACGAGGTCTTGGCCACCAAGACCAAGATCTATTTCATCATGGAGTTCGCGAAAGGCGGCGAGCTTTTCGGGAAAATCTCCAAAGGTCGGTTCAGCGAGGATCTCAGCCGTCGGTACTTCCAGCAGCTGATCTCCGCCGTTGGATACTGCCACTCACGCGGAGTCTACCACCGTGATTTGAAGCCGGAGAATTTACTCTTAGACGAGAATTGGAACTTGAAAGTTTCGGATTTCGGACTCTCCGCCGTGACGGAGCAGATCCGACCAGACGGGCTTCTCCACACTCTCTGCGGCACCCCGGCTTACGTGGCGCCGGAGATTCTCGCCAAGAAAGGCTACGACGGCGCCAAGGTGGATATATGGTCGTGCGGGATCATTCTCTTTGTTCTCAACGCCGGCTATCTTCCGTTCAACGATCCCAATCTCATGGTAATGTACCGGAAAATCTACAAGGGGGAGTTTCGCTTTCCGAGGTGGACGTCGCCTGGCCTGAGACGGTTGATTTCGAGATTGCTTGACACGAACGTCGAGACGAGGATCACCGTCGACGAGATCATCAAGGATCCTTGGTTCAGTGTTGGCTACAAGGACGTCAAGTTTCACTTGGAGGATTTTAACTTGAAGGAGTGGAGGGACGAGGACAACGACACGCCGTTGAATGCTTTTGATTTGATTTCATTCTCGTCGGGGTTCGACATCTCGGGGCTGTTCCGGAAGCCGGAGATATCCGACTGCGGGGAAAGGTTTGTTTCGGCAGAGACGCCGGAGAGGATAATTCAGAAGGTGGAGGAGGTGGCATTGGCGGAGGGGATGACGGTGATGGAGAAGAAGACCTGGGGGGCAAAGTTGGCAGGGCAGAATGGTAATTTGGTAGTTGCTATTGGGATTTACCGTTTGACGGAGAAGCTGGTGGTGGTGGAGTTGAACAAGAGGGAAAGAATAGGGGAGAATTGCCAGCAGATATGGAAGGACAAGCTGAGGCCGCAGCTGTCGTGTTTGATATACAAACCGGAAGAAGAACAAGTCTCCGGTGAATAA

>FvSnRK3.10

ATGATGTGGATGTCATATCCACAAAGTAGAGCTTCACAGCTTCGTCGGAGCCCAGAGACCCCTAGAATCACCTCACTCCGGCTTCAGAAGGTTCCCCTTCGCACGTGCCTCGCTTCTCCGGCGACCGGAATATTCGCATTTCCCGCTTCGGAGGTCAATGAGAGATTAGGAAAGACGATGATGAAGAAGAAGGTGACCAGAAATGTAGGCAAGTATGAGGTTGGGCGAACGATCGGCGAAGGAACCTTCGCCAAGGTCAAGTTCGCCAGGAACGCCGAGACCGGCGAGAGTGTTGCCATGAAGGTCTTGGCTAAAAGCACCATTCTCAAGCACAGAATGGTTGATCAGATTAAGAGAGAGATTTCGATAATGAAGATTGTCAGGCATCCTAATATAGTGAGGTTGCATGAGGTTTTGGCTGGCCGGACTAAGATATATATAATTCTCGAGTTTGTAACTGGAGGAGAGTTGTTTGACAAAATTGTCCATCAAGGAAAGCTTCGTGAAAATGAATCAAGGAAATACTTTCAGCAGCTTATAGATGCGGTGTCTCATTGTCACAGCAAGGGTGTTTACCATAGAGACCTGAAGCCTGAAAATCTTCTCCTTGATGCTTATGGAAATTTGAAAGTTTCTGATTTTGGACTGAGTGCATTGCCCCAGCAAGGGGATGGTCTTCTTTTCACAACATGTGGAACCCCAAACTATGTCGCCCCTGAGGTGCTGGGCAGTAAAGGTTATGATGGTGCAGCTGCTGATGTTTGGTCGTGTGGGGTCATCCTTTATGTTCTTATGGCTGGATACCTCCCATTTGATGAGGCAAACCTTGCTGCCTTATATAAAAAGATTAACGCAGCAGAGTTCTCCTGCCCGTTTTGGTTCTCTCCAGGGGCAAATTCATTAATACATAAAATACTTGATCCCAATCCTAAAACTCGTTTTCGGATTGAAGAAATCAGAAAGGATCCATGGTTTAGAAAAAATTATGCGCCTGTTGAATATAGAGAAGATGAGGAAGTCAGTTTAGATGATGTTCGTGCAGTCTTTGAGGACATTGAGGACCAATATGCAGAAGAAAGAACAGATAATAAAGACAGCGGTCCTTTATTAATGAATGCATTTGAGATGATTACCCTCTCCCAAGGGCTAAATTTATCTGCTCTGTTTGACAGGAGACAGGATTATATAAAAAGACAAACTCGTTTTGTATCCCGCAAACCAGCTAAAGTTATAATTTCAAATGTAGAAGCTGTTGCAGAATCAATGAGTCTCAAGGTCCATACACGTAATTTCAAGACAAGACTTGAAGGGATATCTGCAAATAAGGCTGGACAATTTGCAGTTGTCCTTGAGATTTTCGAAGTTGCACCATCCCTTTTCATGGTAGATGTTCGAAAGGCAGCTGGTGATACTCTTGAATATCACAAGTTTTACAAAAATTTCTGCGCCAAGTTAGATGACATTATATGGAAACCAAAAGACGGTATGGCCAGTTCCAATATACTTAGAACGACGACTTGCTGA

>FvSnRK3.11

ATGGAGGAAAGGACGGTGTTGTTCGGCAAATACGAGACGGGAAGGTTGCTGGGCAAAGGCACCTTCGCCAAAGTGTACTACGGCAGACAAATAGAGACCAACGAAAGCGTGGCGATCAAAGTGATAAGCAAAGAGCAGGTTAAGAAGGAAGGCATGATGGAGCAGATCAAGCGCGAAATCTCCGCCACCCGATTACTCCGCCACCCAAACATCGTCCAACTCAAAGAGGTCATGGCCACCAAGACCAAAATCTTCATCGTCATGGAGTACGTCAAAGGCGGCGAGCTGTTCGCGAAAGTCGCCAAGGGAAAGCTGAAAGAAGATCAAGCCCGCAAATACTTCCAGCAATTGATCAGCGCCGTCGATTTCTGCCACAGCCGCGGAGTCTCCCACCGCGACTTGAAGCCCGAGAATCTCCTCCTGGACGAGAACGGGGACCTCAAAATCTCCGACTTCGGGCTCTCCTCGCTCCCGGAGCAGCTCCGAAACGACGGCCTTTTACACACGCAGTGTGGCACCCCTGCTTACGTGGCGCCCGAGGTCCTGAGGAAGAAAGGCTACGACGGTTCGAAAACTGATATATGGTCGTGCGGAGTGATTCTGTTCGTGTTGCTGGCGGGGTTCCTTCCGTTTCAAGACGAGAACATCATGAAGATGTACCGCAAGGTTTTCAAGGCCGAATTCGAATGCCCGCCCTGGTTCTCAACCGAGGCCAAACGCCTCGTCTCCAAGTTGTTGGTGTCCGATCCTGAGCGCCGTATCACCATCCCTGAAATCATGCGCGTGCCTTGGTTCCGCAAAGGCTACACGCGCCCCCTCGCCTTTTCGCCTCCCCCTGCTTCCTCTGACAAGTCATTTGATGAAGATTTTGGTTCTACTGCCCCTGCTCCTGCTGATGCTGCCAACCACAAGTCGCAGTCGCCCAACTTCTTCAACGCGTTCCAGTTCATCTCATCCATGTCTTCCGGATTCGATTTGTCCAATTTGTTTGAGAGCAAGAGAAAAGCAGGGACCATGTTCACCTCCAAGTGCTCCTCAGCGGCTATTATGGCCAAGATTGAGCATGCTGCCAAGGCATTGAGCTTCAAGGTGGGGACGGTTAAGGACTTCAAGTTAAGGCTACAAGGCCCAAACGAAGGCCGGAAAGGGAGGCTTTCGGTGACCGCGGAGGTCTTCGAGGTGGCGCCGGAGGTCGCGGTGGTCGAGTTTTCCAAGTCTGCCGGGGATACTTTGGAGTACGCCAAGTTTTGTGAGGAAGATGTTAGGCCAGCATTGAAAGACATTGTCTGGACCTGGCAAGGTGACGGTAACAAAGTCGATGGTGAAGAATGA

>FvSnRK3.12

ATGGTGATCATAAGCAAGGGGCAGGAGAAGAGCAGTGATCAGAGTAAGAAGGGAATGCGACTTGGGAAATACGAGTTGGGGAAGACTTTGGGTGAGGGCAATTTCGGCAAAGTCAAGTTTGCTAAGGACGTCGGCTCCGGCCAACCTTTCGCCGTTAAGATTCTTGAGAAGAAGAGAATCACTGATCTCAATATCGCCGACCAGATAAAGAGGGAGATTGGCACTTTGAAGCTTCTAAAACATCCAAACGTAGTCCGATTACATGAGGTCGTGGCAAGCAAAACCAAGATTTACATGGTCTTAGAATATGTTACTGGCGGGGAATTGTTTGATAAAATTGCACAAAAGGGAAGACTTAAAGAGTCCGAAGGTAGAAAGCTTTTTCAACAGTTAATTGATGGTGTGAGCTACTGCCACAACCAAGGTGTTTTCCATCGGGATCTTAAGCTGGAGAATATTCTTGTTGATTCCAAAGGAAACATAAAGATATCTGACTTTGGCCTTAGTGCTTTGCCGCAGCATTTTAGGGAAGATGGTTTGCTGCATACAACCTGTGGAAGCCCCAATTATGTTGCCCCTGAGATCCTTGCTAATAGAGGGTATGATGGTGGCACCTCTGATATATGGTCATGTGGTGTCATCTTATATGTCATTCTAACAGGGTATCTCCCGTTCGATGATAGGAATCTTGCAGTTCTCTATCAAAAGATCTTGAAGGGGGATGTTCAGATACCCAAATGGTTATCACCTGGAGCACAAAACTTGATAAGAAGGGTTCTCGATCCCAGTCCTCTCACCAGAATAAACATGACAGACATCAAGTCAGATGAATGGTTCAAGCAGGATTACTTTCCTGCAAAAGCTGACGAAGAAGAAGAAGATATAAACGTTGATACCGAAGCTTATTCAATAAAAGAAGTGCCATCTGAAGGGGAAAAGAGTCCAGATTTGCGGCACTCACCCACCCTTATCAATGCCTTTCAGTTGATTGGAATGTCTTCATGTCTAGACCTCTCTGGATTCTTTGAGAAAGAGGATGTGTCTGAGAGGAAGATCAGATTTACTTCCAACCACTCTGCAAAGGATTTGCTAGAGAGGATTGAAGAAATTGTAATAGAGATGGGATATGCTGTCCAGAAGAAAAATGGAAGGGCTAAAATAATGTTGCTACCTTCAACATTTCAGTTAAAGGTGATGCAAGAGAACAAGGGGCAGAGAAATCTGGGCAGTCTCTCAGTTGCAGCAGAGGTGTTTGAGTTAAGCCCAACATTACACGTAGTTGAATTAAGAAAATCATACGGAGATCCGTCTGCATATAGACAGTTGTGTAAAAAGCTATCAAATGAGTTAGGTGTTCCATCGAGCCAAGAATTGTTGGCCAGCGAGGTATTGAAGTCCAGTTCTTTGCAGAGTCAAACGGCGTAG

>FvSnRK3.13

ATGGGCCGTGGGCCCCCACCACCCACCCCTTCCTCCGCCGCCGCCGCCGCCGCCACCACGACCACCAACCTCCTCGGAAAGTATCAAATCAGCCGGATGCTGGGCCGCGGCAGCTTCGCCAAGGTCTATAAGGCCCAAACCATCGCCGACGAGACTCCCGTCGCCATTAAAATCATCGACAAGCTCAAAACCCACGCCGCGATGGAGCCTCTGATCCTCCGGGAGATCTCCGCCATGCGCCGTCTCCAGGACCACCCTAACATTCTCAAAATCCACGAGGTCATGGCCACCAAGTCCAAGATCTACATCGTCGTGGAGCTCGCCACCGGCGGCGAGCTGTTCGCCAAGATCTCACGGCACGGCAAGTTGCCGGAGTCTCTGGCCCGCCGCTACTTCCAGCAGCTCGTCTCCGCCCTCCGCTTCTGCCACGAGAACGGCGTCGCTCACCGCGACGTGAAGCCGCAGAACCTCCTCCTCGACGGCAACGGCGACCTCAAAGTCTCCGATTTCGGACTCTCCGCCCTACCGGAGCAGCTCAAAAACGGCCTCCTCCACACCGCCTGCGGGACTCCGGCGTACACGGCGCCGGAGGTGCTCTACCGGGTCGGGTACGACGGGTCCAAGGCCGACGCGTGGTCCTGCGGCGTCATCCTCTTCGTCCTCCTCGCCGGACACTTGCCGTTCGACGACAGCAACCTGGTGGCGATGCACAAGAAGATCCAACGCCGCGATTACGTCATACCGGCGGCGATTTCGAAACCGGCGCGGCGGATCATATACCAGCTCCTCGACCCGAACCCGAACACGCGGCTGAGCGTGGAGGCCGTGATGGAGAAGGCGTGGTTCCAGAAGGCCATAGATCTGAAGCTAGTCTCCGACGGCTGCGATGTCTTTGAATTGGAGAAGCCGCCGGCGAAATGCGACGTCGTTTCGGGGATGAACGCGTTCGACATAATATCCATGTCGTCGGGGCTGGACTTGTCGGGGCTTTTCGAGGCGGAGAACAGGAGCGAGAGGCGGTTCACGGCGAATGTGGCGGCGGAGAAGGTGGCGGAAAAGGTGGGGGAGGTTGGGGAGAGGATGGGGTATAAAGCGGAGAGAGGGAAAGGAGGGATGAGCGTTGGGTTGGGGAAAGGAGGGAAAGGGAAAGGACGACGTGTCGCTTTGGTGGTGGAGATGATGGAGGTGGCGGCGGGTTTGGTTTTGGGGGAGGTCAAGATGGTGGAGGGTGGTGTTGAGTTTCCTGAGCTGCTTTGGGAGGACTTGAAGACTGGGTTGGGGGACGTTGTGGTGTCTTGGCAAAACGGTGGCGTTTAG

>FvSnRK3.14

ATGCCAGAGATCGAACAACAGCAGCAGCAAGTAGTGCTCCGCATCCCAGACAATGCCCTGTTCGGGAAATACGAGCTGGGCAAGCTTCTGGGATGCGGGGCCTTCGCGAAGGTGTACCACGCAAGGAATGTGTTCACCGGCCAGAGCGTGGCCGTGAAGGTGATCAACAAAAAGAAGCTAAACGGTACCAGCCTCATGTCGAACGTGACACGCGAGATTTCCATTATGAGACGGTTACGGCACCCAAACATTGTCAAGCTTTACGAAGTCATGGCCTCCAAGACTAAGATCTACTTCATCCTTGAGTTCGTCAAAGGCGGCGAGCTTTTCGCTAAGGTCTCGAAGGGAAGATTCTCGGAGGCTCTGAGCCGGAAATATTTCCAGCAGCTGATCTCCGCCGTAGGGTACTGCCACTCTAGGGGAGTCTACCACCGTGATCTGAAGCCGGAGAATTTGCTGGTGGACGATAACGGGAATTTGAAAGTTTCGGACTTTGGTCTCAGCGCGGTGACGGGTCAGATCCGACCCGACGGGCTGCTCCACACGCTTTGCGGGACGCCGGCTTACGTGGCGCCGGAGATCTTGACGAAGAGAGGGTACGACGGAGCAAAGGTGGACATGTGGTCGTGCGGTGTGATTCTTTATGTATTGAACGCCGGGTTCTTGCCGTTTAACGACCCGAATCTGATGGCGATGTACAAGAAGATTTACAAGGGGGAGTTCCGGTGCCCCAAGTGGATGTCGCAGGATTTGAAAAGGTTCTTGGGTCGGGTCCTGGATACCAACCCGATGACCCGGATCACCGTGGACGGCGTCCTGAACGACCCGTGGTTCAGAAAAGGCGGCGAGTACAAGGAGATCAGTTTCTATGATGATGACAAACCGGACGAGGAGCAGAAATGCATGACGAATTTGAATGCTTTCGATATCATTTCTTACTCGGCGGGTCTGGATCTTTCCGGGTTGTTCGAGTCGACTAACCCGATCCAGGACTCGGAGCGGATCGTGTCGTCGGAGACGGTGGAGACGATGGTGGGGAGAGTGGAGGAGTTTGCTAAGGAGGAGAAGTTGAGGGTGAGGAGGAAGAAGGATTGGGGGATGGAAATGGAAGGGCAGAATGGTAATTTGGTGATTGGAGTGGAGGTGTCGAGGTTGACGGAGAGTTTGGTGGTTGTGGAGGCCAAGAGAACAGGCGGGGAGTCTGGGCCGTTCAATGACATGTGGAATAAGCTCAAACCCCGTCTTGTCTTAGGCCGTGAAGAAACAGTCGAAGAAGACTTGTCTTTGATCGCTTCTTCTTCTTCTTTGTCGTCTGTATAG

>FvSnRK3.15

ATGGTGGTTAGGAAAGTTGGCAAGTATGAAATTGGGAGGACGATCGGTGAAGGGACCTTTGCGAAGGTGAAGTTTGCTCAGAACACTGAGACCGGTGAGAGTGTGGCCATGAAGATCATCGATCGGAGCTCCATCATCAAGCACAAGATGGTCGACCAGGGAGTTGAGCTTGAAATGTTGATGTTTATGATGCAGATCAAGAGGGAGATATCTATAATGAAGCTTGTTCTGGCGAGTCGGACCAAGATTTATATAATATTGGAGTTCATTACTGGTGGTGAATTGTTTGATAAAATTGTTCATCACGGACGTCTTAGCGAAGGTGAAGCCAGGAGATTCTTCCAACAGCTTATTGATGGAGTGGATTATTGCCACAGCAAAGGAGTGTATCATAGAGATTTAAAGCCGGAAAATCTTTTGCTTGATTCCATCGGAAATTTAAGAATTTCAGATTTTGGACTGAGTGCATTGCCTGAACCCGGAGTCAGCCTCCTTCGCACGACTTGTGGGACCCCTAACTATGTTGCACCTGAGGTTCTCAGTCACAAGGGTTATGATGGTGCCGTGGCAGATGTTTGGTCCTGCGGGGTCATCCTGTATGTTCTCATGGCTGGATACCTTCCATTTGATGAGCTTGACCTGACCACATTATATAAGGATGATTCTTCCGATGAGGGACATAATGACATATGCAAATTTGATGGTGGTGTTTTTGCAGCATCTAAGGAGGATGACAAGTTGCATTCAAAAAGAATGTGTTTTTTCCAGCGTATTACAATTCAACAGATCAGAAATGATGAGTGGTTTCAGAAAAATTATGTTCCTCCCAAAATATTAGAATATGAAGATGTAAATCTTGATGATGTAAATGCTGTCTTTGACGAGACCGAGGAAGAAAGGGATAGCGAGCACCAGGGAAGTGAGGACATGGGTCCTTTAGTTCTTAATGCATTTGACTTGATCATCTTATCTCAAGGCCTGAACCTGGCGTCAATGTTTGACCGTGGCAAGGATTGTATGAAGTATCAGACTCGCTTTGTCTCACAAAAGCCAGCAAAGGTCGTACTATCAAGCATGGAAGTTGTTGCACAATCAATGGGTTTCAAGACACACATCCGCAACTATAAGATGCGAGTGGAGGGTCTTTCGGCAAATAAAACTTCTCATTTCTCCATTATTCTCGAAATTTTCGAAGTTGCTCCAACCTATTATATGGTAGACATTCAGAAAGCTGCTGGAGATGCTAGTGAATTCCTCAAGTGTACTGCTTTATCTTTAGTTTCTGATTCCCTACTGTTCTACAGTTTTACAAGAACTTTTGTAGCAATCTTGAGGATATCATCTGGAAACCGCCTACTGAATCTAGCAAATCAAGGATCAGCAAGAGTAAAAGTAGAAAGTGTTGATATCTTACACAGCTGCTTTCACTCCACGGTCATTGCTGGACTTGTACTTGACAACGCTTTTGGAGGCATGGGGCATTAA

>FvSnRK3.16

ATGGACAAGGAGGGGGAGGTGTTGCTGGGGAAGTACCAACTTGGGAAGCTCTTAGGGCAGGGGGGCTTTGCAAAGGTCTACCACGCAAGGAGCCTCAAAACCAACCAGATTGTAGCCATTAAGATCATCAGCAAAGAGAAGGTTTTTGAATTGGGACTCGTTGATCAGACCAAAAGAGAGATTTCTATTATGAGACTGCTTAAACACCCCAACATAGTGCAGCTATATGAAGTCATGGCCACCAAAAAGAAGATCTACCTTGTCATGGAATACGCAGAAGGCGGCGAGCTTTTTCAAAAGATAAATAAAAGGAGGCTCAAGGAGGAAGCAGCAAGGAGATTCTTCCAACAACTCATCACTGCTGTCGACTTCTGCCACAAGAGAGGTGTTTTCCATCGCGATTTGAAGCCCGAGAACTTGCTGCTAGATAAAGATGGAGTGCTGAAGGTGTCGGATTTTGGGTTGAGTGCGTTTTCTGAATCGAAGAGGAAACATGCTTTACTGCATACAACTTGTGGCACTCCTAATTATGTAGCTCCTGAAGTTATCCGGCTTGGAGCCTATGATGGAGCTAAAGCTGATATATGGTCTTGTGGGGTGATCTTGTTTCAACTATTGGCTGGTTATCGTCCCTTCGATGACTCAAATCTAAACAACATGTTTAGGAAAATATGTGCATCAGAATATCGATGTCCCCGGTGGTTTTCCGATGACATAAGAAAGCTCTTGTTTGGAATCCTCAACACAAATCCGAATGAAAGGTTTCTTGCTTCAGACATAATGAGAAGTAGCTGGTTTCAAGAAGGACTCAGCTCCAAGATTAAAACAGAAGTGGAAGATGTTGATGGGGAGTCTGATGACTGTGACAAAAGCGAGAACCAAGAGACTATTACACCCGCCACTTTGAGTGCTTTCGATATCATATCTCTCTCTAGTGGATTCGATCTTTCTGGCTTGATGATGCAGAAGGATGCAAAGAGATCAGCTGTGCAGTTCACATCCGCGCAATCTGCGACATCCATCATGACAAAGCTGAGGGATATTACTCGGAAACTGAAGCTGAAATCGAAGAAAGAAGGAGCATCATTGAAGTTAATGAAGGGGGCATTATCCATAGAAGCCGAAATTTTTGAGTTCACTCCATCCTTTCATTTAGTGGAGATGAAGAAATCTAATGGGGATACATTTGAGTTTAGGAAGATGGTAGACGAGGATATAAGGCCGGCTCTCAAAGACGTCGTCTGGACATGGCAGGGTGAGCGCAGTAACAACAATAGCAGCATCTGCGTTTGA
